# Personalised lung cancer risk stratification and lung cancer screening: do general practice electronic medical records have a role?

**DOI:** 10.1038/s41416-023-02467-9

**Published:** 2023-10-25

**Authors:** Bhautesh Dinesh Jani, Michael K. Sullivan, Peter Hanlon, Barbara I. Nicholl, Jennifer S. Lees, Lamorna Brown, Sara MacDonald, Patrick B. Mark, Frances S. Mair, Frank M. Sullivan

**Affiliations:** 1 General Practice and Primary Care, School of Health and Wellbeing, University of Glasgow, Glasgow, UK; 2 School of Cardiovascular and Metabolic Health, University of Glasgow, Glasgow, UK; 3 Population and Behavioural Science Division, School of Medicine, University of St Andrews, St Andrews, UK

**Keywords:** Lung cancer, Epidemiology

## Abstract

**Background:**

In the United Kingdom (UK), cancer screening invitations are based on general practice (GP) registrations. We hypothesize that GP electronic medical records (EMR) can be utilised to calculate a lung cancer risk score with good accuracy/clinical utility.

**Methods:**

The development cohort was Secure Anonymised Information Linkage-SAIL (2.3 million GP EMR) and the validation cohort was UK Biobank-UKB (*N* = 211,597 with GP-EMR availability). Fast backward method was applied for variable selection and area under the curve (AUC) evaluated discrimination.

**Results:**

Age 55–75 were included (SAIL: *N* = 574,196; UKB: *N* = 137,918). Six-year lung cancer incidence was 1.1% (6430) in SAIL and 0.48% (656) in UKB. The final model included 17/56 variables in SAIL for the EMR-derived score: age, sex, socioeconomic status, smoking status, family history, body mass index (BMI), BMI:smoking interaction, alcohol misuse, chronic obstructive pulmonary disease, coronary heart disease, dementia, hypertension, painful condition, stroke, peripheral vascular disease and history of previous cancer and previous pneumonia. The GP-EMR-derived score had AUC of 80.4% in SAIL and 74.4% in UKB and outperformed ever-smoked criteria (currently the first step in UK lung cancer screening pilots).

**Discussion:**

A GP-EMR-derived score may have a role in UK lung cancer screening by accurately targeting high-risk individuals without requiring patient contact.

## Background

Lung cancer is a leading cause of global cancer incidence and cancer-related mortality with close to 2 million cases in 2020 [1]. In the UK, lung cancer is the third most common cancer and the most common cause of cancer mortality [2]. Computerised Tomography (CT) for lung cancer screening has been shown to reduce the risk of lung cancer mortality [3]; notably 20% reduction in the National Lung Screening Trial (NLST) trial [4] and 24% reduction in the Dutch–Belgian Lung Cancer Screening Trial (NELSON), respectively [5].

Lung cancer screening trials using CT scans have mainly used two risk factors, age and smoking history, in their inclusion criteria for the identification of high-risk populations [4–8]. The United States Preventive Services Task Force (USPTF) uses a ‘*pack-year criteria’* and recommends lung cancer screening between age 50–80 years for those who were smokers within the past 15 years and who have a smoking history of 20 pack years or more [9]. Despite this, only 5–18% of eligible patients are being screened [10]. In recent years, various lung cancer risk prediction models have been developed and found to have high discriminatory power in identifying those with a high risk of lung cancer [3, 11]. Commonly included risk factors in these models are comprehensive information on smoking history (duration, quit time and intensity), family history of lung cancer and lifetime asbestos exposure [3, 11]. Implementation of the existing lung cancer risk prediction models into routine clinical practice would necessitate patient contact as electronic medical records (EMR) are unlikely to have the required granularity of information on component risk factors [12–14].

A general practice (GP) electronic health records-based risk score is important in the UK healthcare setting context as all existing cancer screening programmes (cervical, breast and bowel) in the UK are based on general practice registrations (https://www.cancerresearchuk.org/about-cancer/cancer-symptoms/spot-cancer-early/screening/what-is-cancer-screening). In the UK, lung cancer screening ‘pilots’-Targeted Lung Health Check (TLHC) projects for high-risk individuals have been implemented in certain areas by NHS England [15, 16]. High-risk participants are identified by a two-step process and those eligible on assessment are offered low-dose CT screening [15]. The first step involves identifying participants who have ever-smoked, those registered with a GP practice and those aged between 55 and 75 years, using the general practice EMR. The second step involves a comprehensive assessment, which includes a spirometry test and a discussion to assess participants’ individual lung cancer risk. The Lung Health Check programme uses two of the existing lung cancer risk prediction models for step two: Prostate Lung Colorectal and Ovarian (PLCO)_M2012_ and Liverpool Lung Project version 2 (LLPv2) [17]. Participants found to have a risk threshold of ≥1.51% risk of lung cancer over 6 years as the minimum threshold for PLCO_M2012_; and ≥2.5% risk of lung cancer over 5 years for LLPv2 in step two are eligible for a low-dose CT screening [3, 11, 15]. EMRs have been used to predict lung cancer risk in symptomatic patients [18], and other studies in the general population are underway [19]. In the UK context, cancer screening is based on general practice registrations and in pilot studies for lung cancer screening, only every-smoked criteria has been extracted from GP records for identifying those at high risk.

We hypothesize that general practice EMRs can improve the identification of individuals at high risk of lung cancer compared to the ever-smoked criteria by extracting sociodemographic, lifestyle factors and long-term conditions prevalence. The study objectives are to calculate a lung cancer risk prediction model from a routinely collected general practice data source; validate the new model in another data source; assess discrimination and calibration of the new model; and compare the new model accuracy against ever-smoked criteria and other commonly used lung cancer risk prediction models (PLCO_M2012_ and LLPv2).

## Methods

### Data sources and study population

The development cohort was identified from Secure Anonymised Information Linkage (SAIL Databank). SAIL databank has data from primary care EMRs from Wales and covers ~70% of the population of Wales, representative of the wider population in terms of age, sex, and socioeconomic deprivation [20, 21]. We identified all participants that were currently registered with a participating practice on January 1, 2011 and who had been registered for a full year prior to this date, as electronically coded data was most complete from this point [21]. These data were regarded as the start of the follow-up period for SAIL analysis. The validation cohort was UK Biobank, a population-based cohort study which includes 502,640 participants enrolled from 22 different centres across England, Scotland, and Wales between 2006 and 2010 (5% response rate). At present, linked primary care data are only available for a subset of participants. The availability of primary care data depended on the electronic medical record system used by the practice (data only currently available from certain systems) rather than any participant-level factors. This subset is representative of the UK Biobank cohort [22]. The date of recruitment for a participant to UK Biobank was regarded as the start of the follow-up period. The age range for SAIL was 55 to 75 years while in UK Biobank it was 55–73 years as that was the maximum age of participants recruited to UK Biobank. Participants with a previous history of lung cancer (based on lung cancer registry records) were excluded in both cohorts.

### Predictor and outcome variables

Age at baseline was used as a continuous variable in both datasets. Sex was used as a categorical variable. The Welsh Index for Multiple Deprivation (WIMD) score [23] in SAIL and Townsend score [24] in UK Biobank, respectively, were used to measure socioeconomic status and the scores were divided into quintiles. WIMD is the Welsh Government’s official measure of relative deprivation for small areas in Wales. It identifies areas with the highest concentrations of several different types of deprivation. WIMD ranks all small areas in Wales from most deprived to least deprived [23]. The Townsend Deprivation Index is a measure of material deprivation first introduced by Peter Townsend in 1987. A Townsend score can be calculated using a combination of four census variables for any geographical area (provided census data is available for that area): households without a car, overcrowded households, households not owner-occupied, persons unemployed [24]. A previous study has found association between Townsend score and lung cancer risk among symptomatic patients attending GP practices in the UK [18]. In both datasets, smoking status was defined using general practice Read codes and divided into three categories: non-smokers, previous smokers, and current smokers, using Read codes from previously published studies [12] (please see Supplementary File Table S1 for read codes used). There was a considerable heterogeneity in the duration between recording of the smoking status and the start of the follow-up period across the two cohorts. Supplementary Table S2 shows the median duration (with interquartile range) across different smoking categories in the two cohorts.

We used a previously published list of 40 long-term conditions (LTCs), defined using Read codes in SAIL as predictors [21, 22] (please see Supplementary File Table S2 for Read2 and CTV3 codes). In addition, we also considered previously identified risk factors for lung cancer by PLCO_M2012_ and LLPv2 as candidate predictor variables. Painful conditions as a LTC included a broad group of conditions which included back pain, joint pain, headaches (not migraine), sciatica, plantar fasciitis, carpal tunnel syndrome, fibromyalgia, arthritis, shingles, disc problem, prolapsed disc/slipped disc, spine arthritis/spondylitis, ankylosing spondylitis, back problem, osteoarthritis, gout, cervical spondylosis, trigeminal neuralgia, and disc degeneration, using previously validated definitions [25–27].

In UK Biobank, participants also self-reported/underwent an examination for information on family history of lung cancer, detailed history on smoking status and body mass index, while in SAIL all candidate predictor variables were extracted from general practice records. The outcome of interest was lung cancer incidence at 6 years of follow-up, which was derived by searching for the presence and date of ICD-10 code “C34” within linked cancer registry records in both cohorts.

### Statistical analysis

All statistical analysis was performed using R version 4.0.3 in R studio. For the development of lung cancer risk prediction model in the SAIL cohort, fast backward variable selection model (using *P* value < 0.01 as the significance level for selecting variables in the model) from rms package was used [28]. Age, sex, socioeconomic status, smoking status, and presence/absence of *N* = 40 LTCs were entered as predictor variables. In addition, we also considered previously identified risk factors for lung cancer by PLCO_M2012_ and LLPv2 as candidate predictor variables. The association of selected variables (by backward selection) with 6-year lung cancer risk was examined using logistic regression models in both development (SAIL) and validation cohorts (UK Biobank). Interaction between selected variables and smoking status was tested and if a significant statistical interaction was found, this was added to the final risk score. The discriminating ability of the newly developed EMR-based lung cancer risk score in the prediction of lung cancer was compared to ever-smoked criteria from the TLHC programme [15] in the SAIL and the UK Biobank cohorts using receiver operating characteristics (ROC) area under curve (AUC) [29]. Similarly, AUC was used to compare the EMR-based risk score against the PLCO_2012_ [30] and LLPv2 [17] in both cohorts. The calibration performance of the new EMR-based lung cancer risk score was evaluated using cut-off values at each 10% increment and compared against the ever-smoked criteria. This was done in both development and validation cohorts. The number of false positive, true positive, true negative and false negative cases were reported for each threshold. In addition, sensitivity, specificity, positive and negative predictive values, and balanced accuracy (formula= sensitivity + specificity/2) were also reported for each threshold [31]. The number of ever-smokers excluded, the number needed to detect one lung cancer and the number correctly excluded for every lung cancer missed were also reported for each 10% increments in both cohorts. The number needed to screen to detect one lung cancer was calculated using the formula: (false positive + true positive)/true positive cases. The number correctly excluded for every lung cancer case missed using the formula: (true negative+ false negative)/false negative. The calibration performance was compared against ever-smoked criteria (yes/no) and PLCO_2012_ ≥ 1.51% threshold [15].

### Sensitivity analysis

The AUC analyses were repeated for the general practice EMR score and other comparator scores described above after including data from hospital admissions (in addition to general practice health records) for calculating LTC prevalence using a previously validated algorithm [32].

### Sub-group analysis

The SAIL cohort was split equally into ten sub-groups for internal validation and the AUC was calculated independently to assess the discriminating power of the new score in predicting lung cancer incidence at 6 years. In addition, a sub-group analysis was conducted in SAIL cohort based on age group, sex and smoking status.

## Results

### Study populations

**Fig. 1:**
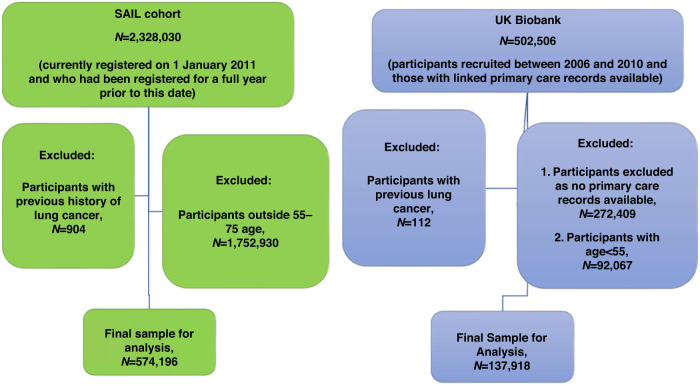
Study population for the development cohort (SAIL) and validation cohort (UK Biobank). SAIL secure anonymised information linkage.

In the development cohort (SAIL), ~2.3 million patients were registered with a GP practice at the start of the follow-up period (January 1, 2011). The study population was *N* = 574,196, aged between 55 and 75 years, after excluding those with previous history of lung cancer. The incidence of lung cancer was 1.11% (6430 cases) at 6 years follow-up. In the validation cohort (UK Biobank), *N* = 137,918 met the eligibility criteria and had primary care records available, with a lung cancer incidence of 0.48% (656 cases) at 6 years (see Fig. 1 for details).

Table 1 describes demographic factors and smoking habits in both cohorts. SAIL cohort had a proportionately higher number of current smokers (*N* = 156,463; 27.25%) compared to current smokers recorded in the UK Biobank cohort (*N* = 19,247; 13.95%). While the UK Biobank cohort had a higher proportion of ex-smokers (*N* = 44,722; 32.42%) compared to ex-smokers recorded in the SAIL cohort (*N* = 123,274; 21.47%).

**Table 1:** Study participant demographics, smoking status and 6-year lung cancer incidence.

SAIL cohort; age range 55–75			
*N* = 574,196	No lung cancer, *N* = 567,766 (98.89%)	Lung cancer, *N* = 6430 (1.11%)	*P* value for difference
Age in years: mean (SD) missing values = 0	64.0 (6.1)	66.5 (5.7)	<0.001
Gender, missing = 9			<0.001
Males 291,772	278951 (98.7%)	3464 (1.3%)	
Females 282,415	288,806 (98.9%)	2966 (1.1%)	
WIMD quintiles missing=32,865			<0.001
Q1 120,179 affluent	119,364 (99.4%)	815 (0.6%)	
Q2 107,182	106,227 (99.1%)	955 (0.9%)	
Q3 115,736	114,459 (98.9%)	1277 (1.1%)	
Q4 102,298	100,885 (98.6%)	1413 (1.4%)	
Q5 95,936 deprived	94,322 (98.3%)	1614 (1.7%)	
Smoking status; missing=12,037			<0.001
Non-smoker 282,422	281,729 (99.8%)	693 (0.2%)	
Ex-smoker 123,724	121,891 (98.8%)	1383 (1.2%)	
Current smoker 156,463	152,269 (97.3%)	4194 (2.7%)	
**UK Biobank cohort; age range 55–73**			
*N* = 137,918	No lung cancer *N* = 137,262 (99.52%)	Lung cancer *N* = 656 (0.48%)	
Age in years: mean (SD), missing values = 0	62.0 (4.1)	63.4 (4.0)	<0.001
Gender, missing=0			0.002
Males 63,517	63,174 (99.4%)	343 (0.5%)	
Females 74,401	74,088 (99.6%)	313 (0.4%)	
Townsend quintiles, missing=0			<0.001
Q1 29,112 Affluent	29,023 (99.7%)	89 (0.3%)	
Q2 29,223	29,124 (99.7%)	99 (0.3%)	
Q3 29,476	29,366 (99.6%)	110 (0.4%)	
Q4 26,684	26,538 (99.4%)	146 (0.6%)	
Q5 23,423 deprived	23,211 (99.1%)	212 (0.9%)	
Smoking status; missing=0			<0.001
Non-smoker 73,949	73,723 (99.7%)	226 (0.3%)	
Ex-smoker 44,722	44,551 (99.6%)	171 (0.4%)	
Current smoker 19,247	18,988 (98.6%)	259 (1.4%)	

*SAIL* secure anonymised information linkage, *WIMD* Wales index for multiple deprivation.

WIMD and Townsend score are area-based measures of socioeconomic status [23, 24].

Compared to those who did not develop lung cancer, people developing lung cancer over 6 years were older in both cohorts. In both cohorts, males, current and ex-smokers and participants from socioeconomically deprived areas were observed to have a higher lung cancer incidence at 6 years. Smoking status captured by primary care records and that captured by self-report in UK Biobank had moderate correlation with Spearman correlation coefficient value of 0.405 (95% CI 0.401–0.409).

### Development and validation of lung cancer risk score

Variable selection, using backward selection method, retained 17/56 predictor variables in the SAIL cohort (age, sex socioeconomic status, smoking status, family history of lung cancer, body mass index (BMI), BMI: smoking status, and presence of the following LTCs: alcohol misuse, chronic obstructive pulmonary disease (COPD), coronary heart disease, dementia, hypertension, painful condition, stroke/transient ischaemic attack (TIA), peripheral vascular disease, and history of previous cancer and previous pneumonia). The newly developed lung cancer risk score was called ALIGNED (generAL practIce lunG caNcEr moDel-aligned). The full results of variable selection are presented in Supplementary Table S4 and the results for interaction testing between selected variables and smoking status are presented in Supplementary Table S5. The relationship of these predictor variables with the 6-year risk of lung cancer incidence was assessed using multivariate logistic regression models in the development and validation cohorts, respectively, and presented in Table 2. The presence of ten LTCs was associated with a significantly higher risk of lung cancer at 6 years in SAIL cohort and five LTCs (COPD, painful condition, hypertension, peripheral vascular disease and history of previous cancer) had a significant association in both cohorts.

**Table 2:** Variables selected in the final model and association with 6-year lung cancer risk: multivariate logistic regression analysis.

	OR with 95% CI- SAIL (development cohort), *N* = 574,196	OR with 95% CI-UK Biobank (validation cohort), *N* = 137,918
Age-continuous	1.07 (1.06–1.08)	1.06 (1.04–1.09)
Sex—male	1.09 (1.03–1.15)	1.24 (1.04–1.48)
Socioeconomic status		
SE Quintile 1 least deprived	Reference	Reference
SE Quintile 2	1.14 (1.03–1.25)	1.16 (0.83–1.63)
SE Quintile 3	1.27 (1.17–1.40)	1.24 (0.89–1.73)
SE Quintile 4	1.39 (1.27–1.51)	1.94 (1.44–2.65)
SE Quintile 5 most deprived	1.48 (1.36–1.62)	2.51 (1.88–3.39)
Family history of lung cancer	1.84 (1.38–2.38)	1.66 (1.35–2.01)
Smoking status		
Never smoked	Reference	Reference
Previous smoker	3.86 (3.49–4.28)	1.52 (0.38–6.06)
Current smoker	9.94 (9.08–10.89)	9.17 (2.66–31.40)
Body mass index (BMI)-continuous	1.02 (1.01–1.03)	
Presence of LTCs		
Alcohol misuse	1.31 (1.14–1.48)	1.44 (0.84–2.35)
Coronary heart disease	1.12 (1.02–1.21)	0.97 (0.69–1.32)
Chronic obstructive pulmonary disease	2.01 (1.89–2.13)	2.53 (1.96–3.23)
History of previous cancer	1.37 (1.26–1.47)	1.40 (1.07–1.81)
Peripheral vascular disease	1.27 (1.16–1.38)	1.71 (1.23–2.32)
Dementia	0.40 (0.22–0.65)	No output*
Painful condition	1.14 (1.07–1.21)	1.34 (1.09–1.63)
Hypertension	0.91 (0.85–0.95)	1.25 (1.04–1.50)
Stroke/transient Ischaemic attack	1.18 (1.06–1.30)	1.41 (0.94–2.04)
History of previous pneumonia	1.38 (0.73–1.23)	0.71 (0.30–1.39)
BMI: Previous smoker interaction	0.99 (0.98–0.99)	0.98 (0.94–1.03)
BMI: Current smoker interaction	0.98 (0.97–0.99)	0.96 (0.92–1.01)

*SAIL* secure anonymised information linkage, *OR* odds ratio, *CI* confidence intervals.

WIMD and Townsend score were area-based measures of socioeconomic status in SAIL and UK Biobank, respectively. *No lung cancer cases were recorded in participants with dementia in the UK Biobank cohort.

### Discrimination and calibration of ALIGNED score

**Fig. 2:**
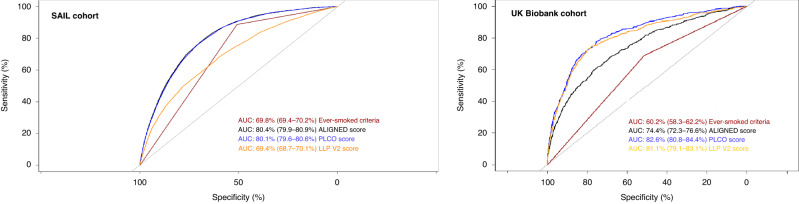
Area under curve (AUC) of the new EMR-based lung cancer ALIGNED score for 6-year lung cancer incidence prediction. ALIGNED score generAL practIce lunG caNcEr model, PLCO Prostate Lung Colorectal and Ovarian PLCOM2012, LLP V2 Liverpool Lung Project version 2 (LLPv2). Left panel = findings in the development cohort (SAIL secure anonymised information linkage); right panel = findings in the validation (UK Biobank) cohort.

The discriminating power of ALIGNED in 55–75-year-olds to detect 6-year risk of lung cancer was good, with AUC value of 80.4% in the development (SAIL) cohort (95% CI 79.9–80.9%). The discriminating power (AUC value) of ever-smoked status (yes/no) and PLCO_2012_ in the SAIL cohort for 55–75-year-olds was A 69.8% (95% CI 69.4–70.2%) and 80.1% (95% CI 79.6–80.6%), respectively, see Fig. 2. Table 3 reports calibration performance of the ALIGNED score using cut-off scores at each 10% decile value in SAIL. In SAIL, 70% threshold cut-off had the best value for balanced accuracy at 73.3%, while 50%, 60 and 80% thresholds, respectively, also performed better than the ever-smoked criteria (balanced accuracy=69.8%). The number needed ‘to screen’ one lung cancer case was 35, and for every 262 participants correctly excluded, one true lung cancer would be missed, with the 70% threshold of ALIGNED in SAIL (the most accurate threshold). In comparison, the number needed to screen one lung cancer case was 50 for ever-smoked criteria and 77 for PLCO_2012_ ≥ 1.51% in the SAIL cohort.

**Table 3:** Model discrimination and calibration for different cut-off values across each decile of the ALIGNED risk score and comparison against ever-smoker status in SAIL.

Deciles	10%	20%	30%	40%	50%	60%	70%	80%	90%	Ever-smoked (yes/no) as predictor	PLCO_M2012_ ≥ 1.51% (yes/no) as predictor
True negative	52,976 (10%)	105,907 (20%)	158,807 (30%)	211,696 (39.9%)	264,495 (49.9%)	317,173 (59.8%)	**369,623 (69.7%)**	421,856 (79.6%)	473,582 (89.3%)	266,562 (50.2%)	77,921 (14.7%)
False positive	471,170 (88.9%)	418,239 (78.9%)	365,339 (68.9%)	312,450 (58.9%)	259,651 (49%)	206,973 (39.0%)	**154,523 (29.1%)**	102,290 (19.3%)	50,564 (9.5%)	257,584 (48.5%)	446,225 (84.2%)
False negative	36 (0.007%)	113 (0.02%)	210 (0.04%)	329 (0.06%)	542 (0.1%)	870 (0.2%)	**1416 (0.3%)**	2199 (0.4%)	3480 (0.7%)	666 (0.1%)	71 (0.01%)
True positive	5887 (1.11%)	5810 (1.09%)	5713 (1.07%)	5594 (1.05%)	5381 (1.1%)	5053 (0.9%)	**4507 (0.8%)**	3724 (0.7%)	2443 (0.5%)	5257 (1.0%)	5852 (1.1%)
Balanced accuracy	54.7%	59.1%	63.3%	67.4%	70.6%	72.9%	**73.3%**	71.6%	65.8%	69.8%	56.8%
Sensitivity	10.1%	20.2%	30.3%	40.3%	50.4%	60.5%	**70.5%**	80.4%	90.3%	50.8%	14.8%
Specificity	99.3%	98.1%	96.4%	94.4%	90.8%	85.3%	**76.1%**	62.8%	41.2%	88.7%	98.8%
PPV	99.9%	99.8%	99.8%	99.8%	99.8%	99.7%	**99.6%**	99.5%	99.3%	99.7%	99.9%
NPV	1.2%	1.3%	1.5%	1.7%	2.0%	2.4%	**2.8%**	3.5%	4.6%	2.0%	1.3%
Number needed to screen to detect one lung cancer case	81	72	65	56	49	41	**35**	28	21	50	77
Number correctly excluded for every lung cancer case missed	1472	938	757	644	488	365	**262**	192	137	401	1098
Number of ever-smokers excluded	0	3	19	1537	16,777	55,017	**10,4843**	157,080	209,862	Not applicable	0

*SAIL* secure anonymised information linkage, *ALIGNED* generAL practIce lunG caNcEr moDel, *PPV* positive predictive value, *NPV* negative predictive value, *SD* standard deviation.

*N* = 530,069, lung cancer at 6 years = 5923 (1.11%); no lung cancer = 524146 (98.89%) (after excluding participants with missing values).

Number needed to screen to detect one lung cancer=False positive+ True Positive/True positive. Number correctly excluded for every lung cancer case missed=True negative+ False negative/False negative. Results in bold (70% threshold) indicates the best balanced accuracy values.

In the validation cohort (UK Biobank), the ALIGNED score (AUC 74.4%; 95% CI 72.3–76.6%) outperformed the ever-smoked criteria (AUC 60.2%; 95% CI 58.3–62.2%) in 55–73 years old participants (please see Fig. 2). However, the ALIGNED score underperformed PLCO_M2012_ (AUC 82.6%; 95% CI 80.8–84.4%) and LLPv2 (AUC 81.1%; 95% CI 79.1–83.1%) in the UK Biobank cohort. Of note, the PLCO_M2012_ and LLPv2 in UK Biobank was calculated using smoking information which was self-reported by the participants at the time of the recruitment. In the calibration analysis, six thresholds of the ALIGNED score from 30% onwards outperformed the ever-smoked criteria with better-balanced accuracy (see Table 4). In this cohort as well, 70% threshold of the ALIGNED score offered the best-balanced accuracy of 68% and it significantly outperformed the ever-smoked criteria (balanced accuracy=60.2%). The number needed ‘to screen’ one lung cancer case was 80, and for every 363 participants correctly excluded, one true lung cancer would be missed, with the 70% threshold of ALIGNED in UK Biobank. In both datasets, the ALIGNED score had high positive predictive values but low negative predictive values. The number of patients needed to be screened to detect one patient with lung cancer was comparatively smaller for SAIL across all the decile cut-off scores, which is expected as SAIL is the more representative cohort of the general population with higher lung cancer incidence.

**Table 4:** Model discrimination and calibration for different cut-off values across each decile of the ALIGNED risk score and comparison against ever-smoker status in UK Biobank.

Deciles	10%	20%	30%	40%	50%	60%	70%	80%	90%	Ever-smoked (yes/no) as predictor	PLCO_M2012_ ≥ 1.51% (yes/no) as predictor
True negative	9648 (10%)	19,296 (19.9%)	28,937 (29.9%)	38,574 (39.9%)	48,209 (49.9%)	57,825 (59.9%)	**67,445 (69.8%)**	77043 (79.7%)	86,617 (89.6%)	49,536 (51.3%)	45,408 (47%)
False positive	86,424 (89.4%)	76,776 (79.5%)	67,135 (69.5%)	57,498 (59.5%)	47,863 (49.5%)	38,247 (39.6%)	**28,627 (29.6%)**	19,029 (19.7%)	9455 (9.8%)	46,536 (48.2%)	50,664 (52.4%)
False negative	14 (0.01%)	28 (0.03%)	48 (0.04%)	72 (0.07%)	99 (0.1%)	144 (0.1%)	**186 (0.2%)**	247 (0.3%)	329 (0.3%)	169 (0.2%)	51 (0.05%)
True positive	530 (0.54%)	516 (0.53%)	496 (0.51%)	472 (0.48%)	445 (0.45%)	400 (0.41%)	**358 (0.37%)**	297 (0.3%)	215 (0.22%)	375 (0.39%)	493 (0.5%)
Balanced accuracy	53.7%	57.4%	60.6%	63.4%	66%	66.8%	**68%**	67.3%	64.8%	60.2%	68.9%
Sensitivity	10%	20.1%	30.1%	40.1%	50.1%	60.2%	**70.2%**	80.2%	90.1%	51.5%	47.2%
Specificity	97.4%	94.8%	91.1%	86.7%	81.8%	73.7%	**66.2%**	54.6%	39.5%	68.9%	90.6%
PPV	99.8%	99.8%	99.8%	99.8%	99.7%	99.7%	**99.7%**	99.6%	99.6%	99.7%	99.8%
NPV	0.6%	0.6%	0.7%	0.8%	0.9%	1.0%	**1.2%**	1.5%	2.2%	0.8%	1.0%
Number needed to screen to detect one lung cancer case	164	149	136	122	108	96	**80**	65	44	125	103
Number correctly excluded for every lung cancer case missed	690	690	603	536	487	402	**363**	312	264	294	877
Number of ever-smokers excluded	2314	5401	8728	12,274	16,160	20,459	**25,266**	30,836	38,012	Not applicable	15,505

*ALIGNED* generAL practIce lunG caNcEr moDel, *PPV* positive predictive value, *NPV* negative predictive value, *SD* standard deviation.

*N* = 96,616, lung cancer at 6 years = 544 (0.56%), no lung cancer = 96072 (99.44%) (after excluding participants with missing values)

Number needed to screen to detect one lung cancer=False positive+ True Positive/True positive; Number correctly excluded for every lung cancer case missed=True negative+ False negative/False negative. Results in bold (70% threshold) indicates the best balanced accuracy values.

### Sensitivity analysis

We checked the accuracy of ALIGNED score after excluding patients with dementia. The AUC for both SAIL and UKB cohorts were relatively unchanged (80.3% and 74.4%, respectively) in the sensitivity analysis. The lung cancer risk scores were recalculated using hospital admission data (in addition to GP data) which improved the AUC values for ALIGNED, PLCO_M2012_ and LLPv2 scores in both SAIL and UK Biobank cohorts (please see Supplementary Material Table S6). In addition, the AUC for risk scores were recalculated for lung cancer incidence at 5 years in SAIL and the result trends remain unchanged (please see Supplementary Table S7).

### Sub-group analysis

SAIL cohort data was split into ten equal parts, the AUC values for ALIGNED score ranged from 79.1 to 81.6% across the ten sub-groups (please see Supplementary Material Table S8). In sub-group analysis based on demographic characteristics and smoking status, ALIGNED score had the highest AUC value of 81.1% among females and lowest among current smokers at 67.1% (please see Supplementary Table S9).

## Discussion

This study presents the findings of developing and validating a GP EMR-based lung cancer risk score—ALIGNED—from two large community cohorts. The new score was based on using demographic information (age, sex, and socioeconomic status), smoking status (non-smoker, ex-smoker and current smokers), body mass index, family history of lung cancer and presence of following LTCs: alcohol misuse, COPD, coronary heart disease, dementia, hypertension, painful condition, stroke/TIA, peripheral vascular disease, and history of previous cancer and previous pneumonia. The new score outperformed the ever-smoking status from TLHC in discrimination and calibration, however underperformed against the questionnaire-derived PLCO_M2012_ and LLPv2 risk scores. The new ALIGNED score may have a potential role in implementation of lung cancer screening at population level, crucially without requiring patient contact, while the other commonly used scores like PLCO_M2012_ and LLPv2 rely on information which require participant response.

A proactive approach based on individual risk prediction is being described as the future of early cancer detection, across all cancers [33]. A risk prediction model-based approach in lung cancer screening has been found to be associated with greater reduction in lung cancer mortality in clinical trials [34]. Use of EMR for lung cancer prediction has been previously examined in a lung cancer screening trial [8] and routine implementation of lung cancer screening programmes [35, 36], however, these studies have not used the EMR to generate a lung cancer risk score. A personalised lung cancer risk score generated from the EMR could potentially help with reducing perceived cancer risk—a recognised barrier in lung cancer screening participation [37, 38]—and enable those at higher risk to be provided with a personalised risk estimation. Identifying high-risk individuals at an early stage via EMR can pave the way for more personalised targeting of high-risk individuals for the next stage of risk assessment (including comprehensive smoking and family history, spirometry, +/− biomarkers/imaging) [39]. Importantly, EMR-based lung cancer risk score can be calculated without any patient contact which means that everyone can be assessed, not just those who attend for a complete assessment, which has the potential to reduce health inequalities.

We compared cumulative lung cancer incidence and AUC values in our cohorts with those from previous studies. The observed 6-year lung cancer cumulative incidence among ever-smokers was 5257 (2%) in SAIL (age 55–75 years) and 375 (0.8%) in UK Biobank (age 55–73 years), respectively. In comparison, cumulative lung cancer incidence was 0.85% (46/4380) at 5.5 years in the NELSON trial cohort [40], 0.80% (599/75,958) at 5 years in UKLS trial cohort [41], 1.84% (1463/79,209) in PLCO trial cohort and 3.73% (1925/51,527) in NLST trial cohort respectively at 6 years [11]. In our study, the new EMR-based lung cancer risk score had an AUC value of 80% (SAIL) and 74% (UK Biobank). Previous studies have reported AUC values of 67%, 73%, 73%, 74 and 81% for LLP V2 risk score in different cohorts, respectively [11, 41, 42]. Similarly, reported AUC values for PCO_M2012_ risk score have been 68%, 74%, 76 and 78%, respectively, in different cohorts [11, 42]. The ALIGNED score includes LTCs which have not been previously included in any previous lung cancer risk scoring system, however, previous research has found similar associations. A higher risk of lung cancer has been observed with heavy alcohol consumption: in particular, beer drinking was associated with higher risk of squamous cell carcinoma [43, 44]. A meta-analysis of 16,849 patients found a higher prevalence of lung cancer among those with peripheral vascular disease [45]. A cohort study in Germany involving 18,668 found a higher risk of intrathoracic cancers among stroke survivors, in both men and women [46].

Using a large primary care representative cohort for development (SAIL) and another large community cohort (UK Biobank) for validation is one of the key strengths of this study. However, we acknowledge some limitations, particularly relating to the UK Biobank cohort. UK Biobank participants have been found to be less socioeconomically deprived, have fewer lifestyle risk factors and lower prevalence of LTCs than the UK population [47]. However, despite lower absolute risk of lung cancer and other diseases, hazard ratios derived from UK Biobank should still be applicable to the wider UK population [48]. There was significant heterogeneity in the duration between recording of smoking status in GP EMR and the start of the study follow-up across different smoking categories in the two cohorts which would have likely influenced the accuracy of smoking status considered in both cohorts. In this study, we could not use free text data available in GP EMR for extracting more detailed information on predictor variables, including smoking behaviour as free text data was not available for research purposes in both these cohorts. Use of free text data and natural language processing methods have the potential to improve the prediction power and accuracy of lung cancer risk. The correlation between GP EMR-based classification and self-reported classification of smoking status had moderate correlation so there is a possibility of misclassification and measurement error when using GP records for capturing smoking status.

A GP EMR-based lung cancer risk score-LUCGPEHR was validated using two large UK community cohorts and comprised of demographic information (age and socioeconomic status), smoking status, body mass index, family history of lung cancer, and presence/absence of ten LTCs. The crucial benefit of the ALIGNED score is not requiring patient contact for score calculation. The ALIGNED score outperformed the ever-smoked criteria used in the TLHC programme. The ALIGNED score may have a potential role as a “first step” in the implementation of lung cancer screening by facilitating more informed decision-making with personalised information available for participants. It can also be potentially used for improving screening precision by more focused targeting of high-risk individuals for lung cancer screening, however further research is urgently needed in this area.

### Supplementary information

Additional Supplementary Material

STROBE checklist for cohort studies_Janietal

## Data Availability

The data that support the findings of this study are available from SAIL and UK Biobank project site, subject to successful registration and application process.
