# Supplementary material for: Personalised lung cancer risk stratification and lung cancer screening: do general practice electronic medical records have a role?
Source: Br J Cancer. 2023 Oct 25;129(12):1968–77. doi: 10.1038/s41416-023-02467-9 (PMC10703821; doi:10.1038/s41416-023-02467-9)
Supplement: Supplementary file 1 — Additional Supplementary Material [file 41416_2023_2467_MOESM1_ESM.docx]

**Title: Additional Supplementary Material**

**Table S1 Read codes used to define smoking status**(1)**.**

| **Read codes** | **Description** | **Category** | **Notes** |
| --- | --- | --- | --- |
| 1371. | Never smoked tobacco | N |  |
| 9kn.. | Non-smoker annual review - enhanced services administration | N |  |
| 137K. | Stopped smoking | E |  |
| 137L. | Current non-smoker | E |  |
| 137N. | Ex pipe smoker | E |  |
| 137O. | Ex cigar smoker | E |  |
| 137S. | Ex smoker | E |  |
| 137T. | Date ceased smoking | E |  |
| 1377. | Ex-trivial smoker (< 1 per day) | E |  |
| 1378. | Ex-light smoker (1 - 9 per day) | E |  |
| 1379. | Ex-moderate smoker (10 - 19 per day) | E |  |
| 137A. | Ex-heavy smoker (20 - 39 per day) | E |  |
| 137B. | Ex-very heavy smoker (40 + per day) | E |  |
| 137F. | Ex-smoker - amount unknown | E |  |
| 137i. | Ex tobacco chewer | E |  |
| 137j. | Ex-cigarette smoker | E |  |
| 137K0 | Recently stopped smoking | E |  |
| 9km.. | Ex-smoker annual review - enhanced services administration | E |  |
| 13p4. | Smoking free weeks | E |  |
| 137l. | Ex roll-up cigarette smoker | E |  |
| 745H% | (Various) Smoking cessation therapy | S |  |
| du3% | (Various) Nicotine replacement therapy | S |  |
| du6% | (Various) Bupropion | S |  |
| du7% | (Various) additional nicotine replacement therapy | S |  |
| du8% | (Various) Varenicline | S |  |
| du9% | (Various) Nicotine withdrawal products | S |  |
| E251% | (Various) tobacco dependence | S |  |
| 137.. | Tobacco consumption | S | All with EVENT_VAL greater than 0 |
| 137Z | Tobacco consumption NOS | S |  |
| 137X. | Cigarette consumption | S |  |
| 137Y. | Cigar consumption | S |  |
| 137E. | Tobacco consumption unknown | S |  |
| 137g. | Cigarette pack years | S |  |
| 1372. | Trivial smoker - < 1 per day | S |  |
| 1373. | Light smoker - 1-9 per day | S |  |
| 1374. | Moderate smoker - 10-19 per day | S |  |
| 1375. | Heavy smoker - 20-39 per day | S |  |
| 1376. | Very heavy smoker - 20-39 per day | S |  |
| 137a. | Pipe tobacco consumption | S |  |
| 137b. | Ready to stop smoking | S |  |
| 137C. | Keeps trying to stop smoking | S |  |
| 137c. | Thinking about stopping smoking | S |  |
| 137e. | Smoking restarted | S |  |
| 137G. | Trying to give up smoking | S |  |
| 137H. | Pipe smoker | S |  |
| 137J. | Cigar smoker | S |  |
| 137M. | Rolls own cigarettes | S |  |
| 137P. | Cigarette smoker | S |  |
| 137Q. | Smoking started | S |  |
| 137R. | Current smoker | S |  |
| 137V. | Smoking reduced | S |  |
| 137D. | Admitted tobacco cons untrue ? | S |  |
| 137d. | Not interested in stopping smoking | S |  |
| 137f. | Reason for restarting smoking | S |  |
| 137h. | Minutes from waking to first tobacco consumption | S |  |
| 6791. | Health ed. - smoking | S |  |
| 67910 | Health education - parental smoking | S |  |
| 137m. | Failed attempt to stop smoking | S |  |
| 13p.. | Smoking cessation milestones | S |  |
| 13p0. | Negotiated date for cessation of smoking | S |  |
| 13p8. | Lost to smoking cessation follow-up | S |  |
| 38DH. | Fagerstrom test for nicotine dependence | S |  |
| 67A3. | Pregnancy smoking advice | S |  |
| 67H1. | Lifestyle advice regarding smoking | S |  |
| 67H6. | Brief cessation for smoking cessation | S |  |
| 8B2B. | Nicotine replacement therapy | S |  |
| 8B3f. | Nicotine replacement therapy provided free | S |  |
| 8B3Y. | Over the counter nicotine replacement therapy | S |  |
| 8BP3. | Nicotine replacement therapy provided by community pharmacis | S |  |
| 8CAg. | Smoking cessation advice provided by community pharmacist | S |  |
| 8CAL. | Smoking cessation advice | S |  |
| 8CdB. | Stop smoking service opportunity signposted | S |  |
| 8H7i. | Referral to smoking cessation advisor | S |  |
| 8HBM. | Stop smoking face to face follow-up | S |  |
| 8HkQ. | Referral to NHS stop smoking service | S |  |
| 8HTK. | Referral to stop-smoking clinic | S |  |
| 8I2I. | Nicotine replacement therapy contraindicated | S |  |
| 8I2J. | Bupropion contraindicated | S |  |
| 8I39. | Nicotine replacement therapy refused | S |  |
| 8I3M. | Bupropion refused | S |  |
| 8I6H. | Smoking review not indicated | S |  |
| 8IAj. | Smoking cessation advice declined | S |  |
| 8IEK. | Smoking cessation program declined | S |  |
| 8IEM. | Smoking cessation drug therapy declined | S |  |
| 9hG.. | Exception reporting: smoking quality indicators | S |  |
| 9hG0. | Excepted from smoking quality indicators: Patient unsuitable | S |  |
| 9hG1. | Excepted from smoking quality indicators: Informed dissent | S |  |
| 9kc.. | Smoking cessation - enhanced services administration | S |  |
| 9kc0. | Smoking cessatn monitor template complet - enhanc serv admin | S |  |
| 9ko.. | Current smoker annual review - enhanced service admin | S |  |
| 9N2k. | Seen by smoking cessation advisor | S |  |
| 9N4M. | DNA - did not attend smoking cessation clinic | S |  |
| 9Ndg. | Declined consent for follow-up by smoking cessation team | S |  |
| 9NdV. | Consent given follow-up after smoking cessation intervention | S |  |
| 9NdW. | Consent given for smoking cessation data sharing | S |  |
| 9NdY. | Declin cons follow-up evaluation after smoking cess interven | S |  |
| 9NdZ. | Declined consent for smoking cessation data sharing | S |  |
| 9NS02 | Referral for smoking cessation service offered | S |  |
| 9OO.. | Attends stop smoking monitor admin | S |  |
| 9OO1. | Attends stop smoking monitor | S |  |
| 9OO2. | Refuses stop smoking monitor | S |  |
| 9OO3. | Stop smoking monitor default | S |  |
| 9OO4. | Stop smoking monitor 1st lettr | S |  |
| 9OO5. | Stop smoking monitor 2nd lettr | S |  |
| 9OO6. | Stop smoking monitor 3rd lettr | S |  |
| 9OO7. | Stop smoking monitor verb.inv. | S |  |
| 9OO8. | Stop smoking monitor phone inv | S |  |
| 9OO9. | Stop smoking monitoring delete | S |  |
| 9OOA. | Stop smoking monitor check.done | S |  |
| 9OOB. | Stop smoking invitation short message service text message | S |  |
| 9OOB0 | Stop smoking invitation first SMS text message | S |  |
| 9OOB1 | Stop smoking invitation second SMS text message | S |  |
| 9OOB2 | Stop smoking invitation third SMS text message | S |  |
| 9OOZ. | Stop smoking monitor admin.NOS | S |  |
| E023. | Nicotine withdrawal | S |  |
| J0364 | Tobacco deposit on teeth | S |  |
| SMC.. | Toxic effect of tobacco and nicotine | S |  |
| TJHy2 | Adverse reaction to nicotine | S |  |
| U6099 | [X] Bupropion causing adverse effects in therapeutic use | S |  |
| ZV4K0 | [V] Tobacco use | S |  |
| ZV6D8 | [V] Tobacco abuse counselling | S |  |
| 13p5. | Smoking cessation programme start date | S |  |
| 9ko.. | Current smoker annual review - enhanced service admin | S |  |

Categories: S smoker, E ex-smoker, N never-smoker

Table S2 Median duration between the last known entry in the electronic medical record and the start of the follow-up period

|  | Non-smoker | Ex-smoker | Smoker |
| --- | --- | --- | --- |
| SAIL | Median: 459 days (IQR: 179-1073 days) | Median: 482 days (IQR: 187-1152 days) | Median: 514 days (IQR: 193-1243 days) |
| UK Biobank | Median: 538 days (IQR: 220-1081 days) | Median: 1448 days (IQR: 425-3990 days) | Median: 687 days (IQR: 280-2647 days) |

**Table S3 Read codes used for defining long term conditions prevalence**(2)**.**

| Read Code | Description |
| --- | --- |
| 662G. | Hypertensive treatm.changed |
| 662O. | On treatment for hypertension |
| 662P. | Hypertension monitoring |
| 662q. | Trial reduction of antihypertensive therapy |
| 662r. | Trial withdrawal of antihypertensive therapy |
| 8B26. | Antihypertensive therapy |
| 8BL0. | Patient on maximal tolerated antihypertensive therapy |
| 8CR4. | Hypertension clinical management plan |
| F4042 | Blind hypertensive eye |
| F4213 | Hypertensive retinopathy |
| F4504 | Ocular hypertension |
| G2... | Hypertensive disease |
| G20.. | Essential hypertension |
| G200. | Malignant essential hypertension |
| G201. | Benign essential hypertension |
| G202. | Systolic hypertension |
| G203. | Diastolic hypertension |
| G20z. | Essential hypertension NOS |
| G21.. | Hypertensive heart disease |
| G210. | Malignant hypertensive heart disease |
| G2100 | Malignant hypertensive heart disease without CCF |
| G2101 | Malignant hypertensive heart disease with CCF |
| G210z | Malignant hypertensive heart disease NOS |
| G211. | Benign hypertensive heart disease |
| G2110 | Benign hypertensive heart disease without CCF |
| G2111 | Benign hypertensive heart disease with CCF |
| G211z | Benign hypertensive heart disease NOS |
| G21z. | Hypertensive heart disease NOS |
| G21z0 | Hypertensive heart disease NOS without CCF |
| G21z1 | Hypertensive heart disease NOS with CCF |
| G21zz | Hypertensive heart disease NOS |
| G22.. | Hypertensive renal disease |
| G220. | Malignant hypertensive renal disease |
| G221. | Benign hypertensive renal disease |
| G222. | Hypertensive renal disease with renal failure |
| G22z. | Hypertensive renal disease NOS |
| G23.. | Hypertensive heart and renal disease |
| G230. | Malignant hypertensive heart and renal disease |
| G231. | Benign hypertensive heart and renal disease |
| G232. | Hypertensive heart&renal dis wth (congestive) heart failure |
| G233. | Hypertensive heart and renal disease with renal failure |
| G234. | Hyperten heart&renal dis+both(congestv)heart and renal fail |
| G23z. | Hypertensive heart and renal disease NOS |
| G24.. | Secondary hypertension |
| G240. | Secondary malignant hypertension |
| G2400 | Secondary malignant renovascular hypertension |
| G240z | Secondary malignant hypertension NOS |
| G241. | Secondary benign hypertension |
| G2410 | Secondary benign renovascular hypertension |
| G241z | Secondary benign hypertension NOS |
| G244. | Hypertension secondary to endocrine disorders |
| G24z. | Secondary hypertension NOS |
| G24z0 | Secondary renovascular hypertension NOS |
| G24z1 | Hypertension secondary to drug |
| G24zz | Secondary hypertension NOS |
| G2y.. | Other specified hypertensive disease |
| G2z.. | Hypertensive disease NOS |
| G672. | Hypertensive encephalopathy |
| Gyu2. | [X]Hypertensive diseases |
| Gyu20 | [X]Other secondary hypertension |
| Gyu21 | [X]Hypertension secondary to other renal disorders |
| 1B17. | Depressed |
| 1B1U. | Symptoms of depression |
| 1BT.. | Depressed mood |
| 2257 | O/E - depressed |
| 62T1. | Puerperal depression |
| 66590 | Antidepressant drug treatment started |
| 6G00. | Postnatal depression counselling |
| 8CAa. | Patient given advice about management of depression |
| 8HHq. | Referral for guided self-help for depression |
| 9H90. | Depression annual review |
| 9H91. | Depression medication review |
| 9H92. | Depression interim review |
| 9HA0. | On depression register |
| E0013 | Presenile dementia with depression |
| E002. | Senile dementia with depressive or paranoid features |
| E0021 | Senile dementia with depression |
| E002z | Senile dementia with depressive or paranoid features NOS |
| E0043 | Arteriosclerotic dementia with depression |
| E02y3 | Drug-induced depressive state |
| E112. | Single major depressive episode |
| E1120 | Single major depressive episode, unspecified |
| E1121 | Single major depressive episode, mild |
| E1122 | Single major depressive episode, moderate |
| E1123 | Single major depressive episode, severe, without psychosis |
| E1124 | Single major depressive episode, severe, with psychosis |
| E1125 | Single major depressive episode, partial or unspec remission |
| E1126 | Single major depressive episode, in full remission |
| E112z | Single major depressive episode NOS |
| E113. | Recurrent major depressive episode |
| E1130 | Recurrent major depressive episodes, unspecified |
| E1131 | Recurrent major depressive episodes, mild |
| E1132 | Recurrent major depressive episodes, moderate |
| E1133 | Recurrent major depressive episodes, severe, no psychosis |
| E1134 | Recurrent major depressive episodes, severe, with psychosis |
| E1135 | Recurrent major depressive episodes,partial/unspec remission |
| E1136 | Recurrent major depressive episodes, in full remission |
| E1137 | Recurrent depression |
| E113z | Recurrent major depressive episode NOS |
| E118. | Seasonal affective disorder |
| E11y2 | Atypical depressive disorder |
| E11z2 | Masked depression |
| E130. | Reactive depressive psychosis |
| E135. | Agitated depression |
| E204. | Neurotic depression reactive type |
| E2003 | Anxiety with depression |
| E290. | Brief depressive reaction |
| E290z | Brief depressive reaction NOS |
| E291. | Prolonged depressive reaction |
| E2B.. | Depressive disorder NEC |
| E2B0. | Postviral depression |
| E2B1. | Chronic depression |
| Eu204 | [X]Post-schizophrenic depression |
| Eu251 | [X]Schizoaffective disorder, depressive type |
| Eu32. | [X]Depressive episode |
| Eu320 | [X]Mild depressive episode |
| Eu321 | [X]Moderate depressive episode |
| Eu322 | [X]Severe depressive episode without psychotic symptoms |
| Eu323 | [X]Severe depressive episode with psychotic symptoms |
| Eu324 | [X]Mild depression |
| Eu325 | [X]Major depression, mild |
| Eu326 | [X]Major depression, moderately severe |
| Eu327 | [X]Major depression, severe without psychotic symptoms |
| Eu328 | [X]Major depression, severe with psychotic symptoms |
| Eu329 | [X]Single major depr ep, severe with psych, psych in remiss |
| Eu32A | [X]Recurr major depr ep, severe with psych, psych in remiss |
| Eu32y | [X]Other depressive episodes |
| Eu32z | [X]Depressive episode, unspecified |
| Eu33. | [X]Recurrent depressive disorder |
| Eu330 | [X]Recurrent depressive disorder, current episode mild |
| Eu331 | [X]Recurrent depressive disorder, current episode moderate |
| Eu332 | [X]Recurr depress disorder cur epi severe without psyc sympt |
| Eu333 | [X]Recurrent depress disorder cur epi severe with psyc symp |
| Eu334 | [X]Recurrent depressive disorder, currently in remission |
| Eu33y | [X]Other recurrent depressive disorders |
| Eu33z | [X]Recurrent depressive disorder, unspecified |
| Eu341 | [X]Dysthymia |
| Eu412 | [X]Mixed anxiety and depressive disorder |
| Eu920 | [X]Depressive conduct disorder |
| ZN120 | EPDS score 12 to 13 |
| ZN121 | EPDS score above 13 |
| ZN123 | EPDS 8 month score 1-11 |
| ZN124 | EPDS 8 month score 12-13 |
| ZN125 | EPDS 8 month score above 13 |
| 173A. | Exercise induced asthma |
| 173c. | Occupational asthma |
| 173d. | Work aggravated asthma |
| 1780 | Aspirin induced asthma |
| 1O2.. | Asthma confirmed |
| 663d. | Emergency asthma admission since last appointment |
| 663e. | Asthma restricts exercise |
| 6.63E+02 | Asthma sometimes restricts exercise |
| 6.63E+03 | Asthma severely restricts exercise |
| 663f. | Asthma never restricts exercise |
| 663h. | Asthma - currently dormant |
| 663j. | Asthma - currently active |
| 663m. | Asthma accident and emergency attendance since last visit |
| 663n. | Asthma treatment compliance satisfactory |
| 663N. | Asthma disturbing sleep |
| 663N0 | Asthma causing night waking |
| 663N1 | Asthma disturbs sleep weekly |
| 663N2 | Asthma disturbs sleep frequently |
| 663O. | Asthma not disturbing sleep |
| 663O0 | Asthma never disturbs sleep |
| 663p. | Asthma treatment compliance unsatisfactory |
| 663P. | Asthma limiting activities |
| 663q. | Asthma daytime symptoms |
| 663Q. | Asthma not limiting activities |
| 663r. | Asthma causes night symptoms 1 to 2 times per month |
| 663s. | Asthma never causes daytime symptoms |
| 663t. | Asthma causes daytime symptoms 1 to 2 times per month |
| 663u. | Asthma causes daytime symptoms 1 to 2 times per week |
| 663U. | Asthma management plan given |
| 663v. | Asthma causes daytime symptoms most days |
| 663V. | Asthma severity |
| 663P. | Asthma limiting activities |
| 663q. | Asthma daytime symptoms |
| 663r. | Asthma causes night symptoms 1 to 2 times per month |
| 663u. | Asthma causes daytime symptoms 1 to 2 times per week |
| 663v. | Asthma causes daytime symptoms most days |
| 663V0 | Occasional asthma |
| 663V1 | Mild asthma |
| 663V2 | Moderate asthma |
| 663V3 | Severe asthma |
| 663w. | Asthma limits walking up hills or stairs |
| 663W. | Asthma prophylactic medication used |
| 663x. | Asthma limits walking on the flat |
| 663y. | Number of asthma exacerbations in past year |
| 66Y5. | Change in asthma management plan |
| 66Y9. | Step up change in asthma management plan |
| 66YA. | Step down change in asthma management plan |
| 66YC. | Absent from work or school due to asthma |
| 66YE. | Asthma monitoring due |
| 66YJ. | Asthma annual review |
| 66YK. | Asthma follow-up |
| 66YP. | Asthma night-time symptoms |
| 66YQ. | Asthma monitoring by nurse |
| 66YR. | Asthma monitoring by doctor |
| 8793 | Asthma control step 0 |
| 8794 | Asthma control step 1 |
| 8795 | Asthma control step 2 |
| 8796 | Asthma control step 3 |
| 8797 | Asthma control step 4 |
| 8798 | Asthma control step 5 |
| 8B3j. | Asthma medication review |
| 8CR0. | Asthma clinical management plan |
| 8791 | Further asthma - drug prevent. |
| 9hA.. | Exception reporting: asthma quality indicators |
| H3120 | Chronic asthmatic bronchitis |
| H33.. | Asthma |
| H330. | Extrinsic (atopic) asthma |
| H3300 | Extrinsic asthma without status asthmaticus |
| H3301 | Extrinsic asthma with status asthmaticus |
| H330z | Extrinsic asthma NOS |
| H331. | Intrinsic asthma |
| H3310 | Intrinsic asthma without status asthmaticus |
| H3311 | Intrinsic asthma with status asthmaticus |
| H331z | Intrinsic asthma NOS |
| H332. | Mixed asthma |
| H333. | Acute exacerbation of asthma |
| H334. | Brittle asthma |
| H33z. | Asthma unspecified |
| H33z0 | Status asthmaticus NOS |
| H33z1 | Asthma attack |
| H33z2 | Late-onset asthma |
| H33zz | Asthma NOS |
| H35y6 | Sequoiosis (red-cedar asthma) |
| H35y7 | Wood asthma |
| H47y0 | Detergent asthma |
| 14AL. | H/O: Treatment for ischaemic heart disease |
| G3... | Ischaemic heart disease |
| G30B. | Acute posterolateral myocardial infarction |
| G31.. | Other acute and subacute ischaemic heart disease |
| G310. | Postmyocardial infarction syndrome |
| G3110 | Myocardial infarction aborted |
| G312. | Coronary thrombosis not resulting in myocardial infarction |
| G31y. | Other acute and subacute ischaemic heart disease |
| G31y0 | Acute coronary insufficiency |
| G31y1 | Microinfarction of heart |
| G31y2 | Subendocardial ischaemia |
| G31y3 | Transient myocardial ischaemia |
| G31yz | Other acute and subacute ischaemic heart disease NOS |
| G32.. | Old myocardial infarction |
| G33z. | Angina pectoris NOS |
| G33z0 | Status anginosus |
| G33z1 | Stenocardia |
| G33z2 | Syncope anginosa |
| G33z3 | Angina on effort |
| G33z4 | Ischaemic chest pain |
| G33z5 | Post infarct angina |
| G33z6 | New onset angina |
| G33z7 | Stable angina |
| G33zz | Angina pectoris NOS |
| G34.. | Other chronic ischaemic heart disease |
| G340. | Coronary atherosclerosis |
| G3400 | Single coronary vessel disease |
| G3401 | Double coronary vessel disease |
| G34.. | Other chronic ischaemic heart disease |
| G340. | Coronary atherosclerosis |
| G3400 | Single coronary vessel disease |
| G3401 | Double coronary vessel disease |
| G3412 | Aneurysm of coronary vessels |
| G342. | Atherosclerotic cardiovascular disease |
| G343. | Ischaemic cardiomyopathy |
| G344. | Silent myocardial ischaemia |
| G34y. | Other specified chronic ischaemic heart disease |
| G34y0 | Chronic coronary insufficiency |
| G34y1 | Chronic myocardial ischaemia |
| G34yz | Other specified chronic ischaemic heart disease NOS |
| G34z. | Other chronic ischaemic heart disease NOS |
| G34z0 | Asymptomatic coronary heart disease |
| G35.. | Subsequent myocardial infarction |
| G350. | Subsequent myocardial infarction of anterior wall |
| G351. | Subsequent myocardial infarction of inferior wall |
| G353. | Subsequent myocardial infarction of other sites |
| G35X. | Subsequent myocardial infarction of unspecified site |
| G36.. | Certain current complication follow acute myocardial infarct |
| G360. | Haemopericardium/current comp folow acut myocard infarct |
| G361. | Atrial septal defect/curr comp folow acut myocardal infarct |
| G362. | Ventric septal defect/curr comp fol acut myocardal infarctn |
| G363. | Ruptur cardiac wall w'out haemopericard/cur comp fol ac MI |
| G364. | Ruptur chordae tendinae/curr comp fol acute myocard infarct |
| G365. | Rupture papillary muscle/curr comp fol acute myocard infarct |
| G366. | Thrombosis atrium,auric append&vent/curr comp foll acute MI |
| G38.. | Postoperative myocardial infarction |
| G380. | Postoperative transmural myocardial infarction anterior wall |
| G381. | Postoperative transmural myocardial infarction inferior wall |
| G382. | Postoperative transmural myocardial infarction other sites |
| G383. | Postoperative transmural myocardial infarction unspec site |
| G384. | Postoperative subendocardial myocardial infarction |
| G38z. | Postoperative myocardial infarction, unspecified |
| G3y.. | Other specified ischaemic heart disease |
| G3z.. | Ischaemic heart disease NOS |
| G501. | Post infarction pericarditis |
| G5y2. | Cardiovascular arteriosclerosis unspecified |
| Gyu3. | [X]Ischaemic heart diseases |
| Gyu31 | [X]Other current complicatns following acute myocard infarct |
| Gyu32 | [X]Other forms of acute ischaemic heart disease |
| Gyu33 | [X]Other forms of chronic ischaemic heart disease |
| Gyu35 | [X]Subsequent myocardial infarction of other sites |
| Gyu36 | [X]Subsequent myocardial infarction of unspecified site |
| 13AB. | Diabetic lipid lowering diet |
| 13AC. | Diabetic weight reducing diet |
| 13B1. | Diabetic diet |
| 1434 | H/O: diabetes mellitus |
| 14F4. | H/O: Admission in last year for diabetes foot problem |
| 2BBF. | Retinal abnormality - diabetes related |
| 2BBk. | O/E - right eye stable treated prolif diabetic retinopathy |
| 2BBL. | O/E - diabetic maculopathy present both eyes |
| 2BBl. | O/E - left eye stable treated prolif diabetic retinopathy |
| 2BBo. | O/E - sight threatening diabetic retinopathy |
| 2BBP. | O/E - right eye background diabetic retinopathy |
| 2BBQ. | O/E - left eye background diabetic retinopathy |
| 2BBR. | O/E - right eye preproliferative diabetic retinopathy |
| 2BBr. | Impaired vision due to diabetic retinopathy |
| 2BBS. | O/E - left eye preproliferative diabetic retinopathy |
| 2BBT. | O/E - right eye proliferative diabetic retinopathy |
| 2BBV. | O/E - left eye proliferative diabetic retinopathy |
| 2BBW. | O/E - right eye diabetic maculopathy |
| 2BBX. | O/E - left eye diabetic maculopathy |
| 2G510 | Foot abnormality - diabetes related |
| 2G5A. | O/E - Right diabetic foot at risk |
| 2G5B. | O/E - Left diabetic foot at risk |
| 2G5C. | Foot abnormality - diabetes related |
| 2G5E. | O/E - Right diabetic foot at low risk |
| 2G5F. | O/E - Right diabetic foot at moderate risk |
| 2G5G. | O/E - Right diabetic foot at high risk |
| 2G5H. | O/E - Right diabetic foot - ulcerated |
| 2G5I. | O/E - Left diabetic foot at low risk |
| 2G5J. | O/E - Left diabetic foot at moderate risk |
| 2G5K. | O/E - Left diabetic foot at high risk |
| 2G5L. | O/E - Left diabetic foot - ulcerated |
| 2G5V. | O/E - right chronic diabetic foot ulcer |
| 2G5W. | O/E - left chronic diabetic foot ulcer |
| 3881 | Education score - diabetes |
| 3882 | Diabetes well being questionnaire |
| 3883 | Diabetes treatment satisfaction questionnaire |
| 42c.. | HbA1 - diabetic control |
| 42c0. | HbA1 < 7% - good control |
| 42c1. | HbA1 7 - 10% - borderline control |
| 42c2. | HbA1 > 10% - bad control |
| 42W.. | Hb. A1C - diabetic control |
| 42W1. | Hb. A1C < 7% - good control |
| 42W2. | Hb. A1C 7-10% - borderline |
| 42W3. | Hb. A1C > 10% - bad control |
| 42WZ. | Hb. A1C - diabetic control NOS |
| 44V3. | Glucose tol. test diabetic |
| 66A.. | Diabetic monitoring |
| 66A1. | Initial diabetic assessment |
| 66A2. | Follow-up diabetic assessment |
| 66A3. | Diabetic on diet only |
| 66A4. | Diabetic on oral treatment |
| 66A5. | Diabetic on insulin |
| 66A6. | Last hypo. attack |
| 66A7. | Frequency of hypo. attacks |
| 66A70 | Frequency of hospital treated hypoglycaemia |
| 66A71 | Frequency of GP or paramedic treated hypoglycaemia |
| 66A8. | Has seen dietician - diabetes |
| 66A9. | Understands diet - diabetes |
| 66Aa. | Diabetic diet - poor compliance |
| 66AA. | Injection sites |
| 66Ab. | Diabetic foot examination |
| 66AB. | Urine sugar charts |
| 66Ac. | Diabetic peripheral neuropathy screening |
| 66AC. | Blood sugar charts |
| 66Ad. | Hypoglycaemic attack requiring 3rd party assistance |
| 66AD. | Fundoscopy - diabetic check |
| 66Ae. | HBA1c target |
| 66Af. | Patient diabetes education review |
| 66Ag. | Insulin needles changed daily |
| 66Ah. | Insulin needles changed for each injection |
| 66AH. | Diabetic treatment changed |
| 66AH0 | Conversion to insulin |
| 66Ai. | Diabetic 6 month review |
| 66AI. | Diabetic - good control |
| 66Aj. | Insulin needles changed less than once a day |
| 66AJ. | Diabetic - poor control |
| 66AJ0 | Chronic hyperglycaemia |
| 66AJ1 | Brittle diabetes |
| 66AJ2 | Loss of hypoglycaemic warning |
| 66AJ3 | Recurrent severe hypos |
| 66AJz | Diabetic - poor control NOS |
| 66Ak. | Diabetic monitoring - lower risk albumin excretion |
| 66AK. | Diabetic - cooperative patient |
| 66Al. | Diabetic monitoring - higher risk albumin excretion |
| 66AL. | Diabetic-uncooperative patient |
| 66Am. | Insulin dose changed |
| 66AM. | Diabetic - follow-up default |
| 66An. | Diabetes type 1 review |
| 66AN. | Date diabetic treatment start |
| 66Ao. | Diabetes type 2 review |
| 66Ap. | Insulin treatment initiated |
| 66AP. | Diabetes: practice programme |
| 66Aq. | Diabetic foot screen |
| 66AQ. | Diabetes: shared care programme |
| 66AR. | Diabetes management plan given |
| 66AS. | Diabetic annual review |
| 66AT. | Annual diabetic blood test |
| 66AU. | Diabetes care by hospital only |
| 66AV. | Diabetic on insulin and oral treatment |
| 66AW. | Diabetic foot risk assessment |
| 66AX. | Diabetes: shared care in pregnancy - diabetol and obstet |
| 66AY. | Diabetic diet - good compliance |
| 66AZ. | Diabetic monitoring NOS |
| 6761 | Diabetic pre-pregnancy counselling |
| 7L198 | Subcutaneous injection of insulin |
| 8A12. | Diabetic crisis monitoring |
| 8A13. | Diabetic stabilisation |
| 8A17. | Self monitoring of blood glucose |
| 8A19. | Self monitoring of blood and urine glucose |
| 8B3l. | Diabetes medication review |
| 8CA41 | Pt advised re diabetic diet |
| 8CP2. | Transition of diabetes care options discussed |
| 8CR2. | Diabetes clinical management plan |
| 8CS0. | Diabetes care plan agreed |
| C10.. | Diabetes mellitus |
| C100. | Diabetes mellitus with no mention of complication |
| C1000 | Diabetes mellitus, juvenile type, no mention of complication |
| C1001 | Diabetes mellitus, adult onset, no mention of complication |
| C100z | Diabetes mellitus NOS with no mention of complication |
| C101. | Diabetes mellitus with ketoacidosis |
| C1010 | Diabetes mellitus, juvenile type, with ketoacidosis |
| C1011 | Diabetes mellitus, adult onset, with ketoacidosis |
| C101y | Other specified diabetes mellitus with ketoacidosis |
| C101z | Diabetes mellitus NOS with ketoacidosis |
| C102. | Diabetes mellitus with hyperosmolar coma |
| C1020 | Diabetes mellitus, juvenile type, with hyperosmolar coma |
| C1021 | Diabetes mellitus, adult onset, with hyperosmolar coma |
| C102z | Diabetes mellitus NOS with hyperosmolar coma |
| C103. | Diabetes mellitus with ketoacidotic coma |
| C1030 | Diabetes mellitus, juvenile type, with ketoacidotic coma |
| C1031 | Diabetes mellitus, adult onset, with ketoacidotic coma |
| C103y | Other specified diabetes mellitus with coma |
| C103z | Diabetes mellitus NOS with ketoacidotic coma |
| C104. | Diabetes mellitus with renal manifestation |
| C1040 | Diabetes mellitus, juvenile type, with renal manifestation |
| C1041 | Diabetes mellitus, adult onset, with renal manifestation |
| C104y | Other specified diabetes mellitus with renal complications |
| C104z | Diabetes mellitus with nephropathy NOS |
| C105. | Diabetes mellitus with ophthalmic manifestation |
| C1050 | Diabetes mellitus, juvenile type, + ophthalmic manifestation |
| C1051 | Diabetes mellitus, adult onset, + ophthalmic manifestation |
| C105y | Other specified diabetes mellitus with ophthalmic complicatn |
| C105z | Diabetes mellitus NOS with ophthalmic manifestation |
| C106. | Diabetes mellitus with neurological manifestation |
| C1060 | Diabetes mellitus, juvenile, + neurological manifestation |
| C1061 | Diabetes mellitus, adult onset, + neurological manifestation |
| C106y | Other specified diabetes mellitus with neurological comps |
| C106z | Diabetes mellitus NOS with neurological manifestation |
| C107. | Diabetes mellitus with peripheral circulatory disorder |
| C1070 | Diabetes mellitus, juvenile +peripheral circulatory disorder |
| C1071 | Diabetes mellitus, adult, + peripheral circulatory disorder |
| C1072 | Diabetes mellitus, adult with gangrene |
| C1073 | IDDM with peripheral circulatory disorder |
| C1074 | NIDDM with peripheral circulatory disorder |
| C107y | Other specified diabetes mellitus with periph circ comps |
| C107z | Diabetes mellitus NOS with peripheral circulatory disorder |
| C108. | Insulin dependent diabetes mellitus |
| C1080 | Insulin-dependent diabetes mellitus with renal complications |
| C1081 | Insulin-dependent diabetes mellitus with ophthalmic comps |
| C1082 | Insulin-dependent diabetes mellitus with neurological comps |
| C1083 | Insulin dependent diabetes mellitus with multiple complicatn |
| C1084 | Unstable insulin dependent diabetes mellitus |
| C1085 | Insulin dependent diabetes mellitus with ulcer |
| C1086 | Insulin dependent diabetes mellitus with gangrene |
| C1087 | Insulin dependent diabetes mellitus with retinopathy |
| C1088 | Insulin dependent diabetes mellitus - poor control |
| C1089 | Insulin dependent diabetes maturity onset |
| C108A | Insulin-dependent diabetes without complication |
| C108B | Insulin dependent diabetes mellitus with mononeuropathy |
| C108C | Insulin dependent diabetes mellitus with polyneuropathy |
| C108D | Insulin dependent diabetes mellitus with nephropathy |
| C108E | Insulin dependent diabetes mellitus with hypoglycaemic coma |
| C108F | Insulin dependent diabetes mellitus with diabetic cataract |
| C108G | Insulin dependent diab mell with peripheral angiopathy |
| C108H | Insulin dependent diabetes mellitus with arthropathy |
| C108J | Insulin dependent diab mell with neuropathic arthropathy |
| C108y | Other specified diabetes mellitus with multiple comps |
| C108z | Unspecified diabetes mellitus with multiple complications |
| C109. | Non-insulin dependent diabetes mellitus |
| C1090 | Non-insulin-dependent diabetes mellitus with renal comps |
| C1091 | Non-insulin-dependent diabetes mellitus with ophthalm comps |
| C1092 | Non-insulin-dependent diabetes mellitus with neuro comps |
| C1093 | Non-insulin-dependent diabetes mellitus with multiple comps |
| C1094 | Non-insulin dependent diabetes mellitus with ulcer |
| C1095 | Non-insulin dependent diabetes mellitus with gangrene |
| C1096 | Non-insulin-dependent diabetes mellitus with retinopathy |
| C1097 | Non-insulin dependent diabetes mellitus - poor control |
| C1099 | Non-insulin-dependent diabetes mellitus without complication |
| C109A | Non-insulin dependent diabetes mellitus with mononeuropathy |
| C109B | Non-insulin dependent diabetes mellitus with polyneuropathy |
| C109C | Non-insulin dependent diabetes mellitus with nephropathy |
| C109D | Non-insulin dependent diabetes mellitus with hypoglyca coma |
| C109E | Non-insulin depend diabetes mellitus with diabetic cataract |
| C109F | Non-insulin-dependent d m with peripheral angiopath |
| C109G | Non-insulin dependent diabetes mellitus with arthropathy |
| C109H | Non-insulin dependent d m with neuropathic arthropathy |
| C109J | Insulin treated Type 2 diabetes mellitus |
| C109K | Hyperosmolar non-ketotic state in type 2 diabetes mellitus |
| C10A. | Malnutrition-related diabetes mellitus |
| C10A0 | Malnutrition-related diabetes mellitus with coma |
| C10A1 | Malnutrition-related diabetes mellitus with ketoacidosis |
| C10A2 | Malnutrition-related diabetes mellitus with renal complicatn |
| C10A3 | Malnutrit-related diabetes mellitus wth ophthalmic complicat |
| C10A4 | Malnutrition-related diabetes mellitus wth neuro complicatns |
| C10A5 | Malnutritn-relat diabetes melitus wth periph circul complctn |
| C10A6 | Malnutrition-related diabetes mellitus with multiple comps |
| C10A7 | Malnutrition-related diabetes mellitus without complications |
| C10AW | Malnutrit-related diabetes mellitus with unspec complics |
| C10AX | Malnutrit-relat diabetes mellitus with other spec comps |
| C10B. | Diabetes mellitus induced by steroids |
| C10B0 | Steroid induced diabetes mellitus without complication |
| C10C. | Diabetes mellitus autosomal dominant |
| C10D. | Diabetes mellitus autosomal dominant type 2 |
| C10E. | Type 1 diabetes mellitus |
| C10E0 | Type 1 diabetes mellitus with renal complications |
| C10E1 | Type 1 diabetes mellitus with ophthalmic complications |
| C10E2 | Type 1 diabetes mellitus with neurological complications |
| C10E3 | Type 1 diabetes mellitus with multiple complications |
| C10E4 | Unstable type 1 diabetes mellitus |
| C10E5 | Type 1 diabetes mellitus with ulcer |
| C10E6 | Type 1 diabetes mellitus with gangrene |
| C10E7 | Type 1 diabetes mellitus with retinopathy |
| C10E8 | Type 1 diabetes mellitus - poor control |
| C10E9 | Type 1 diabetes mellitus maturity onset |
| C10EA | Type 1 diabetes mellitus without complication |
| C10EB | Type 1 diabetes mellitus with mononeuropathy |
| C10EC | Type 1 diabetes mellitus with polyneuropathy |
| C10ED | Type 1 diabetes mellitus with nephropathy |
| C10EE | Type 1 diabetes mellitus with hypoglycaemic coma |
| C10EF | Type 1 diabetes mellitus with diabetic cataract |
| C10EG | Type 1 diabetes mellitus with peripheral angiopathy |
| C10EH | Type 1 diabetes mellitus with arthropathy |
| C10EJ | Type 1 diabetes mellitus with neuropathic arthropathy |
| C10EK | Type 1 diabetes mellitus with persistent proteinuria |
| C10EL | Type 1 diabetes mellitus with persistent microalbuminuria |
| C10EM | Type 1 diabetes mellitus with ketoacidosis |
| C10EN | Type 1 diabetes mellitus with ketoacidotic coma |
| C10EP | Type 1 diabetes mellitus with exudative maculopathy |
| C10EQ | Type 1 diabetes mellitus with gastroparesis |
| C10ER | Latent autoimmune diabetes mellitus in adult |
| C10F. | Type 2 diabetes mellitus |
| C10F0 | Type 2 diabetes mellitus with renal complications |
| C10F1 | Type 2 diabetes mellitus with ophthalmic complications |
| C10F2 | Type 2 diabetes mellitus with neurological complications |
| C10F3 | Type 2 diabetes mellitus with multiple complications |
| C10F4 | Type 2 diabetes mellitus with ulcer |
| C10F5 | Type 2 diabetes mellitus with gangrene |
| C10F6 | Type 2 diabetes mellitus with retinopathy |
| C10F7 | Type 2 diabetes mellitus - poor control |
| C10F9 | Type 2 diabetes mellitus without complication |
| C10FA | Type 2 diabetes mellitus with mononeuropathy |
| C10FB | Type 2 diabetes mellitus with polyneuropathy |
| C10FC | Type 2 diabetes mellitus with nephropathy |
| C10FD | Type 2 diabetes mellitus with hypoglycaemic coma |
| C10FE | Type 2 diabetes mellitus with diabetic cataract |
| C10FF | Type 2 diabetes mellitus with peripheral angiopathy |
| C10FG | Type 2 diabetes mellitus with arthropathy |
| C10FH | Type 2 diabetes mellitus with neuropathic arthropathy |
| C10FJ | Insulin treated Type 2 diabetes mellitus |
| C10FK | Hyperosmolar non-ketotic state in type 2 diabetes mellitus |
| C10FL | Type 2 diabetes mellitus with persistent proteinuria |
| C10FM | Type 2 diabetes mellitus with persistent microalbuminuria |
| C10FN | Type 2 diabetes mellitus with ketoacidosis |
| C10FP | Type 2 diabetes mellitus with ketoacidotic coma |
| C10FQ | Type 2 diabetes mellitus with exudative maculopathy |
| C10FR | Type 2 diabetes mellitus with gastroparesis |
| C10FS | Maternally inherited diabetes mellitus |
| C10G. | Secondary pancreatic diabetes mellitus |
| C10G0 | Secondary pancreatic diabetes mellitus without complication |
| C10H. | Diabetes mellitus induced by non-steroid drugs |
| C10H0 | DM induced by non-steroid drugs without complication |
| C10M. | Lipoatrophic diabetes mellitus |
| C10M0 | Lipoatrophic diabetes mellitus without complication |
| C10N. | Secondary diabetes mellitus |
| C10N0 | Secondary diabetes mellitus without complication |
| C10N1 | Cystic fibrosis related diabetes mellitus |
| C10y. | Diabetes mellitus with other specified manifestation |
| C10y0 | Diabetes mellitus, juvenile, + other specified manifestation |
| C10y1 | Diabetes mellitus, adult, + other specified manifestation |
| C10yy | Other specified diabetes mellitus with other spec comps |
| C10yz | Diabetes mellitus NOS with other specified manifestation |
| C10z. | Diabetes mellitus with unspecified complication |
| C10z0 | Diabetes mellitus, juvenile type, + unspecified complication |
| C10z1 | Diabetes mellitus, adult onset, + unspecified complication |
| C10zy | Other specified diabetes mellitus with unspecified comps |
| C10zz | Diabetes mellitus NOS with unspecified complication |
| C11y0 | Steroid induced diabetes |
| Cyu2. | [X]Diabetes mellitus |
| Cyu20 | [X]Other specified diabetes mellitus |
| Cyu21 | [X]Malnutrit-relat diabetes mellitus with other spec comps |
| Cyu22 | [X]Malnutrit-related diabetes mellitus with unspec complics |
| Cyu23 | [X]Unspecified diabetes mellitus with renal complications |
| F1711 | Autonomic neuropathy due to diabetes |
| F3450 | Diabetic mononeuritis multiplex |
| F35z0 | Diabetic mononeuritis NOS |
| F372. | Polyneuropathy in diabetes |
| F3720 | Acute painful diabetic neuropathy |
| F3721 | Chronic painful diabetic neuropathy |
| F3722 | Asymptomatic diabetic neuropathy |
| F3813 | Myasthenic syndrome due to diabetic amyotrophy |
| F3y0. | Diabetic mononeuropathy |
| F420. | Diabetic retinopathy |
| F4200 | Background diabetic retinopathy |
| F4201 | Proliferative diabetic retinopathy |
| F4202 | Preproliferative diabetic retinopathy |
| F4203 | Advanced diabetic maculopathy |
| F4204 | Diabetic maculopathy |
| F4205 | Advanced diabetic retinal disease |
| F4206 | Non proliferative diabetic retinopathy |
| F4207 | High risk proliferative diabetic retinopathy |
| F4208 | High risk non proliferative diabetic retinopathy |
| F420z | Diabetic retinopathy NOS |
| F4407 | Diabetic iritis |
| F4640 | Diabetic cataract |
| G73y0 | Diabetic peripheral angiopathy |
| K01x1 | Nephrotic syndrome in diabetes mellitus |
| L1805 | Pre-existing diabetes mellitus, insulin-dependent |
| L1806 | Pre-existing diabetes mellitus, non-insulin-dependent |
| L1807 | Pre-existing malnutrition-related diabetes mellitus |
| L180X | Pre-existing diabetes mellitus, unspecified |
| Lyu29 | [X]Pre-existing diabetes mellitus, unspecified |
| M0372 | Cellulitis in diabetic foot |
| M2710 | Ischaemic ulcer diabetic foot |
| M2711 | Neuropathic diabetic ulcer - foot |
| M2712 | Mixed diabetic ulcer - foot |
| N0300 | Diabetic cheiroarthropathy |
| N0301 | Diabetic Charcot arthropathy |
| Q441. | Neonatal diabetes mellitus |
| R0542 | [D]Gangrene of toe in diabetic |
| R0543 | [D]Widespread diabetic foot gangrene |
| 22H2. | O/E - thyroid swelling -unilat |
| 22H3. | O/E - thyroid swelling -bilat. |
| 22H4. | O/E - thyroid lump |
| 66B.. | Thyroid disease monitoring |
| 66B1. | Initial thyroid assessment |
| 66B2. | Follow-up thyroid assessment |
| 66B3. | Inactive thyroid disease |
| 66B4. | Thyroid eye disease |
| 66B5. | Thyroid symptom change |
| 66B7. | Thyroid-dubious diagn.criteria |
| 66B8. | Thyroid dis.treatment changed |
| 66B9. | Thyroid dis.treatment started |
| 66BB. | Hypothyroidism annual review |
| 66BZ. | Thyroid disease monitoring NOS |
| 8CR5. | Hypothyroidism clinical management plan |
| C0... | Disorders of thyroid gland |
| C00.. | Simple and unspecified goitre |
| C000. | Simple goitre |
| C00z. | Goitre NOS |
| C01.. | Nontoxic nodular goitre |
| C010. | Nontoxic uninodular goitre |
| C011. | Nontoxic multinodular goitre |
| C01z. | Nontoxic nodular goitre NOS |
| C03.. | Congenital hypothyroidism |
| C03y. | Other specified congenital hypothyroidism |
| C03y0 | Congenital hypothyroidism with diffuse goitre |
| C03y1 | Congenital hypothyroidism without goitre |
| C03z. | Congenital hypothyroidism NOS |
| C04.. | Acquired hypothyroidism |
| C040. | Postsurgical hypothyroidism |
| C041. | Other postablative hypothyroidism |
| C0410 | Irradiation hypothyroidism |
| C041z | Postablative hypothyroidism NOS |
| C042. | Iodine hypothyroidism |
| C043. | Other iatrogenic hypothyroidism |
| C0430 | Hypothyroidism resulting from para-aminosalicylic acid |
| C0431 | Hypothyroidism resulting from phenylbutazone |
| C0432 | Hypothyroidism resulting from resorcinol |
| C043z | Iatrogenic hypothyroidism NOS |
| C044. | Postinfectious hypothyroidism |
| C047. | Subclinical hypothyroidism |
| C04y. | Other acquired hypothyroidism |
| C04z. | Hypothyroidism NOS |
| C04z0 | Premature puberty due to hypothyroidism |
| C04z1 | Myxoedema coma |
| C05.. | Thyroiditis |
| C050. | Acute thyroiditis |
| C0500 | Acute nonsuppurative thyroiditis |
| C0501 | Acute suppurative thyroiditis |
| C0502 | Abscess of thyroid |
| C050z | Acute thyroiditis NOS |
| C051. | Subacute thyroiditis |
| C052. | Chronic lymphocytic thyroiditis |
| C053. | Chronic fibrous thyroiditis |
| C054. | Iatrogenic thyroiditis |
| C05y. | Other and unspecified chronic thyroiditis |
| C05y4 | Chronic thyroiditis with transient thyrotoxicosis |
| C05z. | Thyroiditis NOS |
| C06.. | Other disorders of thyroid |
| C060. | Disorder of thyrocalcitonin secretion |
| C061. | Dyshormonogenic goitre |
| C062. | Thyroid cyst |
| C063. | Thyroid haemorrhage and infarction |
| C0630 | Thyroid haemorrhage |
| C0631 | Thyroid infarction |
| C063z | Thyroid haemorrhage or infarction NOS |
| C06y. | Other specified thyroid disorders |
| C06y0 | Thyroid-binding globulin abnormality |
| C06y1 | Thyroid atrophy |
| C06yz | Other specified thyroid disorder NOS |
| C06z. | Thyroid disorder NOS |
| C0A.. | Congenital iodine deficiency syndrome |
| C0A0. | Congenital iodine-deficiency syndrome, neurological type |
| C0A1. | Congenital iodine-deficiency syndrome, myxoedematous type |
| C0A2. | Congenital iodine-deficiency syndrome, mixed type |
| C0A3. | Iodine-deficiency-related diffuse (endemic) goitre |
| C0A4. | Iodine-deficiency-related multinodular (endemic) goitre |
| C0A5. | Subclinical iodine-deficiency hypothyroidism |
| C0AX. | Iodine-deficiency-related (endemic) goitre, unspecified |
| C1343 | TSH - thyroid-stimulating hormone deficiency |
| C3A.. | Iodine-deficiency syndromes |
| Cyu1. | [X]Disorders of thyroid gland |
| Cyu10 | [X]Oth iodine-deficncy relatd thyroid disordr+allied condtns |
| Cyu11 | [X]Other sp cified hypothyroidism |
| Cyu12 | [X]Other specified nontoxic goitre |
| Cyu14 | [X]Other chronic thyroiditis |
| Cyu15 | [X]Other specified disorders of thyroid |
| Cyu4J | [X]Disorders of thyroid gland in diseases CE |
| F11x5 | Cerebral degeneration due to myxoedema |
| F1441 | Cerebellar ataxia due to myxoedema |
| F3814 | Myasthenic syndrome due to hypothyroidism |
| L1810 | Thyroid dysfunction - unspec whether in pregnancy/puerperium |
| PK251 | Congenital absence of thyroid gland |
| Q4337 | Neonatal jaundice with congenital hypothyroidism |
| 14G1. | H/O: rheumatoid arthritis |
| 669.. | Gout monitoring |
| 6691 | Initial gout assessment |
| 6692 | Follow-up gout assessment |
| 6693 | Joints gout affected |
| 6697 | Gout associated problems |
| 6699 | Gout treatment changed |
| 669Z. | Gout monitoring NOS |
| 66c0. | Disease modifying antirheumatic drug monitoring |
| AD61. | Behcet's syndrome |
| C34.. | Gout |
| C340. | Gouty arthropathy |
| C341. | Gouty nephropathy |
| C3410 | Gouty nephropathy unspecified |
| C3411 | Uric acid nephrolithiasis |
| C341z | Gouty nephropathy NOS |
| C342. | Idiopathic gout |
| C343. | Lead-induced gout |
| C344. | Drug-induced gout |
| C345. | Gout due to impairment of renal function |
| C34y. | Other specified gouty manifestation |
| C34y0 | Gouty tophi of ear |
| C34y1 | Gouty tophi of heart |
| C34y2 | Gouty tophi of other sites |
| C34y3 | Gouty iritis |
| C34y4 | Gouty neuritis |
| C34y5 | Gouty tophi of hand |
| C34yz | Other specified gouty manifestation NOS |
| C34z. | Gout NOS |
| C394. | Autoimmune disease NOS |
| F371. | Polyneuropathy in collagen vascular disease |
| F3710 | Polyneuropathy in disseminated lupus erythematosus |
| F3711 | Polyneuropathy in polyarteritis nodosa |
| F3712 | Polyneuropathy in rheumatoid arthritis |
| F371z | Polyneuropathy in collagen vascular disease NOS |
| F3961 | Myopathy due to disseminated lupus erythematosus |
| F3963 | Myopathy due to polyarteritis nodosa |
| F3964 | Myopathy due to rheumatoid arthritis |
| F3966 | Myopathy due to scleroderma |
| F3967 | Myopathy due to Sjogren's disease |
| F4A32 | Keratoconjunctivitis sicca (excluding Sjogren's syndrome) |
| G5573 | Gouty tophi of heart |
| G5yA. | Rheumatoid carditis |
| G5y8. | Rheumatoid myocarditis |
| G75.. | Polyarteritis nodosa and allied conditions |
| G750. | Polyarteritis nodosa |
| G751. | Acute febrile mucocutaneous lymph node syndrome |
| G7510 | Kawasaki disease |
| G751z | Acute febrile mucocutaneous lymph node syndrome NOS |
| G752. | Hypersensitivity angiitis |
| G7520 | Goodpasture's syndrome |
| G752z | Hypersensitivity angiitis NOS |
| G753. | Lethal midline granuloma |
| G754. | Wegener's granulomatosis |
| G755. | Giant cell arteritis |
| G7550 | Cranial arteritis |
| G7551 | Temporal arteritis |
| G7552 | Horton's disease |
| G755z | Giant cell arteritis NOS |
| G756. | Thrombotic microangiopathy |
| G7560 | Moschcowitz syndrome |
| G7561 | Thrombotic thrombocytopenic purpura |
| G756z | Thrombotic microangiopathy NOS |
| G757. | Takayasu's disease |
| G758. | Churg-Strauss vasculitis |
| G759. | Juvenile polyarteritis |
| G75X. | Necrotising vasculopathy, unspecified |
| G75z. | Polyarteritis nodosa and allied conditions NOS |
| H570. | Rheumatoid lung |
| H572. | Lung disease with systemic sclerosis |
| H57y1 | Lung disease with polymyositis |
| H57y3 | Lung disease with Sjogren's disease |
| H57y4 | Lung disease with systemic lupus erythematosus |
| K01x3 | Nephrotic syndrome in polyarteritis nodosa |
| K01x4 | Nephrotic syndrome in systemic lupus erythematosus |
| K0B40 | Renal tubulo-interstitial disorder in SLE |
| M1601 | Distal interphalangeal psoriatic arthropathy |
| M1602 | Arthritis mutilans |
| N00.. | Diffuse diseases of connective tissue |
| N000. | Systemic lupus erythematosus |
| N0000 | Disseminated lupus erythematosus |
| N0001 | Libman-Sacks disease |
| N0002 | Drug-induced systemic lupus erythematosus |
| N0003 | Systemic lupus erythematosus with organ or sys involv |
| N0004 | Systemic lupus erythematosus with pericarditis |
| N0005 | Neonatal lupus erythematosus |
| N000z | Systemic lupus erythematosus NOS |
| N001. | Scleroderma |
| N0010 | Progressive systemic sclerosis |
| N0011 | CREST syndrome |
| N0012 | Systemic sclerosis induced by drugs and chemicals |
| N002. | Sicca (Sjogren's) syndrome |
| N003. | Dermatomyositis |
| N0030 | Juvenile dermatomyositis |
| N0031 | Dermatopolymyositis in neoplastic disease |
| N003X | Dermatopolymyositis, unspecified |
| N004. | Polymyositis |
| N005. | Adult Still's Disease |
| N00y. | Other specified diffuse collagen diseases |
| N00y0 | Eosinophilic fasciitis |
| N00y1 | Fibrosclerosis systemic |
| N00z. | Collagen disease NOS |
| N012. | Arthropathy in Behcet's syndrome |
| N0120 | Arthropathy in Behcet's syndrome of unspecified site |
| N0121 | Arthropathy in Behcet's syndrome of the shoulder region |
| N0122 | Arthropathy in Behcet's syndrome of the upper arm |
| N0123 | Arthropathy in Behcet's syndrome of the forearm |
| N0124 | Arthropathy in Behcet's syndrome of the hand |
| N0125 | Arthropathy in Behcet's syndrome of the pelvis/thigh |
| N0126 | Arthropathy in Behcet's syndrome of the lower leg |
| N0127 | Arthropathy in Behcet's syndrome of the ankle and foot |
| N012x | Arthropathy in Behcet's syndrome of multiple sites |
| N012y | Arthropathy in Behcet's syndrome of other specified sites |
| N012z | Arthropathy in Behcet's syndrome NOS |
| N02.. | Crystal arthropathies |
| N020. | Chondrocalcinosis due to dicalcium phosphate crystals |
| N0200 | Chondrocalcinosis-dicalcium phosphate, of unspecified site |
| N0201 | Chondrocalcinosis-dicalcium phosphate, of shoulder region |
| N0202 | Chondrocalcinosis-dicalcium phosphate, of the upper arm |
| N0203 | Chondrocalcinosis-dicalcium phosphate, of the forearm |
| N0204 | Chondrocalcinosis-dicalcium phosphate, of the hand |
| N0205 | Chondrocalcinosis-dicalcium phosphate, of pelvic/thigh |
| N0206 | Chondrocalcinosis-dicalcium phosphate, of the lower leg |
| N0207 | Chondrocalcinosis-dicalcium phosphate, of the ankle and foot |
| N020x | Chondrocalcinosis-dicalcium phosphate, of multiple sites |
| N020y | Chondrocalcinosis-dicalcium phosphate, of other spec site |
| N020z | Chondrocalcinosis due to dicalcium phosphate crystals, NOS |
| N021. | Chondrocalcinosis due to pyrophosphate crystals |
| N0210 | Chondrocalcinosis-pyrophosphate crystals, of unspec site |
| N0211 | Chondrocalcinosis-pyrophosphate crystals, of shoulder region |
| N0212 | Chondrocalcinosis-pyrophosphate crystals, of the upper arm |
| N0213 | Chondrocalcinosis-pyrophosphate crystals, of the forearm |
| N0214 | Chondrocalcinosis-pyrophosphate crystals, of the hand |
| N0215 | Chondrocalcinosis-pyrophosphate crystals, of pelvic/thigh |
| N0216 | Chondrocalcinosis-pyrophosphate crystals, of the lower leg |
| N0217 | Chondrocalcinosis-pyrophosphate crystals, of ankle and foot |
| N021x | Chondrocalcinosis-pyrophosphate crystals, of multiple sites |
| N021y | Chondrocalcinosis-pyrophosphate crystals, of other spec site |
| N021z | Chondrocalcinosis due to pyrophosphate crystals, NOS |
| N022. | Chondrocalcinosis, unspecified |
| N0220 | Chondrocalcinosis unspecified, of unspecified site |
| N0221 | Chondrocalcinosis unspecified, of the shoulder region |
| N0222 | Chondrocalcinosis unspecified, of the upper arm |
| N0223 | Chondrocalcinosis unspecified, of the forearm |
| N0224 | Chondrocalcinosis unspecified, of the hand |
| N0225 | Chondrocalcinosis unspecified, of the pelvic region/thigh |
| N0226 | Chondrocalcinosis unspecified, of the lower leg |
| N0227 | Chondrocalcinosis unspecified, of the ankle and foot |
| N022x | Chondrocalcinosis unspecified, of multiple sites |
| N022y | Chondrocalcinosis unspecified, of other specified site |
| N022z | Chondrocalcinosis NOS |
| N023. | Gouty arthritis |
| N0230 | Gouty arthritis of unspecified site |
| N0231 | Gouty arthritis of the shoulder region |
| N0232 | Gouty arthritis of the upper arm |
| N0233 | Gouty arthritis of the forearm |
| N0234 | Gouty arthritis of the hand |
| N0235 | Gouty arthritis of the pelvic region and thigh |
| N0236 | Gouty arthritis of the lower leg |
| N0237 | Gouty arthritis of the ankle and foot |
| N0238 | Gouty arthritis of toe |
| N023x | Gouty arthritis of multiple sites |
| N023y | Gouty arthritis of other specified site |
| N023z | Gouty arthritis NOS |
| N024. | Familial chondrocalcinosis |
| N02y. | Other crystal arthropathies |
| N02y0 | Other crystal arthropathies of unspecified site |
| N02y1 | Other crystal arthropathies of the shoulder |
| N02y2 | Other crystal arthropathies of the upper arm |
| N02y3 | Other crystal arthropathies of the forearm |
| N02y4 | Other crystal arthropathies of the hand |
| N02y5 | Other crystal arthropathies of the pelvic region and thigh |
| N02y6 | Other crystal arthropathies of the lower leg |
| N02y7 | Other crystal arthropathies of the ankle and foot |
| N02y8 | Hydroxyapatite deposition disease |
| N02yx | Other crystal arthropathies of multiple sites |
| N02yy | Other crystal arthropathies of other specified sites |
| N02yz | Other crystal arthropathy NOS |
| N02z. | Crystal arthropathy NOS |
| N02z0 | Crystal arthropathy NOS, site unspecified |
| N02z1 | Crystal arthropathy NOS, of the shoulder region |
| N02z2 | Crystal arthropathy NOS, of the upper arm |
| N02z3 | Crystal arthropathy NOS, of the forearm |
| N02z4 | Crystal arthropathy NOS, of the hand |
| N02z5 | Crystal arthropathy NOS, of the pelvic region and thigh |
| N02z6 | Crystal arthropathy NOS, of the lower leg |
| N02z7 | Crystal arthropathy NOS, of the ankle and foot |
| N02z8 | Crystal arthropathy NOS, of shoulder |
| N02z9 | Crystal arthropathy NOS, of sternoclavicular joint |
| N02zA | Crystal arthropathy NOS, of acromioclavicular joint |
| N02zB | Crystal arthropathy NOS, of elbow |
| N02zC | Crystal arthropathy NOS, of distal radio-ulnar joint |
| N02zD | Crystal arthropathy NOS, of wrist |
| N02zE | Crystal arthropathy NOS, of MCP joint |
| N02zF | Crystal arthropathy NOS, of PIP joint of finger |
| N02zG | Crystal arthropathy NOS, of DIP joint of finger |
| N02zH | Crystal arthropathy NOS, of hip |
| N02zJ | Crystal arthropathy NOS, of sacro-iliac joint |
| N02zK | Crystal arthropathy NOS, of knee |
| N02zL | Crystal arthropathy NOS, of tibio-fibular joint |
| N02zM | Crystal arthropathy NOS, of ankle |
| N02zN | Crystal arthropathy NOS, of subtalar joint |
| N02zP | Crystal arthropathy NOS, of talonavicular joint |
| N02zQ | Crystal arthropathy NOS, of other tarsal joint |
| N02zR | Crystal arthropathy NOS, of 1st MTP joint |
| N02zS | Crystal arthropathy NOS, of lesser MTP joint |
| N02zT | Crystal arthropathy NOS, of IP joint of toe |
| N02zx | Crystal arthropathy NOS, of multiple sites |
| N02zy | Crystal arthropathy NOS, of other specified site |
| N02zz | Crystal arthropathy NOS |
| N03.. | Arthropathy associated with disorders EC |
| N030. | Arthropathy associated with endocrine and metabolic disorder |
| N0302 | Arthropathy in amyloidosis |
| N031. | Arthropathy assoc with non-infect gastrointestinal disorders |
| N032. | Arthropathy associated with haematological disorders |
| N036. | Arthropathy due to hypersensitivity reaction |
| N03x. | Other general diseases with associated arthropathy |
| N03x0 | Arthritis associated with other disease, shoulder |
| N03x1 | Arthritis associated with other disease, sternoclavic joint |
| N03x2 | Arthritis associated with other disease, acromioclavic joint |
| N03x3 | Arthritis associated with other disease, elbow |
| N03x4 | Arthritis associated with other disease, dist rad-uln joint |
| N03x5 | Arthritis associated with other disease, wrist |
| N03x6 | Arthritis associated with other disease, MCP joint |
| N03x7 | Arthritis associated with other disease, PIP joint of finger |
| N03x8 | Arthritis associated with other disease, DIP joint of finger |
| N03x9 | Arthritis associated with other disease, hip |
| N03xA | Arthritis associated with other disease, sacro-iliac joint |
| N03xB | Arthritis associated with other disease, knee |
| N03xC | Arthritis associated with other disease, tibio-fibular joint |
| N03xD | Arthritis associated with other disease, ankle |
| N03xE | Arthritis associated with other disease, subtalar joint |
| N03xF | Arthritis associated with other disease, talonavicular joint |
| N03xG | Arthritis associated with other disease, other tarsal joint |
| N03xH | Arthritis associated with other disease, 1st MTP joint |
| N03xJ | Arthritis associated with other disease, lesser MTP joint |
| N03xK | Arthritis associated with other disease, IP joint of toe |
| N03y. | Arthropathy associated with other conditions EC |
| N03z. | Arthropathy associated with disorders EC NOS |
| N0237 | Gouty arthritis of the ankle and foot |
| N0238 | Gouty arthritis of toe |
| N023x | Gouty arthritis of multiple sites |
| N023y | Gouty arthritis of other specified site |
| N023z | Gouty arthritis NOS |
| N024. | Familial chondrocalcinosis |
| N02y. | Other crystal arthropathies |
| N02y0 | Other crystal arthropathies of unspecified site |
| N02y1 | Other crystal arthropathies of the shoulder |
| N02y2 | Other crystal arthropathies of the upper arm |
| N02y3 | Other crystal arthropathies of the forearm |
| N02y4 | Other crystal arthropathies of the hand |
| N02y5 | Other crystal arthropathies of the pelvic region and thigh |
| N02y6 | Other crystal arthropathies of the lower leg |
| N02y7 | Other crystal arthropathies of the ankle and foot |
| N02y8 | Hydroxyapatite deposition disease |
| N02yx | Other crystal arthropathies of multiple sites |
| N02yy | Other crystal arthropathies of other specified sites |
| N02yz | Other crystal arthropathy NOS |
| N02z. | Crystal arthropathy NOS |
| N02z0 | Crystal arthropathy NOS, site unspecified |
| N02z1 | Crystal arthropathy NOS, of the shoulder region |
| N02z2 | Crystal arthropathy NOS, of the upper arm |
| N02z3 | Crystal arthropathy NOS, of the forearm |
| N02z4 | Crystal arthropathy NOS, of the hand |
| N02z5 | Crystal arthropathy NOS, of the pelvic region and thigh |
| N02z6 | Crystal arthropathy NOS, of the lower leg |
| N02z7 | Crystal arthropathy NOS, of the ankle and foot |
| N02z8 | Crystal arthropathy NOS, of shoulder |
| N02z9 | Crystal arthropathy NOS, of sternoclavicular joint |
| N02zA | Crystal arthropathy NOS, of acromioclavicular joint |
| N02zB | Crystal arthropathy NOS, of elbow |
| N02zC | Crystal arthropathy NOS, of distal radio-ulnar joint |
| N02zD | Crystal arthropathy NOS, of wrist |
| N02zE | Crystal arthropathy NOS, of MCP joint |
| N02zF | Crystal arthropathy NOS, of PIP joint of finger |
| N02zG | Crystal arthropathy NOS, of DIP joint of finger |
| N02zH | Crystal arthropathy NOS, of hip |
| N02zJ | Crystal arthropathy NOS, of sacro-iliac joint |
| N02zK | Crystal arthropathy NOS, of knee |
| N02zL | Crystal arthropathy NOS, of tibio-fibular joint |
| N02zM | Crystal arthropathy NOS, of ankle |
| N02zN | Crystal arthropathy NOS, of subtalar joint |
| N02zP | Crystal arthropathy NOS, of talonavicular joint |
| N02zQ | Crystal arthropathy NOS, of other tarsal joint |
| N02zR | Crystal arthropathy NOS, of 1st MTP joint |
| N02zS | Crystal arthropathy NOS, of lesser MTP joint |
| N02zT | Crystal arthropathy NOS, of IP joint of toe |
| N02zx | Crystal arthropathy NOS, of multiple sites |
| N02zy | Crystal arthropathy NOS, of other specified site |
| N02zz | Crystal arthropathy NOS |
| N03.. | Arthropathy associated with disorders EC |
| N030. | Arthropathy associated with endocrine and metabolic disorder |
| N0302 | Arthropathy in amyloidosis |
| N031. | Arthropathy assoc with non-infect gastrointestinal disorders |
| N032. | Arthropathy associated with haematological disorders |
| N036. | Arthropathy due to hypersensitivity reaction |
| N03x. | Other general diseases with associated arthropathy |
| N03x0 | Arthritis associated with other disease, shoulder |
| N03x1 | Arthritis associated with other disease, sternoclavic joint |
| N03x2 | Arthritis associated with other disease, acromioclavic joint |
| N03x3 | Arthritis associated with other disease, elbow |
| N03x4 | Arthritis associated with other disease, dist rad-uln joint |
| N03x5 | Arthritis associated with other disease, wrist |
| N03x6 | Arthritis associated with other disease, MCP joint |
| N03x7 | Arthritis associated with other disease, PIP joint of finger |
| N03x8 | Arthritis associated with other disease, DIP joint of finger |
| N03x9 | Arthritis associated with other disease, hip |
| N03xA | Arthritis associated with other disease, sacro-iliac joint |
| N03xB | Arthritis associated with other disease, knee |
| N03xC | Arthritis associated with other disease, tibio-fibular joint |
| N03xD | Arthritis associated with other disease, ankle |
| N03xE | Arthritis associated with other disease, subtalar joint |
| N03xF | Arthritis associated with other disease, talonavicular joint |
| N03xG | Arthritis associated with other disease, other tarsal joint |
| N03xH | Arthritis associated with other disease, 1st MTP joint |
| N03xJ | Arthritis associated with other disease, lesser MTP joint |
| N03xK | Arthritis associated with other disease, IP joint of toe |
| N03y. | Arthropathy associated with other conditions EC |
| N03z. | Arthropathy associated with disorders EC NOS |
| N023x | Gouty arthritis of multiple sites |
| N04.. | Rheumatoid arthritis and other inflammatory polyarthropathy |
| N040. | Rheumatoid arthritis |
| N0400 | Rheumatoid arthritis of cervical spine |
| N0401 | Other rheumatoid arthritis of spine |
| N0402 | Rheumatoid arthritis of shoulder |
| N0403 | Rheumatoid arthritis of sternoclavicular joint |
| N0404 | Rheumatoid arthritis of acromioclavicular joint |
| N0405 | Rheumatoid arthritis of elbow |
| N0406 | Rheumatoid arthritis of distal radio-ulnar joint |
| N0407 | Rheumatoid arthritis of wrist |
| N0408 | Rheumatoid arthritis of MCP joint |
| N0409 | Rheumatoid arthritis of PIP joint of finger |
| N040A | Rheumatoid arthritis of DIP joint of finger |
| N040B | Rheumatoid arthritis of hip |
| N040C | Rheumatoid arthritis of sacro-iliac joint |
| N040D | Rheumatoid arthritis of knee |
| N040E | Rheumatoid arthritis of tibio-fibular joint |
| N040F | Rheumatoid arthritis of ankle |
| N040G | Rheumatoid arthritis of subtalar joint |
| N040H | Rheumatoid arthritis of talonavicular joint |
| N040J | Rheumatoid arthritis of other tarsal joint |
| N040K | Rheumatoid arthritis of 1st MTP joint |
| N040L | Rheumatoid arthritis of lesser MTP joint |
| N040M | Rheumatoid arthritis of IP joint of toe |
| N040N | Rheumatoid vasculitis |
| N040P | Seronegative rheumatoid arthritis |
| N040Q | Rheumatoid bursitis |
| N040R | Rheumatoid nodule |
| N040S | Rheumatoid arthritis - multiple joint |
| N040T | Flare of rheumatoid arthritis |
| N041. | Felty's syndrome |
| N042. | Other rheumatoid arthropathy + visceral/systemic involvement |
| N0420 | Rheumatic carditis |
| N0421 | Rheumatoid lung disease |
| N0422 | Rheumatoid nodule |
| N042z | Rheumatoid arthropathy + visceral/systemic involvement NOS |
| N043. | Juvenile rheumatoid arthritis - Still's disease |
| N0430 | Juvenile rheumatoid arthropathy unspecified |
| N0431 | Acute polyarticular juvenile rheumatoid arthritis |
| N0432 | Pauciarticular juvenile rheumatoid arthritis |
| N0433 | Monarticular juvenile rheumatoid arthritis |
| N043z | Juvenile rheumatoid arthritis NOS |
| N0451 | Juvenile seronegative polyarthritis |
| N0452 | Juvenile arthritis in psoriasis |
| N0453 | Juvenile arthritis in Crohn's disease |
| N0454 | Juvenile arthritis in ulcerative colitis |
| N0455 | Juvenile rheumatoid arthritis |
| N0456 | Pauciarticular onset juvenile chronic arthritis |
| N047. | Seropositive errosive rheumatoid arthritis |
| N04X. | Seropositive rheumatoid arthritis, unspecified |
| N04y. | Other specified inflammatory polyarthropathy |
| N04y2 | Adult-onset Still's disease |
| N04y3 | Remitting seronegative symmetrical synovitis pitting oedema |
| N04yz | Other specified inflammatory polyarthropathy NOS |
| N04z. | Inflammatory polyarthropathy NOS |
| N0505 | Secondary multiple arthrosis |
| N060. | Kaschin - Beck disease |
| N0600 | Kaschin-Beck disease of unspecified site |
| N0601 | Kaschin-Beck disease of the shoulder region |
| N0602 | Kaschin-Beck disease of the upper arm |
| N0603 | Kaschin-Beck disease of the forearm |
| N0604 | Kaschin-Beck disease of the hand |
| N0605 | Kaschin-Beck disease of the pelvic region and thigh |
| N0606 | Kaschin-Beck disease of the lower leg |
| N0607 | Kaschin-Beck disease of the ankle and foot |
| N0608 | Kaschin-Beck disease of other specified site |
| N0609 | Kaschin-Beck disease of multiple sites |
| N060z | Kaschin-Beck disease NOS |
| N062. | Allergic arthritis |
| N0620 | Allergic arthritis of unspecified site |
| N0621 | Allergic arthritis of the shoulder region |
| N0622 | Allergic arthritis of the upper arm |
| N0623 | Allergic arthritis of the forearm |
| N0624 | Allergic arthritis of the hand |
| N0625 | Allergic arthritis of the pelvic region and thigh |
| N0626 | Allergic arthritis of the lower leg |
| N0627 | Allergic arthritis of the ankle and foot |
| N0628 | Allergic arthritis of other specified site |
| N0629 | Allergic arthritis of multiple sites |
| N062z | Allergic arthritis NOS |
| N063. | Climacteric arthritis |
| N0630 | Climacteric arthritis of unspecified site |
| N0631 | Climacteric arthritis of the shoulder region |
| N0632 | Climacteric arthritis of the upper arm |
| N0633 | Climacteric arthritis of the forearm |
| N0634 | Climacteric arthritis of the hand |
| N0635 | Climacteric arthritis of the pelvic region and thigh |
| N0636 | Climacteric arthritis of the lower leg |
| N0637 | Climacteric arthritis of the ankle and foot |
| N0638 | Climacteric arthritis of other specified site |
| N0639 | Climacteric arthritis of multiple sites |
| N063z | Climacteric arthritis NOS |
| N065. | Unspecified polyarthropathy or polyarthritis |
| N0650 | Unspecified polyarthropathy of unspecified site |
| N0651 | Unspecified polyarthropathy of the shoulder region |
| N0652 | Unspecified polyarthropathy of the upper arm |
| N0653 | Unspecified polyarthropathy of the forearm |
| N0654 | Unspecified polyarthropathy of the hand |
| N0655 | Unspecified polyarthropathy of the pelvic region and thigh |
| N0656 | Unspecified polyarthropathy of the lower leg |
| N0657 | Unspecified polyarthropathy of the ankle and foot |
| N0658 | Unspecified polyarthropathy of other specified site |
| N0659 | Unspecified polyarthropathy of multiple sites |
| N065A | Generalised arthritis |
| N065z | Unspecified polyarthropathy or polyarthritis NOS |
| N068. | Haemophilic arthropathy |
| N069. | Arthropathy in neoplastic disease |
| N06y. | Other specified arthropathy |
| N06y0 | Other specified arthropathy of unspecified site |
| N06y1 | Other specified arthropathy of the shoulder region |
| N06y2 | Other specified arthropathy of the upper arm |
| N06y3 | Other specified arthropathy of the forearm |
| N06y4 | Other specified arthropathy of the hand |
| N06y5 | Other specified arthropathy of the pelvic region and thigh |
| N06y6 | Other specified arthropathy of the lower leg |
| N06y7 | Other specified arthropathy of the ankle and foot |
| N06y8 | Other specified arthropathy of other specified site |
| N06y9 | Other specified arthropathy of multiple sites |
| N06yz | Other specified arthropathy NOS |
| N06zA | Acute arthritis |
| N06zB | Chronic arthritis |
| N06zz | Arthropathy NOS |
| N090W | Intermittent hydrarthrosis |
| N092. | Villonodular synovitis |
| N0920 | Villonodular synovitis of unspecified site |
| N0921 | Villonodular synovitis of the shoulder region |
| N0922 | Villonodular synovitis of the upper arm |
| N0923 | Villonodular synovitis of the forearm |
| N0924 | Villonodular synovitis of the hand |
| N0925 | Villonodular synovitis of the pelvic region and thigh |
| N0926 | Villonodular synovitis of the lower leg |
| N0927 | Villonodular synovitis of the ankle and foot |
| N0928 | Villonodular synovitis of other specified site |
| N0929 | Villonodular synovitis of multiple sites |
| N092A | Villonodular synovitis of shoulder joint |
| N092B | Villonodular synovitis of sternoclavicular joint |
| N092C | Villonodular synovitis of acromioclavicular joint |
| N092D | Villonodular synovitis of elbow |
| N092E | Villonodular synovitis of distal radio-ulnar joint |
| N092F | Villonodular synovitis of wrist |
| N092G | Villonodular synovitis of MCP joint |
| N092H | Villonodular synovitis of PIP joint of finger |
| N092J | Villonodular synovitis of DIP joint of finger |
| N092K | Villonodular synovitis of hip |
| N092L | Villonodular synovitis of sacro-iliac joint |
| N092M | Villonodular synovitis of knee |
| N092N | Villonodular synovitis of tibio-fibular joint |
| N092P | Villonodular synovitis of ankle |
| N092Q | Villonodular synovitis of subtalar joint |
| N092R | Villonodular synovitis of talonavicular joint |
| N092S | Villonodular synovitis of other tarsal joint |
| N092T | Villonodular synovitis of 1st MTP joint |
| N092U | Villonodular synovitis of lesser MTP joint |
| N092V | Villonodular synovitis of IP joint of toe |
| N092z | Villonodular synovitis NOS |
| N093. | Palindromic rheumatism |
| N0930 | Palindromic rheumatism of unspecified site |
| N0931 | Palindromic rheumatism of the shoulder region |
| N0932 | Palindromic rheumatism of the upper arm |
| N0933 | Palindromic rheumatism of the forearm |
| N0934 | Palindromic rheumatism of the hand |
| N0935 | Palindromic rheumatism of the pelvic region and thigh |
| N0936 | Palindromic rheumatism of the lower leg |
| N0937 | Palindromic rheumatism of the ankle and foot |
| N0938 | Palindromic rheumatism of other specified site |
| N0939 | Palindromic rheumatism of multiple sites |
| N093z | Palindromic rheumatism NOS |
| N0y.. | Other specified arthropathies |
| N0z.. | Arthropathies NOS |
| N20.. | Polymyalgia rheumatica |
| N200. | Giant cell arteritis with polymyalgia rheumatica |
| N2208 | Villonodular synovitis of tendon sheath |
| N235. | Hypermobility syndrome |
| N2432 | Weber - Christian disease |
| Nyu00 | [X]Other streptococcal arthritis and polyarthritis |
| Nyu1. | [X]Inflammatory polyarthropathies |
| Nyu10 | [X]Rheumatoid arthritis+involvement/other organs or systems |
| Nyu11 | [X]Other seropositive rheumatoid arthritis |
| Nyu12 | [X]Other specified rheumatoid arthritis |
| Nyu13 | [X]Other psoriatic arthropathies |
| Nyu14 | [X]Other enteropathic arthropathies |
| Nyu15 | [X]Other juvenile arthritis |
| Nyu16 | [X]Juvenile arthritis in other diseases CE |
| Nyu17 | [X]Other secondary gout |
| Nyu18 | [X]Other chondrocalcinosis |
| Nyu19 | [X]Other specified crystal arthropathies |
| Nyu1A | [X]Other specific arthropathies, not elsewhere classified |
| Nyu1B | [X]Other specified arthritis |
| Nyu1C | [X]Gouty arthrpathy due/enzym defects+oth inherit disordr CE |
| Nyu1D | [X]Crystal arthropathy in other metabolic disorders CE |
| Nyu1E | [X]Arthropathies/oth endocrin,nutritionl+metabolic disorders |
| Nyu1F | [X]Arthropathies in other specified diseases CE |
| Nyu1G | [X]Seropositive rheumatoid arthritis, unspecified |
| Nyu4. | [X]Systemic connective tissue disorders |
| Nyu40 | [X]Other conditions related to polyarteritis nodosa |
| Nyu41 | [X]Other giant cell arteritis |
| Nyu42 | [X]Other specified necrotizing vasculopathies |
| Nyu43 | [X]Other forms of systemic lupus erythematosus |
| Nyu44 | [X]Other dermatomyositis |
| Nyu45 | [X]Other forms of systemic sclerosis |
| Nyu46 | [X]Other overlap syndromes |
| Nyu47 | [X]Other systemic diseases of connective tissue |
| Nyu48 | [X]Dermato(poly)myositis in neoplastic disease CE |
| Nyu49 | [X]Arthropathy in neoplastic disease classified elsewhere |
| Nyu4A | [X]Arthropathy in other blood disorders CE |
| Nyu4B | [X]Arthropathy in hypersensitivity reactions CE |
| Nyu4C | [X]Systemic disorders/connective tissue in other diseases CE |
| Nyu4D | [X]Necrotising vasculopathy, unspecified |
| Nyu4E | [X]Dermatopolymyositis, unspecified |
| Nyu4F | [X]Mixed connective tissue disease |
| 1493 | H/O: hearing problem |
| 1C12. | Hearing difficulty |
| 1C13. | Deafness |
| 1C131 | Unilateral deafness |
| 1C132 | Partial deafness |
| 1C133 | Bilateral deafness |
| 1C16. | Deteriorating hearing |
| 1C18. | Difficulty hearing with background noise |
| 1C19. | Difficulty hearing whispers |
| 1C1Z. | Hearing symptom NOS |
| 2BL2. | O/E - slightly deaf |
| 2BL3. | O/E - significantly deaf |
| 2BL4. | O/E - very deaf |
| 2BL5. | O/E - completely deaf |
| 2BM2. | O/E -tune fork=conductive deaf |
| 2BM3. | O/E tune fork=perceptive deaf |
| 2BM4. | O/E - High tone deafness |
| 2DG.. | Hearing aid worn |
| 2DH0. | Uses hearing loop |
| 31343 | Hearing test abnormal |
| 31344 | Hearing test bilateral abnormality |
| 31345 | Hearing test left abnormality |
| 31346 | Hearing test right abnormality |
| F5801 | Presbyacusis |
| F5812 | Noise-induced hearing loss |
| F582. | Unspecified sudden hearing loss |
| F59.. | Hearing loss |
| F590. | Conductive hearing loss |
| F5900 | Unspecified conductive hearing loss |
| F5901 | Conductive hearing loss due to disorder of external ear |
| F5902 | Conductive hearing loss due to disorder of tympanic membrane |
| F5903 | Conductive hearing loss due to disorder of middle ear |
| F5904 | Conductive hearing loss due to disorder of inner ear |
| F5905 | Conductive hearing loss, bilateral |
| F5906 | Conduct hear loss,unilat+unrestric hearing on contralat side |
| F590y | Combined conductive hearing loss |
| F590z | Conductive hearing loss NOS |
| F591. | Sensorineural hearing loss |
| F5910 | Unspecified perceptive hearing loss |
| F5911 | Sensory hearing loss |
| F5912 | Neural hearing loss |
| F5913 | Central hearing loss |
| F5914 | Congenital sensorineural deafness |
| F5915 | Ototoxicity - deafness |
| F5916 | Sensorineural hearing loss, bilateral |
| F5917 | Sensorineurl hear loss,unilat unrestrict hear/contralat side |
| F5918 | Congenital prelingual deafness |
| F591y | Combined perceptive hearing loss |
| F591z | Perceptive hearing loss NOS |
| F592. | Mixed conductive and sensorineural deafness |
| F5920 | Mix cond/sensneurl hear loss,unlat unrestrc hear/contrlat sd |
| F5921 | Mixed conductive and sensorineural hearing loss, bilateral |
| F593. | Deaf mutism, NEC |
| F594. | High frequency deafness |
| F595. | Low frequency deafness |
| F596. | Maternally inherited deafness |
| F597. | Mild acquired hearing loss |
| F598. | Moderate acquired hearing loss |
| F599. | Severe acquired hearing loss |
| F59A. | Profound acquired hearing loss |
| F59y. | Other specified forms of hearing loss |
| F59z. | Deafness NOS |
| F5A.. | Hearing impairment |
| FyuU0 | [X]Deaf mutism, not elsewhere classified |
| FyuU1 | [X]Other specified hearing loss |
| P40.. | Ear anomalies with hearing impairment |
| P400. | Ear anomalies with hearing impaired, unspecified |
| P402. | Other external ear anomaly with hearing impairment |
| P402z | Other external ear anomaly with hearing impairment NOS |
| P40z. | Other and unspecified ear anomaly with hearing impaired |
| P40zz | Ear anomaly with hearing impaired NOS |
| ZE63. | Hearing worse |
| ZE7.. | Hearing problem |
| ZE812 | Hearing for whisper impaired |
| ZE813 | Unable to hear whisper |
| ZE822 | Hearing for conversational voice impaired |
| ZE823 | Unable to hear conversational voice |
| ZE832 | Hearing for loud voice impaired |
| ZE833 | Unable to hear loud voice |
| ZE842 | Hearing for voice impaired |
| ZE843 | Ability to hear voice abnormal |
| ZE86. | Difficulty hearing in noise |
| ZE87. | Hearing loss |
| ZV412 | [V]Problems with hearing |
| 66YB. | Chronic obstructive pulmonary disease monitoring |
| 66Yd. | COPD accident and emergency attendance since last visit |
| 66YD. | Chronic obstructive pulmonary disease monitoring due |
| 66Ye. | Emergency COPD admission since last appointment |
| 66Yf. | Number of COPD exacerbations in past year |
| 66Yg. | Chronic obstructive pulmonary disease disturbs sleep |
| 66Yh. | Chronic obstructive pulmonary disease does not disturb sleep |
| 66Yi. | Multiple COPD emergency hospital admissions |
| 66YI. | COPD self-management plan given |
| 66YL. | Chronic obstructive pulmonary disease follow-up |
| 66YM. | Chronic obstructive pulmonary disease annual review |
| 66YS. | Chronic obstructive pulmonary disease monitoring by nurse |
| 66YT. | Chronic obstructive pulmonary disease monitoring by doctor |
| 8CR1. | Chronic obstructive pulmonary disease clini management plan |
| H3... | Chronic obstructive pulmonary disease |
| H30.. | Bronchitis unspecified |
| H300. | Tracheobronchitis NOS |
| H301. | Laryngotracheobronchitis |
| H302. | Wheezy bronchitis |
| H30z. | Bronchitis NOS |
| H31.. | Chronic bronchitis |
| H310. | Simple chronic bronchitis |
| H3100 | Chronic catarrhal bronchitis |
| H3101 | Smokers' cough |
| H310z | Simple chronic bronchitis NOS |
| H311. | Mucopurulent chronic bronchitis |
| H3110 | Purulent chronic bronchitis |
| H3111 | Fetid chronic bronchitis |
| H311z | Mucopurulent chronic bronchitis NOS |
| H312. | Obstructive chronic bronchitis |
| H3120 | Chronic asthmatic bronchitis |
| H3121 | Emphysematous bronchitis |
| H3122 | Acute exacerbation of chronic obstructive airways disease |
| H312z | Obstructive chronic bronchitis NOS |
| H313. | Mixed simple and mucopurulent chronic bronchitis |
| H31y. | Other chronic bronchitis |
| H31y0 | Chronic tracheitis |
| H31y1 | Chronic tracheobronchitis |
| H31yz | Other chronic bronchitis NOS |
| H31z. | Chronic bronchitis NOS |
| H32.. | Emphysema |
| H320. | Chronic bullous emphysema |
| H3200 | Segmental bullous emphysema |
| H3201 | Zonal bullous emphysema |
| H3202 | Giant bullous emphysema |
| H3203 | Bullous emphysema with collapse |
| H320z | Chronic bullous emphysema NOS |
| H321. | Panlobular emphysema |
| H322. | Centrilobular emphysema |
| H32y. | Other emphysema |
| H32y0 | Acute vesicular emphysema |
| H32y1 | Atrophic (senile) emphysema |
| H32y2 | MacLeod's unilateral emphysema |
| H32yz | Other emphysema NOS |
| H32z. | Emphysema NOS |
| H36.. | Mild chronic obstructive pulmonary disease |
| H37.. | Moderate chronic obstructive pulmonary disease |
| H38.. | Severe chronic obstructive pulmonary disease |
| H39.. | Very severe chronic obstructive pulmonary disease |
| H3y.. | Other specified chronic obstructive airways disease |
| H3y0. | Chronic obstruct pulmonary dis with acute lower resp infectn |
| H3y1. | Chron obstruct pulmonary dis wth acute exacerbation, unspec |
| H3z.. | Chronic obstructive airways disease NOS |
| H4640 | Chronic emphysema due to chemical fumes |
| H4641 | Obliterative bronchiolitis due to chemical fumes |
| Hyu3. | [X]Chronic lower respiratory diseases |
| Hyu30 | [X]Other emphysema |
| Hyu31 | [X]Other specified chronic obstructive pulmonary disease |
| 285.. | Neurotic condition, insight present |
| 286.. | Poor insight into neurotic condition |
| 1466 | H/O: anxiety state |
| 1B13. | Anxiousness |
| 225J. | O/E - panic attack |
| 8G94. | Anxiety management training |
| E2... | Neurotic, personality and other nonpsychotic disorders |
| E20.. | Neurotic disorders |
| E200. | Anxiety states |
| E2000 | Anxiety state unspecified |
| E2001 | Panic disorder |
| E2002 | Generalised anxiety disorder |
| E2003 | Anxiety with depression |
| E2004 | Chronic anxiety |
| E2005 | Recurrent anxiety |
| E200z | Anxiety state NOS |
| E201. | Hysteria |
| E2010 | Hysteria unspecified |
| E2011 | Hysterical blindness |
| E2012 | Hysterical deafness |
| E2013 | Hysterical tremor |
| E2014 | Hysterical paralysis |
| E2015 | Hysterical seizures |
| E2016 | Other conversion disorder |
| E2017 | Hysterical amnesia |
| E2018 | Hysterical fugue |
| E2019 | Multiple personality |
| E201A | Dissociative reaction unspecified |
| E201B | Compensation neurosis |
| E201C | Phantom pregnancy |
| E201z | Hysteria NOS |
| E202. | Phobic disorders |
| E2020 | Phobia unspecified |
| E2021 | Agoraphobia with panic attacks |
| E2022 | Agoraphobia without mention of panic attacks |
| E2023 | Social phobia, fear of eating in public |
| E2024 | Social phobia, fear of public speaking |
| E2025 | Social phobia, fear of public washing |
| E2026 | Acrophobia |
| E2027 | Animal phobia |
| E2028 | Claustrophobia |
| E2029 | Fear of crowds |
| E202A | Fear of flying |
| E202B | Cancer phobia |
| E202C | Dental phobia |
| E202D | Fear of death |
| E202E | Fear of pregnancy |
| E202z | Phobic disorder NOS |
| E203. | Obsessive-compulsive disorders |
| E2030 | Compulsive neurosis |
| E2031 | Obsessional neurosis |
| E203z | Obsessive-compulsive disorder NOS |
| E204. | Neurotic depression reactive type |
| E205. | Neurasthenia - nervous debility |
| E206. | Depersonalisation syndrome |
| E207. | Hypochondriasis |
| E20y. | Other neurotic disorders |
| E20y0 | Somatization disorder |
| E20y1 | Writer's cramp neurosis |
| E20y2 | Other occupational neurosis |
| E20y3 | Psychasthenic neurosis |
| E20yz | Other neurotic disorder NOS |
| E20z. | Neurotic disorder NOS |
| E21.. | Personality disorders |
| E210. | Paranoid personality disorder |
| E211. | Affective personality disorder |
| E2110 | Unspecified affective personality disorder |
| E2111 | Hypomanic personality disorder |
| E2112 | Depressive personality disorder |
| E2113 | Cyclothymic personality disorder |
| E211z | Affective personality disorder NOS |
| E212. | Schizoid personality disorder |
| E2120 | Unspecified schizoid personality disorder |
| E2121 | Introverted personality |
| E2122 | Schizotypal personality |
| E212z | Schizoid personality disorder NOS |
| E213. | Explosive personality disorder |
| E214. | Compulsive personality disorders |
| E2140 | Anankastic personality |
| E2141 | Obsessional personality |
| E214z | Compulsive personality disorder NOS |
| E215. | Histrionic personality disorders |
| E2150 | Unspecified histrionic personality disorder |
| E2151 | Munchausen's syndrome |
| E2152 | Emotionally unstable personality |
| E2153 | Psychoinfantile personality |
| E215z | Histrionic personality disorder NOS |
| E216. | Inadequate personality disorder |
| E217. | Antisocial or sociopathic personality disorder |
| E21y. | Other personality disorders |
| E21y0 | Narcissistic personality disorder |
| E21y1 | Avoidant personality disorder |
| E21y2 | Borderline personality disorder |
| E21y3 | Passive-aggressive personality disorder |
| E21y4 | Eccentric personality disorder |
| E21y5 | Immature personality disorder |
| E21y6 | Masochistic personality disorder |
| E21y7 | Psychoneurotic personality disorder |
| E21yz | Other personality disorder NOS |
| E21z. | Personality disorder NOS |
| E26.. | Physiological malfunction arising from mental factors |
| E260. | Psychogenic musculoskeletal symptoms |
| E2600 | Psychogenic paralysis |
| E2601 | Psychogenic torticollis |
| E260z | Psychogenic musculoskeletal symptoms NOS |
| E261. | Psychogenic respiratory symptoms |
| E2610 | Psychogenic air hunger |
| E2611 | Psychogenic cough |
| E2612 | Psychogenic hiccough |
| E2613 | Psychogenic hyperventilation |
| E2614 | Psychogenic yawning |
| E2615 | Psychogenic aphonia |
| E261z | Psychogenic respiratory symptom NOS |
| E262. | Psychogenic cardiovascular symptoms |
| E2620 | Cardiac neurosis |
| E2621 | Cardiovascular neurosis |
| E2622 | Neurocirculatory asthenia |
| E2623 | Psychogenic cardiovascular disorder |
| E262z | Psychogenic cardiovascular symptom NOS |
| E263. | Psychogenic skin symptoms |
| E2630 | Psychogenic pruritus |
| E263z | Psychogenic skin symptoms NOS |
| E264. | Psychogenic gastrointestinal tract symptoms |
| E2640 | Psychogenic aerophagy |
| E2642 | Cyclical vomiting - psychogenic |
| E2643 | Psychogenic diarrhoea |
| E2644 | Psychogenic dyspepsia |
| E2645 | Psychogenic constipation |
| E264z | Psychogenic gastrointestinal tract symptom NOS |
| E265. | Psychogenic genitourinary tract symptoms |
| E2650 | Psychogenic genitourinary tract malfunction unspecified |
| E2651 | Psychogenic vaginismus |
| E2652 | Psychogenic dysmenorrhea |
| E2653 | Psychogenic dysuria |
| E265z | Psychogenic genitourinary tract symptom NOS |
| E266. | Psychogenic endocrine malfunction |
| E267. | Psychogenic symptom of special sense organ |
| E26y. | Other psychogenic malfunction |
| E26y0 | Bruxism (teeth grinding) |
| E26yz | Other psychogenic malfunction NOS |
| E26z. | Psychosomatic disorder NOS |
| E278. | Psychalgia |
| E2780 | Psychogenic pain unspecified |
| E2781 | Tension headache |
| E2782 | Psychogenic backache |
| E278z | Psychalgia NOS |
| E28.. | Acute reaction to stress |
| E280. | Acute panic state due to acute stress reaction |
| E281. | Acute fugue state due to acute stress reaction |
| E282. | Acute stupor state due to acute stress reaction |
| E283. | Other acute stress reactions |
| E2830 | Acute situational disturbance |
| E2831 | Acute posttrauma stress state |
| E283z | Other acute stress reaction NOS |
| E284. | Stress reaction causing mixed disturbance of emotion/conduct |
| E28z. | Acute stress reaction NOS |
| E29.. | Adjustment reaction |
| E2900 | Grief reaction |
| E2920 | Separation anxiety disorder |
| E2921 | Adolescent emancipation disorder |
| E2922 | Early adult emancipation disorder |
| E2923 | Specific academic or work inhibition |
| E2924 | Adjustment reaction with anxious mood |
| E2925 | Culture shock |
| E292y | Adjustment reaction with mixed disturbance of emotion |
| E292z | Adjustment reaction with disturbance of other emotion NOS |
| E293. | Adjustment reaction with predominant disturbance of conduct |
| E2930 | Adjustment reaction with aggression |
| E2931 | Adjustment reaction with antisocial behaviour |
| E2932 | Adjustment reaction with destructiveness |
| E293z | Adjustment reaction with predominant disturbance conduct NOS |
| E294. | Adjustment reaction with disturbance emotion and conduct |
| E29y. | Other adjustment reactions |
| E29y0 | Concentration camp syndrome |
| E29y1 | Other post-traumatic stress disorder |
| E29y2 | Adjustment reaction with physical symptoms |
| E29y3 | Elective mutism due to an adjustment reaction |
| E29y4 | Adjustment reaction due to hospitalisation |
| E29y5 | Other adjustment reaction with withdrawal |
| E29yz | Other adjustment reactions NOS |
| E29z. | Adjustment reaction NOS |
| E292. | Adjustment reaction, predominant disturbance other emotions |
| Eu054 | [X]Organic anxiety disorder |
| Eu4.. | [X]Neurotic, stress - related and somoform disorders |
| Eu40. | [X]Phobic anxiety disorders |
| Eu400 | [X]Agoraphobia |
| Eu401 | [X]Social phobias |
| Eu402 | [X]Specific (isolated) phobias |
| Eu403 | [X]Needle phobia |
| Eu40y | [X]Other phobic anxiety disorders |
| Eu40z | [X]Phobic anxiety disorder, unspecified |
| Eu41. | [X]Other anxiety disorders |
| Eu410 | [X]Panic disorder [episodic paroxysmal anxiety] |
| Eu411 | [X]Generalized anxiety disorder |
| Eu412 | [X]Mixed anxiety and depressive disorder |
| Eu413 | [X]Other mixed anxiety disorders |
| Eu41y | [X]Other specified anxiety disorders |
| Eu41z | [X]Anxiety disorder, unspecified |
| Eu42. | [X]Obsessive - compulsive disorder |
| Eu420 | [X]Predominantly obsessional thoughts or ruminations |
| Eu421 | [X]Predominantly compulsive acts [obsessional rituals] |
| Eu422 | [X]Mixed obsessional thoughts and acts |
| Eu42y | [X]Other obsessive-compulsive disorders |
| Eu42z | [X]Obsessive-compulsive disorder, unspecified |
| Eu43. | [X]Reaction to severe stress, and adjustment disorders |
| Eu430 | [X]Acute stress reaction |
| Eu431 | [X]Post - traumatic stress disorder |
| Eu432 | [X]Adjustment disorders |
| Eu43y | [X]Other reactions to severe stress |
| Eu43z | [X]Reaction to severe stress, unspecified |
| Eu44. | [X]Dissociative [conversion] disorders |
| Eu440 | [X]Dissociative amnesia |
| Eu441 | [X]Dissociative fugue |
| Eu442 | [X]Dissociative stupor |
| Eu443 | [X]Trance and possession disorders |
| Eu444 | [X]Dissociative motor disorders |
| Eu445 | [X]Dissociative convulsions |
| Eu446 | [X]Dissociative anaesthesia and sensory loss |
| Eu447 | [X]Mixed dissociative [conversion] disorders |
| Eu44y | [X]Other dissociative [conversion] disorders |
| Eu44z | [X]Dissociative [conversion] disorder, unspecified |
| Eu45. | [X]Somatoform disorders |
| Eu450 | [X]Somatization disorder |
| Eu451 | [X]Undifferentiated somatoform disorder |
| Eu452 | [X]Hypochondriacal disorder |
| Eu453 | [X]Somatoform autonomic dysfunction |
| Eu454 | [X]Persistent somatoform pain disorder |
| Eu455 | [X]Globus pharyngeus |
| Eu45y | [X]Other somatoform disorders |
| Eu45z | [X]Somatoform disorder, unspecified |
| Eu46. | [X]Other neurotic disorders |
| Eu460 | [X]Neurasthenia |
| Eu461 | [X]Depersonalization - derealization syndrome |
| Eu46y | [X]Other specified neurotic disorders |
| Eu46z | [X]Neurotic disorder, unspecified |
| Eu930 | [X]Separation anxiety disorder of childhood |
| Eu931 | [X]Phobic anxiety disorder of childhood |
| Eu932 | [X]Social anxiety disorder of childhood |
| M240E | Alopecia neurotica |
| Eu45. | [X]Somatoform disorders |
| Eu450 | [X]Somatization disorder |
| Eu451 | [X]Undifferentiated somatoform disorder |
| Eu455 | [X]Globus pharyngeus |
| Eu45y | [X]Other somatoform disorders |
| Eu45z | [X]Somatoform disorder, unspecified |
| Eu46. | [X]Other neurotic disorders |
| ZN114 | Anxiety Management |
| ZS7C7 | Post-traumatic mutism |
| 14CF. | History of irritable bowel syndrome |
| J521. | Irritable colon - Irritable bowel syndrome |
| J5210 | Irritable bowel syndrome with diarrhoea |
| J529. | Generalised intestinal dysmotility |
| J52y. | Other functional disorders of the intestine |
| J52yz | Other functional disorder of the intestine NOS |
| J52z. | Functional gastrointestinal tract disorders NEC NOS |
| Jyu53 | [X]Other specified functional disorders of intestine |
| 1O0.. | Cancer confirmed |
| 7G03K | Excision malignant skin tumour |
| A7886 | Human immunodeficiency virus with secondary cancers |
| A788W | HIV disease resulting in unspecified malignant neoplasm |
| A7898 | HIV disease resulting in multiple malignant neoplasms |
| B0... | Malignant neoplasm of lip, oral cavity and pharynx |
| B00.. | Malignant neoplasm of lip |
| B000. | Malignant neoplasm of upper lip, vermilion border |
| B0000 | Malignant neoplasm of upper lip, external |
| B0001 | Malignant neoplasm of upper lip, lipstick area |
| B000z | Malignant neoplasm of upper lip, vermilion border NOS |
| B001. | Malignant neoplasm of lower lip, vermilion border |
| B0010 | Malignant neoplasm of lower lip, external |
| B0011 | Malignant neoplasm of lower lip, lipstick area |
| B001z | Malignant neoplasm of lower lip, vermilion border NOS |
| B002. | Malignant neoplasm of upper lip, inner aspect |
| B0020 | Malignant neoplasm of upper lip, buccal aspect |
| B0021 | Malignant neoplasm of upper lip, frenulum |
| B0022 | Malignant neoplasm of upper lip, mucosa |
| B0023 | Malignant neoplasm of upper lip, oral aspect |
| B002z | Malignant neoplasm of upper lip, inner aspect NOS |
| B003. | Malignant neoplasm of lower lip, inner aspect |
| B0030 | Malignant neoplasm of lower lip, buccal aspect |
| B0031 | Malignant neoplasm of lower lip, frenulum |
| B0032 | Malignant neoplasm of lower lip, mucosa |
| B0033 | Malignant neoplasm of lower lip, oral aspect |
| B003z | Malignant neoplasm of lower lip, inner aspect NOS |
| B004. | Malignant neoplasm of lip unspecified, inner aspect |
| B0040 | Malignant neoplasm of lip unspecified, buccal aspect |
| B0041 | Malignant neoplasm of lip unspecified, frenulum |
| B0042 | Malignant neoplasm of lip unspecified, mucosa |
| B0043 | Malignant neoplasm of lip, oral aspect |
| B004z | Malignant neoplasm of lip, inner aspect NOS |
| B005. | Malignant neoplasm of commissure of lip |
| B006. | Malignant neoplasm of overlapping lesion of lip |
| B007. | Malignant neoplasm of lip, unspecified |
| B00y. | Malignant neoplasm of other sites of lip |
| B00z. | Malignant neoplasm of vermilion border of lip unspecified |
| B00z0 | Malignant neoplasm of lip, unspecified, external |
| B00z1 | Malignant neoplasm of lip, unspecified, lipstick area |
| B00zz | Malignant neoplasm of lip, vermilion border NOS |
| B01.. | Malignant neoplasm of tongue |
| B010. | Malignant neoplasm of base of tongue |
| B0100 | Malignant neoplasm of base of tongue dorsal surface |
| B010z | Malignant neoplasm of fixed part of tongue NOS |
| B011. | Malignant neoplasm of dorsal surface of tongue |
| B0110 | Malignant neoplasm of anterior 2/3 of tongue dorsal surface |
| B0111 | Malignant neoplasm of midline of tongue |
| B011z | Malignant neoplasm of dorsum of tongue NOS |
| B012. | Malignant neoplasm of tongue, tip and lateral border |
| B013. | Malignant neoplasm of ventral surface of tongue |
| B0130 | Malignant neoplasm of anterior 2/3 of tongue ventral surface |
| B0131 | Malignant neoplasm of frenulum linguae |
| B013z | Malignant neoplasm of ventral tongue surface NOS |
| B014. | Malignant neoplasm of anterior 2/3 of tongue unspecified |
| B015. | Malignant neoplasm of tongue, junctional zone |
| B016. | Malignant neoplasm of lingual tonsil |
| B017. | Malignant overlapping lesion of tongue |
| B01y. | Malignant neoplasm of other sites of tongue |
| B01z. | Malignant neoplasm of tongue NOS |
| B02.. | Malignant neoplasm of major salivary glands |
| B020. | Malignant neoplasm of parotid gland |
| B021. | Malignant neoplasm of submandibular gland |
| B022. | Malignant neoplasm of sublingual gland |
| B023. | Malignant neoplasm, overlapping lesion of major saliv gland |
| B02y. | Malignant neoplasm of other major salivary glands |
| B02z. | Malignant neoplasm of major salivary gland NOS |
| B03.. | Malignant neoplasm of gum |
| B030. | Malignant neoplasm of upper gum |
| B031. | Malignant neoplasm of lower gum |
| B03y. | Malignant neoplasm of other sites of gum |
| B03z. | Malignant neoplasm of gum NOS |
| B04.. | Malignant neoplasm of floor of mouth |
| B040. | Malignant neoplasm of anterior portion of floor of mouth |
| B041. | Malignant neoplasm of lateral portion of floor of mouth |
| B042. | Malignant neoplasm, overlapping lesion of floor of mouth |
| B04y. | Malignant neoplasm of other sites of floor of mouth |
| B04z. | Malignant neoplasm of floor of mouth NOS |
| B05.. | Malignant neoplasm of other and unspecified parts of mouth |
| B050. | Malignant neoplasm of cheek mucosa |
| B051. | Malignant neoplasm of vestibule of mouth |
| B0510 | Malignant neoplasm of upper buccal sulcus |
| B0511 | Malignant neoplasm of lower buccal sulcus |
| B0512 | Malignant neoplasm of upper labial sulcus |
| B0513 | Malignant neoplasm of lower labial sulcus |
| B051z | Malignant neoplasm of vestibule of mouth NOS |
| B052. | Malignant neoplasm of hard palate |
| B053. | Malignant neoplasm of soft palate |
| B054. | Malignant neoplasm of uvula |
| B055. | Malignant neoplasm of palate unspecified |
| B0550 | Malignant neoplasm of junction of hard and soft palate |
| B0551 | Malignant neoplasm of roof of mouth |
| B055z | Malignant neoplasm of palate NOS |
| B056. | Malignant neoplasm of retromolar area |
| B05y. | Malignant neoplasm of other specified mouth parts |
| B05z. | Malignant neoplasm of mouth NOS |
| B06.. | Malignant neoplasm of oropharynx |
| B060. | Malignant neoplasm of tonsil |
| B0600 | Malignant neoplasm of faucial tonsil |
| B0601 | Malignant neoplasm of palatine tonsil |
| B0602 | Malignant neoplasm of overlapping lesion of tonsil |
| B060z | Malignant neoplasm tonsil NOS |
| B061. | Malignant neoplasm of tonsillar fossa |
| B062. | Malignant neoplasm of tonsillar pillar |
| B0620 | Malignant neoplasm of faucial pillar |
| B0621 | Malignant neoplasm of glossopalatine fold |
| B0622 | Malignant neoplasm of palatoglossal arch |
| B0623 | Malignant neoplasm of palatopharyngeal arch |
| B062z | Malignant neoplasm of tonsillar fossa NOS |
| B063. | Malignant neoplasm of vallecula |
| B064. | Malignant neoplasm of anterior epiglottis |
| B0640 | Malignant neoplasm of epiglottis, free border |
| B0641 | Malignant neoplasm of glossoepiglottic fold |
| B064z | Malignant neoplasm of anterior epiglottis NOS |
| B065. | Malignant neoplasm of junctional region of epiglottis |
| B066. | Malignant neoplasm of lateral wall of oropharynx |
| B067. | Malignant neoplasm of posterior wall of oropharynx |
| B06y. | Malignant neoplasm of oropharynx, other specified sites |
| B06y0 | Malignant neoplasm of branchial cleft |
| B06yz | Malignant neoplasm of other specified site of oropharynx NOS |
| B06z. | Malignant neoplasm of oropharynx NOS |
| B07.. | Malignant neoplasm of nasopharynx |
| B070. | Malignant neoplasm of roof of nasopharynx |
| B071. | Malignant neoplasm of posterior wall of nasopharynx |
| B0710 | Malignant neoplasm of adenoid |
| B0711 | Malignant neoplasm of pharyngeal tonsil |
| B071z | Malignant neoplasm of posterior wall of nasopharynx NOS |
| B072. | Malignant neoplasm of lateral wall of nasopharynx |
| B0720 | Malignant neoplasm of pharyngeal recess |
| B0721 | Malignant neoplasm of opening of auditory tube |
| B072z | Malignant neoplasm of lateral wall of nasopharynx NOS |
| B073. | Malignant neoplasm of anterior wall of nasopharynx |
| B0730 | Malignant neoplasm of floor of nasopharynx |
| B0731 | Malignant neoplasm of nasopharyngeal soft palate surface |
| B0732 | Malignant neoplasm posterior margin nasal septum and choanae |
| B073z | Malignant neoplasm of anterior wall of nasopharynx NOS |
| B074. | Malignant neoplasm, overlapping lesion of nasopharynx |
| B07y. | Malignant neoplasm of other specified site of nasopharynx |
| B07z. | Malignant neoplasm of nasopharynx NOS |
| B08.. | Malignant neoplasm of hypopharynx |
| B080. | Malignant neoplasm of postcricoid region |
| B081. | Malignant neoplasm of pyriform sinus |
| B082. | Malignant neoplasm aryepiglottic fold, hypopharyngeal aspect |
| B083. | Malignant neoplasm of posterior pharynx |
| B084. | Malignant neoplasm, overlapping lesion of hypopharynx |
| B08y. | Malignant neoplasm of other specified hypopharyngeal site |
| B08z. | Malignant neoplasm of hypopharynx NOS |
| B0z.. | Malig neop other/ill-defined sites lip, oral cavity, pharynx |
| B0z0. | Malignant neoplasm of pharynx unspecified |
| B0z1. | Malignant neoplasm of Waldeyer's ring |
| B0z2. | Malignant neoplasm of laryngopharynx |
| B0zy. | Malignant neoplasm of other sites lip, oral cavity, pharynx |
| B0zz. | Malignant neoplasm of lip, oral cavity and pharynx NOS |
| B1... | Malignant neoplasm of digestive organs and peritoneum |
| B10.. | Malignant neoplasm of oesophagus |
| B100. | Malignant neoplasm of cervical oesophagus |
| B101. | Malignant neoplasm of thoracic oesophagus |
| B102. | Malignant neoplasm of abdominal oesophagus |
| B103. | Malignant neoplasm of upper third of oesophagus |
| B104. | Malignant neoplasm of middle third of oesophagus |
| B105. | Malignant neoplasm of lower third of oesophagus |
| B106. | Malignant neoplasm, overlapping lesion of oesophagus |
| B107. | Siewert type I adenocarcinoma |
| B10y. | Malignant neoplasm of other specified part of oesophagus |
| B10z. | Malignant neoplasm of oesophagus NOS |
| B11.. | Malignant neoplasm of stomach |
| B110. | Malignant neoplasm of cardia of stomach |
| B1100 | Malignant neoplasm of cardiac orifice of stomach |
| B1101 | Malignant neoplasm of cardio-oesophageal junction of stomach |
| B110z | Malignant neoplasm of cardia of stomach NOS |
| B111. | Malignant neoplasm of pylorus of stomach |
| B1110 | Malignant neoplasm of prepylorus of stomach |
| B1111 | Malignant neoplasm of pyloric canal of stomach |
| B111z | Malignant neoplasm of pylorus of stomach NOS |
| B112. | Malignant neoplasm of pyloric antrum of stomach |
| B113. | Malignant neoplasm of fundus of stomach |
| B114. | Malignant neoplasm of body of stomach |
| B115. | Malignant neoplasm of lesser curve of stomach unspecified |
| B116. | Malignant neoplasm of greater curve of stomach unspecified |
| B117. | Malignant neoplasm, overlapping lesion of stomach |
| B118. | Siewert type II adenocarcinoma |
| B119. | Siewert type III adenocarcinoma |
| B11y. | Malignant neoplasm of other specified site of stomach |
| B11y0 | Malignant neoplasm of anterior wall of stomach NEC |
| B11y1 | Malignant neoplasm of posterior wall of stomach NEC |
| B11yz | Malignant neoplasm of other specified site of stomach NOS |
| B11z. | Malignant neoplasm of stomach NOS |
| B12.. | Malignant neoplasm of small intestine and duodenum |
| B120. | Malignant neoplasm of duodenum |
| B121. | Malignant neoplasm of jejunum |
| B122. | Malignant neoplasm of ileum |
| B123. | Malignant neoplasm of Meckel's diverticulum |
| B124. | Malignant neoplasm, overlapping lesion of small intestine |
| B12y. | Malignant neoplasm of other specified site small intestine |
| B12z. | Malignant neoplasm of small intestine NOS |
| B13.. | Malignant neoplasm of colon |
| B130. | Malignant neoplasm of hepatic flexure of colon |
| B131. | Malignant neoplasm of transverse colon |
| B132. | Malignant neoplasm of descending colon |
| B133. | Malignant neoplasm of sigmoid colon |
| B134. | Malignant neoplasm of caecum |
| B135. | Malignant neoplasm of appendix |
| B136. | Malignant neoplasm of ascending colon |
| B137. | Malignant neoplasm of splenic flexure of colon |
| B138. | Malignant neoplasm, overlapping lesion of colon |
| B139. | Hereditary nonpolyposis colon cancer |
| B13y. | Malignant neoplasm of other specified sites of colon |
| B13z. | Malignant neoplasm of colon NOS |
| B14.. | Malignant neoplasm of rectum, rectosigmoid junction and anus |
| B140. | Malignant neoplasm of rectosigmoid junction |
| B141. | Malignant neoplasm of rectum |
| B142. | Malignant neoplasm of anal canal |
| B1420 | Malignant neoplasm of cloacogenic zone |
| B143. | Malignant neoplasm of anus unspecified |
| B14y. | Malig neop other site rectum, rectosigmoid junction and anus |
| B14z. | Malignant neoplasm rectum,rectosigmoid junction and anus NOS |
| B15.. | Malignant neoplasm of liver and intrahepatic bile ducts |
| B150. | Primary malignant neoplasm of liver |
| B1500 | Primary carcinoma of liver |
| B1501 | Hepatoblastoma of liver |
| B1502 | Primary angiosarcoma of liver |
| B1503 | Hepatocellular carcinoma |
| B150z | Primary malignant neoplasm of liver NOS |
| B151. | Malignant neoplasm of intrahepatic bile ducts |
| B1510 | Malignant neoplasm of interlobular bile ducts |
| B1511 | Malignant neoplasm of interlobular biliary canals |
| B1512 | Malignant neoplasm of intrahepatic biliary passages |
| B1513 | Malignant neoplasm of intrahepatic canaliculi |
| B1514 | Malignant neoplasm of intrahepatic gall duct |
| B151z | Malignant neoplasm of intrahepatic bile ducts NOS |
| B152. | Malignant neoplasm of liver unspecified |
| B153. | Secondary malignant neoplasm of liver |
| B15z. | Malignant neoplasm of liver and intrahepatic bile ducts NOS |
| B16.. | Malignant neoplasm gallbladder and extrahepatic bile ducts |
| B160. | Malignant neoplasm of gallbladder |
| B161. | Malignant neoplasm of extrahepatic bile ducts |
| B1610 | Malignant neoplasm of cystic duct |
| B1611 | Malignant neoplasm of hepatic duct |
| B1612 | Malignant neoplasm of common bile duct |
| B1613 | Malignant neoplasm of sphincter of Oddi |
| B161z | Malignant neoplasm of extrahepatic bile ducts NOS |
| B162. | Malignant neoplasm of ampulla of Vater |
| B163. | Malignant neoplasm, overlapping lesion of biliary tract |
| B16y. | Malignant neoplasm other gallbladder/extrahepatic bile duct |
| B16z. | Malignant neoplasm gallbladder/extrahepatic bile ducts NOS |
| B17.. | Malignant neoplasm of pancreas |
| B170. | Malignant neoplasm of head of pancreas |
| B171. | Malignant neoplasm of body of pancreas |
| B172. | Malignant neoplasm of tail of pancreas |
| B173. | Malignant neoplasm of pancreatic duct |
| B174. | Malignant neoplasm of Islets of Langerhans |
| B175. | Malignant neoplasm, overlapping lesion of pancreas |
| B176. | Somatostatinoma of pancreas |
| B17y. | Malignant neoplasm of other specified sites of pancreas |
| B17y0 | Malignant neoplasm of ectopic pancreatic tissue |
| B17yz | Malignant neoplasm of specified site of pancreas NOS |
| B17z. | Malignant neoplasm of pancreas NOS |
| B18.. | Malignant neoplasm of retroperitoneum and peritoneum |
| B180. | Malignant neoplasm of retroperitoneum |
| B1800 | Malignant neoplasm of periadrenal tissue |
| B1801 | Malignant neoplasm of perinephric tissue |
| B1802 | Malignant neoplasm of retrocaecal tissue |
| B180z | Malignant neoplasm of retroperitoneum NOS |
| B181. | Mesothelioma of peritoneum |
| B182. | Overlapping malign lesion of retroperitoneum and peritoneum |
| B18y. | Malignant neoplasm of specified parts of peritoneum |
| B18y0 | Malignant neoplasm of mesocolon |
| B18y1 | Malignant neoplasm of mesocaecum |
| B18y2 | Malignant neoplasm of mesorectum |
| B18y3 | Malignant neoplasm of omentum |
| B18y4 | Malignant neoplasm of parietal peritoneum |
| B18y5 | Malignant neoplasm of pelvic peritoneum |
| B18y6 | Malignant neoplasm of the pouch of Douglas |
| B18y7 | Malignant neoplasm of mesentery |
| B18yz | Malignant neoplasm of specified parts of peritoneum NOS |
| B18z. | Malignant neoplasm of retroperitoneum and peritoneum NOS |
| B1z.. | Malig neop oth/ill-defined sites digestive tract/peritoneum |
| B1z0. | Malignant neoplasm of intestinal tract, part unspecified |
| B1z1. | Malignant neoplasm of spleen NEC |
| B1z10 | Angiosarcoma of spleen |
| B1z11 | Fibrosarcoma of spleen |
| B1z1z | Malignant neoplasm of spleen NOS |
| B1z2. | Malignant neoplasm, overlapping lesion of digestive system |
| B1zy. | Malignant neoplasm other spec digestive tract and peritoneum |
| B1zz. | Malignant neoplasm of digestive tract and peritoneum NOS |
| B2... | Malig neop of respiratory tract and intrathoracic organs |
| B20.. | Malig neop nasal cavities, middle ear and accessory sinuses |
| B200. | Malignant neoplasm of nasal cavities |
| B2000 | Malignant neoplasm of cartilage of nose |
| B2001 | Malignant neoplasm of nasal conchae |
| B2002 | Malignant neoplasm of septum of nose |
| B2003 | Malignant neoplasm of vestibule of nose |
| B200z | Malignant neoplasm of nasal cavities NOS |
| B201. | Malig neop auditory tube, middle ear and mastoid air cells |
| B2010 | Malignant neoplasm of auditory (Eustachian) tube |
| B2011 | Malignant neoplasm of tympanic cavity |
| B2012 | Malignant neoplasm of tympanic antrum |
| B2013 | Malignant neoplasm of mastoid air cells |
| B201z | Malig neop auditory tube, middle ear, mastoid air cells NOS |
| B202. | Malignant neoplasm of maxillary sinus |
| B203. | Malignant neoplasm of ethmoid sinus |
| B204. | Malignant neoplasm of frontal sinus |
| B205. | Malignant neoplasm of sphenoidal sinus |
| B206. | Malignant neoplasm, overlapping lesion of accessory sinuses |
| B20y. | Malig neop other site nasal cavity, middle ear and sinuses |
| B20z. | Malignant neoplasm of accessory sinus NOS |
| B21.. | Malignant neoplasm of larynx |
| B210. | Malignant neoplasm of glottis |
| B211. | Malignant neoplasm of supraglottis |
| B212. | Malignant neoplasm of subglottis |
| B213. | Malignant neoplasm of laryngeal cartilage |
| B2130 | Malignant neoplasm of arytenoid cartilage |
| B2131 | Malignant neoplasm of cricoid cartilage |
| B2132 | Malignant neoplasm of cuneiform cartilage |
| B2133 | Malignant neoplasm of thyroid cartilage |
| B213z | Malignant neoplasm of laryngeal cartilage NOS |
| B214. | Malignant neoplasm, overlapping lesion of larynx |
| B215. | Malignant neoplasm of epiglottis NOS |
| B21y. | Malignant neoplasm of larynx, other specified site |
| B21z. | Malignant neoplasm of larynx NOS |
| B22.. | Malignant neoplasm of trachea, bronchus and lung |
| B220. | Malignant neoplasm of trachea |
| B2200 | Malignant neoplasm of cartilage of trachea |
| B2201 | Malignant neoplasm of mucosa of trachea |
| B220z | Malignant neoplasm of trachea NOS |
| B221. | Malignant neoplasm of main bronchus |
| B2210 | Malignant neoplasm of carina of bronchus |
| B2211 | Malignant neoplasm of hilus of lung |
| B221z | Malignant neoplasm of main bronchus NOS |
| B222. | Malignant neoplasm of upper lobe, bronchus or lung |
| B2220 | Malignant neoplasm of upper lobe bronchus |
| B2221 | Malignant neoplasm of upper lobe of lung |
| B222z | Malignant neoplasm of upper lobe, bronchus or lung NOS |
| B223. | Malignant neoplasm of middle lobe, bronchus or lung |
| B2230 | Malignant neoplasm of middle lobe bronchus |
| B2231 | Malignant neoplasm of middle lobe of lung |
| B223z | Malignant neoplasm of middle lobe, bronchus or lung NOS |
| B224. | Malignant neoplasm of lower lobe, bronchus or lung |
| B2240 | Malignant neoplasm of lower lobe bronchus |
| B2241 | Malignant neoplasm of lower lobe of lung |
| B224z | Malignant neoplasm of lower lobe, bronchus or lung NOS |
| B225. | Malignant neoplasm of overlapping lesion of bronchus & lung |
| B226. | Mesothelioma |
| B22y. | Malignant neoplasm of other sites of bronchus or lung |
| B22z. | Malignant neoplasm of bronchus or lung NOS |
| B23.. | Malignant neoplasm of pleura |
| B230. | Malignant neoplasm of parietal pleura |
| B231. | Malignant neoplasm of visceral pleura |
| B232. | Mesothelioma of pleura |
| B23y. | Malignant neoplasm of other specified pleura |
| B23z. | Malignant neoplasm of pleura NOS |
| B24.. | Malignant neoplasm of thymus, heart and mediastinum |
| B240. | Malignant neoplasm of thymus |
| B241. | Malignant neoplasm of heart |
| B2410 | Malignant neoplasm of endocardium |
| B2411 | Malignant neoplasm of epicardium |
| B2412 | Malignant neoplasm of myocardium |
| B2413 | Malignant neoplasm of pericardium |
| B2414 | Mesothelioma of pericardium |
| B241z | Malignant neoplasm of heart NOS |
| B242. | Malignant neoplasm of anterior mediastinum |
| B243. | Malignant neoplasm of posterior mediastinum |
| B24X. | Malignant neoplasm of mediastinum, part unspecified |
| B24y. | Malig neop of other site of heart, thymus and mediastinum |
| B24z. | Malignant neoplasm of heart, thymus and mediastinum NOS |
| B25.. | Malig neo, overlapping lesion of heart, mediastinum & pleura |
| B26.. | Malignant neoplasm, overlap lesion of resp & intrathor orgs |
| B2z.. | Malig neop other/ill-defined sites resp/intrathoracic organs |
| B2z0. | Malig neop of upper respiratory tract, part unspecified |
| B2zy. | Malignant neoplasm of other site of respiratory tract |
| B2zz. | Malignant neoplasm of respiratory tract NOS |
| B3... | Malig neop of bone, connective tissue, skin and breast |
| B30.. | Malignant neoplasm of bone and articular cartilage |
| B300. | Malignant neoplasm of bones of skull and face |
| B3000 | Malignant neoplasm of ethmoid bone |
| B3001 | Malignant neoplasm of frontal bone |
| B3002 | Malignant neoplasm of malar bone |
| B3003 | Malignant neoplasm of nasal bone |
| B3004 | Malignant neoplasm of occipital bone |
| B3005 | Malignant neoplasm of orbital bone |
| B3006 | Malignant neoplasm of parietal bone |
| B3007 | Malignant neoplasm of sphenoid bone |
| B3008 | Malignant neoplasm of temporal bone |
| B3009 | Malignant neoplasm of zygomatic bone |
| B300A | Malignant neoplasm of maxilla |
| B300B | Malignant neoplasm of turbinate |
| B300C | Malignant neoplasm of vomer |
| B300z | Malignant neoplasm of bones of skull and face NOS |
| B301. | Malignant neoplasm of mandible |
| B302. | Malignant neoplasm of vertebral column |
| B3020 | Malignant neoplasm of cervical vertebra |
| B3021 | Malignant neoplasm of thoracic vertebra |
| B3022 | Malignant neoplasm of lumbar vertebra |
| B302z | Malignant neoplasm of vertebral column NOS |
| B303. | Malignant neoplasm of ribs, sternum and clavicle |
| B3030 | Malignant neoplasm of rib |
| B3031 | Malignant neoplasm of sternum |
| B3032 | Malignant neoplasm of clavicle |
| B3033 | Malignant neoplasm of costal cartilage |
| B3034 | Malignant neoplasm of costo-vertebral joint |
| B3035 | Malignant neoplasm of xiphoid process |
| B303z | Malignant neoplasm of rib, sternum and clavicle NOS |
| B304. | Malignant neoplasm of scapula and long bones of upper arm |
| B3040 | Malignant neoplasm of scapula |
| B3041 | Malignant neoplasm of acromion |
| B3042 | Malignant neoplasm of humerus |
| B3043 | Malignant neoplasm of radius |
| B3044 | Malignant neoplasm of ulna |
| B304z | Malig neop of scapula and long bones of upper arm NOS |
| B305. | Malignant neoplasm of hand bones |
| B3050 | Malignant neoplasm of carpal bone - scaphoid |
| B3051 | Malignant neoplasm of carpal bone - lunate |
| B3052 | Malignant neoplasm of carpal bone - triquetrum |
| B3053 | Malignant neoplasm of carpal bone - pisiform |
| B3054 | Malignant neoplasm of carpal bone - trapezium |
| B3055 | Malignant neoplasm of carpal bone - trapezoid |
| B3056 | Malignant neoplasm of carpal bone - capitate |
| B3057 | Malignant neoplasm of carpal bone - hamate |
| B3058 | Malignant neoplasm of first metacarpal bone |
| B3059 | Malignant neoplasm of second metacarpal bone |
| B305A | Malignant neoplasm of third metacarpal bone |
| B305B | Malignant neoplasm of fourth metacarpal bone |
| B305C | Malignant neoplasm of fifth metacarpal bone |
| B305D | Malignant neoplasm of phalanges of hand |
| B305z | Malignant neoplasm of hand bones NOS |
| B306. | Malignant neoplasm of pelvic bones, sacrum and coccyx |
| B3060 | Malignant neoplasm of ilium |
| B3061 | Malignant neoplasm of ischium |
| B3062 | Malignant neoplasm of pubis |
| B3063 | Malignant neoplasm of sacral vertebra |
| B3064 | Malignant neoplasm of coccygeal vertebra |
| B3065 | Malignant sacral teratoma |
| B306z | Malignant neoplasm of pelvis, sacrum or coccyx NOS |
| B307. | Malignant neoplasm of long bones of leg |
| B3070 | Malignant neoplasm of femur |
| B3071 | Malignant neoplasm of fibula |
| B3072 | Malignant neoplasm of tibia |
| B307z | Malignant neoplasm of long bones of leg NOS |
| B308. | Malignant neoplasm of short bones of leg |
| B3080 | Malignant neoplasm of patella |
| B3081 | Malignant neoplasm of talus |
| B3082 | Malignant neoplasm of calcaneum |
| B3083 | Malignant neoplasm of medial cuneiform |
| B3084 | Malignant neoplasm of intermediate cuneiform |
| B3085 | Malignant neoplasm of lateral cuneiform |
| B3086 | Malignant neoplasm of cuboid |
| B3087 | Malignant neoplasm of navicular |
| B3088 | Malignant neoplasm of first metatarsal bone |
| B3089 | Malignant neoplasm of second metatarsal bone |
| B308A | Malignant neoplasm of third metatarsal bone |
| B308B | Malignant neoplasm of fourth metatarsal bone |
| B308C | Malignant neoplasm of fifth metatarsal bone |
| B308D | Malignant neoplasm of phalanges of foot |
| B308z | Malignant neoplasm of short bones of leg NOS |
| B309. | Malignant neoplasm, overlap les bone and artic cart of limbs |
| B30W. | Malignant neoplasm/overlap lesion/bone+articulr cartilage |
| B30X. | Malignant neoplasm/bones+articular cartilage/limb,unspfd |
| B30z. | Malignant neoplasm of bone and articular cartilage NOS |
| B30z0 | Osteosarcoma |
| B31.. | Malignant neoplasm of connective and other soft tissue |
| B310. | Malig neop of connective and soft tissue head, face and neck |
| B3100 | Malignant neoplasm of soft tissue of head |
| B3101 | Malignant neoplasm of soft tissue of face |
| B3102 | Malignant neoplasm of soft tissue of neck |
| B3103 | Malignant neoplasm of cartilage of ear |
| B3104 | Malignant neoplasm of tarsus of eyelid |
| B3105 | Malignant neoplasm soft tissues of cervical spine |
| B310z | Malig neop connective and soft tissue head, face, neck NOS |
| B311. | Malig neop connective and soft tissue upper limb/shoulder |
| B3110 | Malignant neoplasm of connective and soft tissue of shoulder |
| B3111 | Malignant neoplasm of connective and soft tissue, upper arm |
| B3112 | Malignant neoplasm of connective and soft tissue of fore-arm |
| B3113 | Malignant neoplasm of connective and soft tissue of hand |
| B3114 | Malignant neoplasm of connective and soft tissue of finger |
| B3115 | Malignant neoplasm of connective and soft tissue of thumb |
| B311z | Malig neop connective soft tissue upper limb/shoulder NOS |
| B312. | Malig neop of connective and soft tissue of hip and leg |
| B3120 | Malignant neoplasm of connective and soft tissue of hip |
| B3121 | Malig neop of connective and soft tissue thigh and upper leg |
| B3122 | Malig neop connective and soft tissue of popliteal space |
| B3123 | Malig neop of connective and soft tissue of lower leg |
| B3124 | Malignant neoplasm of connective and soft tissue of foot |
| B3125 | Malignant neoplasm of connective and soft tissue of toe |
| B3126 | Malig neop of connective and soft tissue of great toe |
| B312z | Malig neop connective and soft tissue hip and leg NOS |
| B313. | Malignant neoplasm of connective and soft tissue of thorax |
| B3130 | Malignant neoplasm of connective and soft tissue of axilla |
| B3131 | Malignant neoplasm of diaphragm |
| B3132 | Malignant neoplasm of great vessels |
| B3133 | Malig neoplasm of connective and soft tissues of thor spine |
| B313z | Malig neop of connective and soft tissue of thorax NOS |
| B314. | Malignant neoplasm of connective and soft tissue of abdomen |
| B3140 | Malig neop of connective and soft tissue of abdominal wall |
| B3141 | Malig neoplasm of connective and soft tissues of lumb spine |
| B314z | Malig neop of connective and soft tissue of abdomen NOS |
| B315. | Malignant neoplasm of connective and soft tissue of pelvis |
| B3150 | Malignant neoplasm of connective and soft tissue of buttock |
| B3151 | Malig neop of connective and soft tissue of inguinal region |
| B3152 | Malignant neoplasm of connective and soft tissue of perineum |
| B3153 | Malig neopl of connective and soft tissue - sacrum or coccyx |
| B315z | Malig neop of connective and soft tissue of pelvis NOS |
| B316. | Malig neop of connective and soft tissue trunk unspecified |
| B317. | Malignant neoplasm, overlap lesion connective & soft tissue |
| B31y. | Malig neop connective and soft tissue other specified site |
| B31z. | Malignant neoplasm of connective and soft tissue, site NOS |
| B31z0 | Kaposi's sarcoma of soft tissue |
| B32.. | Malignant melanoma of skin |
| B320. | Malignant melanoma of lip |
| B321. | Malignant melanoma of eyelid including canthus |
| B322. | Malignant melanoma of ear and external auricular canal |
| B3220 | Malignant melanoma of auricle (ear) |
| B3221 | Malignant melanoma of external auditory meatus |
| B322z | Malignant melanoma of ear and external auricular canal NOS |
| B323. | Malignant melanoma of other and unspecified parts of face |
| B3230 | Malignant melanoma of external surface of cheek |
| B3231 | Malignant melanoma of chin |
| B3232 | Malignant melanoma of eyebrow |
| B3233 | Malignant melanoma of forehead |
| B3234 | Malignant melanoma of external surface of nose |
| B3235 | Malignant melanoma of temple |
| B323z | Malignant melanoma of face NOS |
| B324. | Malignant melanoma of scalp and neck |
| B3240 | Malignant melanoma of scalp |
| B3241 | Malignant melanoma of neck |
| B324z | Malignant melanoma of scalp and neck NOS |
| B325. | Malignant melanoma of trunk (excluding scrotum) |
| B3250 | Malignant melanoma of axilla |
| B3251 | Malignant melanoma of breast |
| B3252 | Malignant melanoma of buttock |
| B3253 | Malignant melanoma of groin |
| B3254 | Malignant melanoma of perianal skin |
| B3255 | Malignant melanoma of perineum |
| B3256 | Malignant melanoma of umbilicus |
| B3257 | Malignant melanoma of back |
| B3258 | Malignant melanoma of chest wall |
| B325z | Malignant melanoma of trunk, excluding scrotum, NOS |
| B326. | Malignant melanoma of upper limb and shoulder |
| B3260 | Malignant melanoma of shoulder |
| B3261 | Malignant melanoma of upper arm |
| B3262 | Malignant melanoma of fore-arm |
| B3263 | Malignant melanoma of hand |
| B3264 | Malignant melanoma of finger |
| B3265 | Malignant melanoma of thumb |
| B326z | Malignant melanoma of upper limb or shoulder NOS |
| B327. | Malignant melanoma of lower limb and hip |
| B3270 | Malignant melanoma of hip |
| B3271 | Malignant melanoma of thigh |
| B3272 | Malignant melanoma of knee |
| B3273 | Malignant melanoma of popliteal fossa area |
| B3274 | Malignant melanoma of lower leg |
| B3275 | Malignant melanoma of ankle |
| B3276 | Malignant melanoma of heel |
| B3277 | Malignant melanoma of foot |
| B3278 | Malignant melanoma of toe |
| B3279 | Malignant melanoma of great toe |
| B327z | Malignant melanoma of lower limb or hip NOS |
| B32y. | Malignant melanoma of other specified skin site |
| B32y0 | Overlapping malignant melanoma of skin |
| B32z. | Malignant melanoma of skin NOS |
| B33.. | Other malignant neoplasm of skin |
| B330. | Malignant neoplasm of skin of lip |
| B331. | Malignant neoplasm of eyelid including canthus |
| B3310 | Malignant neoplasm of canthus |
| B3311 | Malignant neoplasm of upper eyelid |
| B3312 | Malignant neoplasm of lower eyelid |
| B332. | Malignant neoplasm skin of ear and external auricular canal |
| B3320 | Malignant neoplasm of skin of auricle (ear) |
| B3321 | Malignant neoplasm of skin of external auditory meatus |
| B3322 | Malignant neoplasm of pinna NEC |
| B332z | Malig neop skin of ear and external auricular canal NOS |
| B333. | Malignant neoplasm skin of other and unspecified parts face |
| B3330 | Malignant neoplasm of skin of cheek, external |
| B3331 | Malignant neoplasm of skin of chin |
| B3332 | Malignant neoplasm of skin of eyebrow |
| B3333 | Malignant neoplasm of skin of forehead |
| B3334 | Malignant neoplasm of skin of nose (external) |
| B3335 | Malignant neoplasm of skin of temple |
| B333z | Malignant neoplasm skin other and unspec part of face NOS |
| B334. | Malignant neoplasm of scalp and skin of neck |
| B3340 | Malignant neoplasm of scalp |
| B3341 | Malignant neoplasm of skin of neck |
| B334z | Malignant neoplasm of scalp or skin of neck NOS |
| B335. | Malignant neoplasm of skin of trunk, excluding scrotum |
| B3350 | Malignant neoplasm of skin of axillary fold |
| B3351 | Malignant neoplasm of skin of chest, excluding breast |
| B3352 | Malignant neoplasm of skin of breast |
| B3353 | Malignant neoplasm of skin of abdominal wall |
| B3354 | Malignant neoplasm of skin of umbilicus |
| B3355 | Malignant neoplasm of skin of groin |
| B3356 | Malignant neoplasm of skin of perineum |
| B3357 | Malignant neoplasm of skin of back |
| B3358 | Malignant neoplasm of skin of buttock |
| B3359 | Malignant neoplasm of perianal skin |
| B335A | Malignant neoplasm of skin of scapular region |
| B335z | Malignant neoplasm of skin of trunk, excluding scrotum, NOS |
| B336. | Malignant neoplasm of skin of upper limb and shoulder |
| B3360 | Malignant neoplasm of skin of shoulder |
| B3361 | Malignant neoplasm of skin of upper arm |
| B3362 | Malignant neoplasm of skin of fore-arm |
| B3363 | Malignant neoplasm of skin of hand |
| B3364 | Malignant neoplasm of skin of finger |
| B3365 | Malignant neoplasm of skin of thumb |
| B336z | Malignant neoplasm of skin of upper limb or shoulder NOS |
| B337. | Malignant neoplasm of skin of lower limb and hip |
| B3370 | Malignant neoplasm of skin of hip |
| B3371 | Malignant neoplasm of skin of thigh |
| B3372 | Malignant neoplasm of skin of knee |
| B3373 | Malignant neoplasm of skin of popliteal fossa area |
| B3374 | Malignant neoplasm of skin of lower leg |
| B3375 | Malignant neoplasm of skin of ankle |
| B3376 | Malignant neoplasm of skin of heel |
| B3377 | Malignant neoplasm of skin of foot |
| B3378 | Malignant neoplasm of skin of toe |
| B3379 | Malignant neoplasm of skin of great toe |
| B337z | Malignant neoplasm of skin of lower limb or hip NOS |
| B338. | Squamous cell carcinoma of skin |
| B339. | Dermatofibrosarcoma protuberans |
| B33X. | Malignant neoplasm overlapping lesion of skin |
| B33y. | Malignant neoplasm of other specified skin sites |
| B33z. | Malignant neoplasm of skin NOS |
| B33z0 | Kaposi's sarcoma of skin |
| B33z1 | Naevoid basal cell carcinoma syndrome |
| B34.. | Malignant neoplasm of female breast |
| B340. | Malignant neoplasm of nipple and areola of female breast |
| B3400 | Malignant neoplasm of nipple of female breast |
| B3401 | Malignant neoplasm of areola of female breast |
| B340z | Malignant neoplasm of nipple or areola of female breast NOS |
| B341. | Malignant neoplasm of central part of female breast |
| B342. | Malignant neoplasm of upper-inner quadrant of female breast |
| B343. | Malignant neoplasm of lower-inner quadrant of female breast |
| B344. | Malignant neoplasm of upper-outer quadrant of female breast |
| B345. | Malignant neoplasm of lower-outer quadrant of female breast |
| B346. | Malignant neoplasm of axillary tail of female breast |
| B347. | Malignant neoplasm, overlapping lesion of breast |
| B34y. | Malignant neoplasm of other site of female breast |
| B34y0 | Malignant neoplasm of ectopic site of female breast |
| B34yz | Malignant neoplasm of other site of female breast NOS |
| B34z. | Malignant neoplasm of female breast NOS |
| B35.. | Malignant neoplasm of male breast |
| B350. | Malignant neoplasm of nipple and areola of male breast |
| B3500 | Malignant neoplasm of nipple of male breast |
| B3501 | Malignant neoplasm of areola of male breast |
| B350z | Malignant neoplasm of nipple or areola of male breast NOS |
| B35z. | Malignant neoplasm of other site of male breast |
| B35z0 | Malignant neoplasm of ectopic site of male breast |
| B35zz | Malignant neoplasm of male breast NOS |
| B3y.. | Malig neop of bone, connective tissue, skin and breast OS |
| B3z.. | Malig neop of bone, connective tissue, skin and breast NOS |
| B4... | Malignant neoplasm of genitourinary organ |
| B40.. | Malignant neoplasm of uterus, part unspecified |
| B41.. | Malignant neoplasm of cervix uteri |
| B410. | Malignant neoplasm of endocervix |
| B4100 | Malignant neoplasm of endocervical canal |
| B4101 | Malignant neoplasm of endocervical gland |
| B410z | Malignant neoplasm of endocervix NOS |
| B411. | Malignant neoplasm of exocervix |
| B412. | Malignant neoplasm, overlapping lesion of cervix uteri |
| B41y. | Malignant neoplasm of other site of cervix |
| B41y0 | Malignant neoplasm of cervical stump |
| B41y1 | Malignant neoplasm of squamocolumnar junction of cervix |
| B41yz | Malignant neoplasm of other site of cervix NOS |
| B41z. | Malignant neoplasm of cervix uteri NOS |
| B42.. | Malignant neoplasm of placenta |
| B420. | Choriocarcinoma |
| B43.. | Malignant neoplasm of body of uterus |
| B430. | Malignant neoplasm of corpus uteri, excluding isthmus |
| B4300 | Malignant neoplasm of cornu of corpus uteri |
| B4301 | Malignant neoplasm of fundus of corpus uteri |
| B4302 | Malignant neoplasm of endometrium of corpus uteri |
| B4303 | Malignant neoplasm of myometrium of corpus uteri |
| B430z | Malignant neoplasm of corpus uteri NOS |
| B431. | Malignant neoplasm of isthmus of uterine body |
| B4310 | Malignant neoplasm of lower uterine segment |
| B431z | Malignant neoplasm of isthmus of uterine body NOS |
| B432. | Malignant neoplasm of overlapping lesion of corpus uteri |
| B43y. | Malignant neoplasm of other site of uterine body |
| B43z. | Malignant neoplasm of body of uterus NOS |
| B44.. | Malignant neoplasm of ovary and other uterine adnexa |
| B440. | Malignant neoplasm of ovary |
| B441. | Malignant neoplasm of fallopian tube |
| B442. | Malignant neoplasm of broad ligament |
| B443. | Malignant neoplasm of parametrium |
| B444. | Malignant neoplasm of round ligament |
| B44y. | Malignant neoplasm of other site of uterine adnexa |
| B44z. | Malignant neoplasm of uterine adnexa NOS |
| B45.. | Malig neop of other and unspecified female genital organs |
| B450. | Malignant neoplasm of vagina |
| B4500 | Malignant neoplasm of Gartner's duct |
| B4501 | Malignant neoplasm of vaginal vault |
| B450z | Malignant neoplasm of vagina NOS |
| B451. | Malignant neoplasm of labia majora |
| B4510 | Malignant neoplasm of greater vestibular (Bartholin's) gland |
| B451z | Malignant neoplasm of labia majora NOS |
| B452. | Malignant neoplasm of labia minora |
| B453. | Malignant neoplasm of clitoris |
| B454. | Malignant neoplasm of vulva unspecified |
| B45X. | Malignant neoplasm/overlapping lesion/feml genital organs |
| B45y. | Malignant neoplasm of other specified female genital organ |
| B45y0 | Malignant neoplasm of overlapping lesion of vulva |
| B45z. | Malignant neoplasm of female genital organ NOS |
| B46.. | Malignant neoplasm of prostate |
| B47.. | Malignant neoplasm of testis |
| B470. | Malignant neoplasm of undescended testis |
| B4700 | Malignant neoplasm of ectopic testis |
| B4701 | Malignant neoplasm of retained testis |
| B4702 | Seminoma of undescended testis |
| B4703 | Teratoma of undescended testis |
| B470z | Malignant neoplasm of undescended testis NOS |
| B471. | Malignant neoplasm of descended testis |
| B4710 | Seminoma of descended testis |
| B4711 | Teratoma of descended testis |
| B471z | Malignant neoplasm of descended testis NOS |
| B47z. | Malignant neoplasm of testis NOS |
| B48.. | Malignant neoplasm of penis and other male genital organs |
| B480. | Malignant neoplasm of prepuce (foreskin) |
| B481. | Malignant neoplasm of glans penis |
| B482. | Malignant neoplasm of body of penis |
| B483. | Malignant neoplasm of penis, part unspecified |
| B484. | Malignant neoplasm of epididymis |
| B485. | Malignant neoplasm of spermatic cord |
| B486. | Malignant neoplasm of scrotum |
| B487. | Malignant neoplasm, overlapping lesion of penis |
| B48y. | Malignant neoplasm of other male genital organ |
| B48y0 | Malignant neoplasm of seminal vesicle |
| B48y1 | Malignant neoplasm of tunica vaginalis |
| B48y2 | Malignant neoplasm, overlapping lesion male genital orgs |
| B48yz | Malignant neoplasm of other male genital organ NOS |
| B48z. | Malignant neoplasm of penis and other male genital organ NOS |
| B49.. | Malignant neoplasm of urinary bladder |
| B490. | Malignant neoplasm of trigone of urinary bladder |
| B491. | Malignant neoplasm of dome of urinary bladder |
| B492. | Malignant neoplasm of lateral wall of urinary bladder |
| B493. | Malignant neoplasm of anterior wall of urinary bladder |
| B494. | Malignant neoplasm of posterior wall of urinary bladder |
| B495. | Malignant neoplasm of bladder neck |
| B496. | Malignant neoplasm of ureteric orifice |
| B497. | Malignant neoplasm of urachus |
| B49y. | Malignant neoplasm of other site of urinary bladder |
| B49y0 | Malignant neoplasm, overlapping lesion of bladder |
| B49z. | Malignant neoplasm of urinary bladder NOS |
| B4A.. | Malig neop of kidney and other unspecified urinary organs |
| B4A0. | Malignant neoplasm of kidney parenchyma |
| B4A00 | Hypernephroma |
| B4A1. | Malignant neoplasm of renal pelvis |
| B4A10 | Malignant neoplasm of renal calyces |
| B4A11 | Malignant neoplasm of ureteropelvic junction |
| B4A1z | Malignant neoplasm of renal pelvis NOS |
| B4A2. | Malignant neoplasm of ureter |
| B4A3. | Malignant neoplasm of urethra |
| B4A4. | Malignant neoplasm of paraurethral glands |
| B4Ay. | Malignant neoplasm of other urinary organs |
| B4Ay0 | Malignant neoplasm of overlapping lesion of urinary organs |
| B4Az. | Malignant neoplasm of kidney or urinary organs NOS |
| B4y.. | Malignant neoplasm of genitourinary organ OS |
| B4z.. | Malignant neoplasm of genitourinary organ NOS |
| B5... | Malignant neoplasm of other and unspecified sites |
| B50.. | Malignant neoplasm of eye |
| B500. | Malig neop eyeball excl conjunctiva, cornea, retina, choroid |
| B5000 | Malignant neoplasm of ciliary body |
| B5001 | Malignant neoplasm of iris |
| B5002 | Malignant neoplasm of crystalline lens |
| B5003 | Malignant neoplasm of sclera |
| B500z | Malignant neoplasm of eyeball NOS |
| B501. | Malignant neoplasm of orbit |
| B5010 | Malignant neoplasm of connective tissue of orbit |
| B5011 | Malignant neoplasm of extraocular muscle of orbit |
| B501z | Malignant neoplasm of orbit NOS |
| B502. | Malignant neoplasm of lacrimal gland |
| B503. | Malignant neoplasm of conjunctiva |
| B504. | Malignant neoplasm of cornea |
| B505. | Malignant neoplasm of retina |
| B506. | Malignant neoplasm of choroid |
| B507. | Malignant neoplasm of lacrimal duct |
| B5070 | Malignant neoplasm of lacrimal sac |
| B5071 | Malignant neoplasm of nasolacrimal duct |
| B507z | Malignant neoplasm of lacrimal duct NOS |
| B508. | Malignant neoplasm, overlapping lesion of eye and adnexa |
| B50y. | Malignant neoplasm of other specified site of eye |
| B50z. | Malignant neoplasm of eye NOS |
| B51.. | Malignant neoplasm of brain |
| B510. | Malignant neoplasm cerebrum (excluding lobes and ventricles) |
| B5100 | Malignant neoplasm of basal ganglia |
| B5101 | Malignant neoplasm of cerebral cortex |
| B5102 | Malignant neoplasm of corpus striatum |
| B5103 | Malignant neoplasm of globus pallidus |
| B5104 | Malignant neoplasm of hypothalamus |
| B5105 | Malignant neoplasm of thalamus |
| B510z | Malignant neoplasm of cerebrum NOS |
| B511. | Malignant neoplasm of frontal lobe |
| B512. | Malignant neoplasm of temporal lobe |
| B5120 | Malignant neoplasm of hippocampus |
| B5121 | Malignant neoplasm of uncus |
| B512z | Malignant neoplasm of temporal lobe NOS |
| B513. | Malignant neoplasm of parietal lobe |
| B514. | Malignant neoplasm of occipital lobe |
| B515. | Malignant neoplasm of cerebral ventricles |
| B5150 | Malignant neoplasm of choroid plexus |
| B5151 | Malignant neoplasm of floor of cerebral ventricle |
| B515z | Malignant neoplasm of cerebral ventricle NOS |
| B516. | Malignant neoplasm of cerebellum |
| B517. | Malignant neoplasm of brain stem |
| B5170 | Malignant neoplasm of cerebral peduncle |
| B5171 | Malignant neoplasm of medulla oblongata |
| B5172 | Malignant neoplasm of midbrain |
| B5173 | Malignant neoplasm of pons |
| B517z | Malignant neoplasm of brain stem NOS |
| B51y. | Malignant neoplasm of other parts of brain |
| B51y0 | Malignant neoplasm of corpus callosum |
| B51y1 | Malignant neoplasm of tapetum |
| B51y2 | Malignant neoplasm, overlapping lesion of brain |
| B51yz | Malignant neoplasm of other part of brain NOS |
| B51z. | Malignant neoplasm of brain NOS |
| B52.. | Malig neop of other and unspecified parts of nervous system |
| B520. | Malignant neoplasm of cranial nerves |
| B5200 | Malignant neoplasm of olfactory bulb |
| B5201 | Malignant neoplasm of optic nerve |
| B5202 | Malignant neoplasm of acoustic nerve |
| B520z | Malignant neoplasm of cranial nerves NOS |
| B521. | Malignant neoplasm of cerebral meninges |
| B5210 | Malignant neoplasm of cerebral dura mater |
| B5211 | Malignant neoplasm of cerebral arachnoid mater |
| B5212 | Malignant neoplasm of cerebral pia mater |
| B521z | Malignant neoplasm of cerebral meninges NOS |
| B522. | Malignant neoplasm of spinal cord |
| B523. | Malignant neoplasm of spinal meninges |
| B5230 | Malignant neoplasm of spinal dura mater |
| B5231 | Malignant neoplasm of spinal arachnoid mater |
| B5232 | Malignant neoplasm of spinal pia mater |
| B523z | Malignant neoplasm of spinal meninges NOS |
| B524. | Malig neopl peripheral nerves and autonomic nervous system |
| B5240 | Malignant neoplasm of peripheral nerves of head, face & neck |
| B5241 | Malignant neoplasm of peripheral nerve,upp limb,incl should |
| B5242 | Malignant neoplasm of peripheral nerve of low limb, incl hip |
| B5243 | Malignant neoplasm of peripheral nerve of thorax |
| B5244 | Malignant neoplasm of peripheral nerve of abdomen |
| B5245 | Malignant neoplasm of peripheral nerve of pelvis |
| B5246 | Malignant neoplasm,overlap lesion periph nerve & auton ns |
| B524W | Mal neoplasm/periph nerves+autonomic nervous system,unspc |
| B524X | Malignant neoplasm/peripheral nerves of trunk,unspecified |
| B525. | Malignant neoplasm of cauda equina |
| B52W. | Malig neopl, overlap lesion brain & other part of CNS |
| B52X. | Malignant neoplasm of meninges, unspecified |
| B52y. | Malignant neoplasm of other specified part of nervous system |
| B52z. | Malignant neoplasm of nervous system NOS |
| B53.. | Malignant neoplasm of thyroid gland |
| B54.. | Malig neop of other endocrine glands and related structures |
| B540. | Malignant neoplasm of adrenal gland |
| B5400 | Malignant neoplasm of adrenal cortex |
| B5401 | Malignant neoplasm of adrenal medulla |
| B540z | Malignant neoplasm of adrenal gland NOS |
| B541. | Malignant neoplasm of parathyroid gland |
| B542. | Malignant neoplasm pituitary gland and craniopharyngeal duct |
| B5420 | Malignant neoplasm of pituitary gland |
| B5421 | Malignant neoplasm of craniopharyngeal duct |
| B542z | Malig neop pituitary gland or craniopharyngeal duct NOS |
| B543. | Malignant neoplasm of pineal gland |
| B544. | Malignant neoplasm of carotid body |
| B545. | Malignant neoplasm of aortic body and other paraganglia |
| B5450 | Malignant neoplasm of glomus jugulare |
| B5451 | Malignant neoplasm of aortic body |
| B5452 | Malignant neoplasm of coccygeal body |
| B545z | Malignant neoplasm of aortic body or paraganglia NOS |
| B546. | Neuroblastoma |
| B54X. | Malignant neoplasm-pluriglandular involvement,unspecified |
| B54y. | Malignant neoplasm of other specified endocrine gland |
| B54z. | Malig neop of endocrine gland or related structure NOS |
| B55.. | Malignant neoplasm of other and ill-defined sites |
| B550. | Malignant neoplasm of head, neck and face |
| B5500 | Malignant neoplasm of head NOS |
| B5501 | Malignant neoplasm of cheek NOS |
| B5502 | Malignant neoplasm of nose NOS |
| B5503 | Malignant neoplasm of jaw NOS |
| B5504 | Malignant neoplasm of neck NOS |
| B5505 | Malignant neoplasm of supraclavicular fossa NOS |
| B550z | Malignant neoplasm of head, neck and face NOS |
| B551. | Malignant neoplasm of thorax |
| B5510 | Malignant neoplasm of axilla NOS |
| B5511 | Malignant neoplasm of chest wall NOS |
| B5512 | Malignant neoplasm of intrathoracic site NOS |
| B551z | Malignant neoplasm of thorax NOS |
| B552. | Malignant neoplasm of abdomen |
| B553. | Malignant neoplasm of pelvis |
| B5530 | Malignant neoplasm of inguinal region NOS |
| B5531 | Malignant neoplasm of presacral region |
| B5532 | Malignant neoplasm of sacrococcygeal region |
| B553z | Malignant neoplasm of pelvis NOS |
| B554. | Malignant neoplasm of upper limb NOS |
| B555. | Malignant neoplasm of lower limb NOS |
| B55y. | Malignant neoplasm of other specified sites |
| B55y0 | Malignant neoplasm of back NOS |
| B55y1 | Malignant neoplasm of trunk NOS |
| B55y2 | Malignant neoplasm of flank NOS |
| B55yz | Malignant neoplasm of specified site NOS |
| B55z. | Malignant neoplasm of other and ill defined site NOS |
| B56.. | Secondary and unspecified malignant neoplasm of lymph nodes |
| B560. | Secondary and unspec malig neop lymph nodes head/face/neck |
| B5600 | Secondary and unspec malig neop of superficial parotid LN |
| B5601 | Secondary and unspec malignant neoplasm mastoid lymph nodes |
| B5602 | Secondary and unspec malig neop superficial cervical LN |
| B5603 | Secondary and unspec malignant neoplasm occipital lymph node |
| B5604 | Secondary and unspec malig neop deep parotid lymph nodes |
| B5605 | Secondary and unspec malig neop submandibular lymph nodes |
| B5606 | Secondary and unspec malig neop of facial lymph nodes |
| B5607 | Secondary and unspec malig neop submental lymph nodes |
| B5608 | Secondary and unspec malig neop anterior cervical LN |
| B5609 | Secondary and unspec malig neop deep cervical LN |
| B560z | Secondary unspec malig neop lymph nodes head/face/neck NOS |
| B561. | Secondary and unspec malig neop intrathoracic lymph nodes |
| B5610 | Secondary and unspec malig neop internal mammary lymph nodes |
| B5611 | Secondary and unspec malig neop intercostal lymph nodes |
| B5612 | Secondary and unspec malig neop diaphragmatic lymph nodes |
| B5613 | Secondary and unspec malig neop ant mediastinal lymph nodes |
| B5614 | Secondary and unspec malig neop post mediastinal lymph nodes |
| B5615 | Secondary and unspec malig neop paratracheal lymph nodes |
| B5616 | Secondary and unspec malig neop superfic tracheobronchial LN |
| B5617 | Secondary and unspec malig neop inferior tracheobronchial LN |
| B5618 | Secondary and unspec malig neop bronchopulmonary lymph nodes |
| B5619 | Secondary and unspec malig neop pulmonary lymph nodes |
| B561z | Secondary and unspec malig neop intrathoracic LN NOS |
| B562. | Secondary and unspec malig neop intra-abdominal lymph nodes |
| B5620 | Secondary and unspec malig neop coeliac lymph nodes |
| B5621 | Secondary and unspec malig neop superficial mesenteric LN |
| B5622 | Secondary and unspec malig neop inferior mesenteric LN |
| B5623 | Secondary and unspec malig neop common iliac lymph nodes |
| B5624 | Secondary and unspec malig neop external iliac lymph nodes |
| B562z | Secondary and unspec malig neop intra-abdominal LN NOS |
| B563. | Secondary and unspec malig neop axilla and upper limb LN |
| B5630 | Secondary and unspec malig neop axillary lymph nodes |
| B5631 | Secondary and unspec malig neop supratrochlear lymph nodes |
| B5632 | Secondary and unspec malig neop infraclavicular lymph nodes |
| B5633 | Secondary and unspec malig neop pectoral lymph nodes |
| B563z | Secondary and unspec malig neop axilla and upper limb LN NOS |
| B564. | Secondary and unspec malig neop inguinal and lower limb LN |
| B5640 | Secondary and unspec malig neop superficial inguinal LN |
| B5641 | Secondary and unspec malig neop deep inguinal lymph nodes |
| B5642 | Secondary and unspec malig neop popliteal lymph nodes |
| B564z | Secondary and unspec malig neop of inguinal and leg LN NOS |
| B565. | Secondary and unspec malig neop intrapelvic lymph nodes |
| B5650 | Secondary and unspec malig neop internal iliac lymph nodes |
| B5651 | Secondary and unspec malig neop inferior epigastric LN |
| B5652 | Secondary and unspec malig neop circumflex iliac LN |
| B5653 | Secondary and unspec malig neop sacral lymph nodes |
| B5654 | Secondary and unspec malig neop obturator lymph nodes |
| B565z | Secondary and unspec malig neop intrapelvic LN NOS |
| B56y. | Secondary and unspec malig neop lymph nodes multiple sites |
| B56z. | Secondary and unspec malig neop lymph nodes NOS |
| B57.. | Secondary malig neop of respiratory and digestive systems |
| B570. | Secondary malignant neoplasm of lung |
| B571. | Secondary malignant neoplasm of mediastinum |
| B572. | Secondary malignant neoplasm of pleura |
| B573. | Secondary malignant neoplasm of other respiratory organs |
| B574. | Secondary malignant neoplasm of small intestine and duodenum |
| B5740 | Secondary malignant neoplasm of duodenum |
| B5741 | Secondary malignant neoplasm of jejunum |
| B5742 | Secondary malignant neoplasm of ileum |
| B574z | Secondary malig neop of small intestine or duodenum NOS |
| B575. | Secondary malignant neoplasm of large intestine and rectum |
| B5750 | Secondary malignant neoplasm of colon |
| B5751 | Secondary malignant neoplasm of rectum |
| B575z | Secondary malig neop of large intestine or rectum NOS |
| B576. | Secondary malig neop of retroperitoneum and peritoneum |
| B5760 | Secondary malignant neoplasm of retroperitoneum |
| B5761 | Secondary malignant neoplasm of peritoneum |
| B5762 | Malignant ascites |
| B576z | Secondary malig neop of retroperitoneum or peritoneum NOS |
| B577. | Secondary malignant neoplasm of liver |
| B57y. | Secondary malignant neoplasm of other digestive organ |
| B57z. | Secondary malig neop of respiratory or digestive system NOS |
| B58.. | Secondary malignant neoplasm of other specified sites |
| B580. | Secondary malignant neoplasm of kidney |
| B581. | Secondary malignant neoplasm of other urinary organs |
| B5810 | Secondary malignant neoplasm of ureter |
| B5811 | Secondary malignant neoplasm of bladder |
| B5812 | Secondary malignant neoplasm of urethra |
| B581z | Secondary malignant neoplasm of other urinary organ NOS |
| B582. | Secondary malignant neoplasm of skin |
| B5820 | Secondary malignant neoplasm of skin of head |
| B5821 | Secondary malignant neoplasm of skin of face |
| B5822 | Secondary malignant neoplasm of skin of neck |
| B5823 | Secondary malignant neoplasm of skin of trunk |
| B5824 | Secondary malignant neoplasm of skin of shoulder and arm |
| B5825 | Secondary malignant neoplasm of skin of hip and leg |
| B5826 | Secondary malignant neoplasm of skin of breast |
| B582z | Secondary malignant neoplasm of skin NOS |
| B583. | Secondary malignant neoplasm of brain and spinal cord |
| B5830 | Secondary malignant neoplasm of brain |
| B5831 | Secondary malignant neoplasm of spinal cord |
| B5832 | Cerebral metastasis |
| B583z | Secondary malignant neoplasm of brain or spinal cord NOS |
| B584. | Secondary malignant neoplasm of other part of nervous system |
| B585. | Secondary malignant neoplasm of bone and bone marrow |
| B5850 | Pathological fracture due to metastatic bone disease |
| B586. | Secondary malignant neoplasm of ovary |
| B587. | Secondary malignant neoplasm of adrenal gland |
| B58y. | Secondary malignant neoplasm of other specified sites |
| B58y0 | Secondary malignant neoplasm of breast |
| B58y1 | Secondary malignant neoplasm of uterus |
| B58y2 | Secondary malignant neoplasm of cervix uteri |
| B58y3 | Secondary malignant neoplasm of vagina |
| B58y4 | Secondary malignant neoplasm of vulva |
| B58y5 | Secondary malignant neoplasm of prostate |
| B58y6 | Secondary malignant neoplasm of testis |
| B58y7 | Secondary malignant neoplasm of penis |
| B58y8 | Secondary malignant neoplasm of epididymis and vas deferens |
| B58y9 | Secondary malignant neoplasm of tongue |
| B58yz | Secondary malignant neoplasm of other specified site NOS |
| B58z. | Secondary malignant neoplasm of other specified site NOS |
| B59.. | Malignant neoplasm of unspecified site |
| B590. | Disseminated malignancy NOS |
| B591. | Other malignant neoplasm NOS |
| B592. | Malignant neoplasms of independent (primary) multiple sites |
| B592X | Kaposi's sarcoma of multiple organs |
| B593. | Primary malignant neoplasm of unknown site |
| B594. | Secondary malignant neoplasm of unknown site |
| B59z. | Malignant neoplasm of unspecified site NOS |
| B59zX | Kaposi's sarcoma, unspecified |
| B5y.. | Malignant neoplasm of other and unspecified site OS |
| B5z.. | Malignant neoplasm of other and unspecified site NOS |
| B6... | Malignant neoplasm of lymphatic and haemopoietic tissue |
| B60.. | Lymphosarcoma and reticulosarcoma |
| B600. | Reticulosarcoma |
| B6000 | Reticulosarcoma of unspecified site |
| B6001 | Reticulosarcoma of lymph nodes of head, face and neck |
| B6002 | Reticulosarcoma of intrathoracic lymph nodes |
| B6003 | Reticulosarcoma of intra-abdominal lymph nodes |
| B6004 | Reticulosarcoma of lymph nodes of axilla and upper limb |
| B6005 | Reticulosarcoma of lymph nodes of inguinal region and leg |
| B6006 | Reticulosarcoma of intrapelvic lymph nodes |
| B6007 | Reticulosarcoma of spleen |
| B6008 | Reticulosarcoma of lymph nodes of multiple sites |
| B600z | Reticulosarcoma NOS |
| B601. | Lymphosarcoma |
| B6010 | Lymphosarcoma of unspecified site |
| B6011 | Lymphosarcoma of lymph nodes of head, face and neck |
| B6012 | Lymphosarcoma of intrathoracic lymph nodes |
| B6013 | Lymphosarcoma of intra-abdominal lymph nodes |
| B6014 | Lymphosarcoma of lymph nodes of axilla and upper limb |
| B6015 | Lymphosarcoma of lymph nodes of inguinal region and leg |
| B6016 | Lymphosarcoma of intrapelvic lymph nodes |
| B6017 | Lymphosarcoma of spleen |
| B6018 | Lymphosarcoma of lymph nodes of multiple sites |
| B601z | Lymphosarcoma NOS |
| B602. | Burkitt's lymphoma |
| B6020 | Burkitt's lymphoma of unspecified site |
| B6021 | Burkitt's lymphoma of lymph nodes of head, face and neck |
| B6022 | Burkitt's lymphoma of intrathoracic lymph nodes |
| B6023 | Burkitt's lymphoma of intra-abdominal lymph nodes |
| B6024 | Burkitt's lymphoma of lymph nodes of axilla and upper limb |
| B6025 | Burkitt's lymphoma of lymph nodes of inguinal region and leg |
| B6026 | Burkitt's lymphoma of intrapelvic lymph nodes |
| B6027 | Burkitt's lymphoma of spleen |
| B6028 | Burkitt's lymphoma of lymph nodes of multiple sites |
| B602z | Burkitt's lymphoma NOS |
| B60y. | Other specified reticulosarcoma or lymphosarcoma |
| B60z. | Reticulosarcoma or lymphosarcoma NOS |
| B61.. | Hodgkin's disease |
| B610. | Hodgkin's paragranuloma |
| B6100 | Hodgkin's paragranuloma of unspecified site |
| B6101 | Hodgkin's paragranuloma of lymph nodes of head, face, neck |
| B6102 | Hodgkin's paragranuloma of intrathoracic lymph nodes |
| B6103 | Hodgkin's paragranuloma of intra-abdominal lymph nodes |
| B6104 | Hodgkin's paragranuloma of lymph nodes of axilla and arm |
| B6105 | Hodgkin's paragranuloma lymph nodes inguinal region and leg |
| B6106 | Hodgkin's paragranuloma of intrapelvic lymph nodes |
| B6107 | Hodgkin's paragranuloma of spleen |
| B6108 | Hodgkin's paragranuloma of lymph nodes of multiple sites |
| B610z | Hodgkin's paragranuloma NOS |
| B611. | Hodgkin's granuloma |
| B6110 | Hodgkin's granuloma of unspecified site |
| B6111 | Hodgkin's granuloma of lymph nodes of head, face and neck |
| B6112 | Hodgkin's granuloma of intrathoracic lymph nodes |
| B6113 | Hodgkin's granuloma of intra-abdominal lymph nodes |
| B6114 | Hodgkin's granuloma of lymph nodes of axilla and upper limb |
| B6115 | Hodgkin's granuloma lymph nodes of inguinal region and leg |
| B6116 | Hodgkin's granuloma of intrapelvic lymph nodes |
| B6117 | Hodgkin's granuloma of spleen |
| B6118 | Hodgkin's granuloma of lymph nodes of multiple sites |
| B611z | Hodgkin's granuloma NOS |
| B612. | Hodgkin's sarcoma |
| B6120 | Hodgkin's sarcoma of unspecified site |
| B6121 | Hodgkin's sarcoma of lymph nodes of head, face and neck |
| B6122 | Hodgkin's sarcoma of intrathoracic lymph nodes |
| B6123 | Hodgkin's sarcoma of intra-abdominal lymph nodes |
| B6124 | Hodgkin's sarcoma of lymph nodes of axilla and upper limb |
| B6125 | Hodgkin's sarcoma of lymph nodes of inguinal region and leg |
| B6126 | Hodgkin's sarcoma of intrapelvic lymph nodes |
| B6127 | Hodgkin's sarcoma of spleen |
| B6128 | Hodgkin's sarcoma of lymph nodes of multiple sites |
| B612z | Hodgkin's sarcoma NOS |
| B613. | Hodgkin's disease, lymphocytic-histiocytic predominance |
| B6130 | Hodgkin's, lymphocytic-histiocytic predominance unspec site |
| B6131 | Hodgkin's, lymphocytic-histiocytic pred of head, face, neck |
| B6132 | Hodgkin's, lymphocytic-histiocytic pred intrathoracic nodes |
| B6133 | Hodgkin's, lymphocytic-histiocytic pred intra-abdominal node |
| B6134 | Hodgkin's, lymphocytic-histiocytic pred axilla and arm |
| B6135 | Hodgkin's, lymphocytic-histiocytic pred inguinal and leg |
| B6136 | Hodgkin's, lymphocytic-histiocytic pred intrapelvic nodes |
| B6137 | Hodgkin's, lymphocytic-histiocytic predominance of spleen |
| B6138 | Hodgkin's, lymphocytic-histiocytic pred of multiple sites |
| B613z | Hodgkin's, lymphocytic-histiocytic predominance NOS |
| B614. | Hodgkin's disease, nodular sclerosis |
| B6140 | Hodgkin's disease, nodular sclerosis of unspecified site |
| B6141 | Hodgkin's nodular sclerosis of head, face and neck |
| B6142 | Hodgkin's nodular sclerosis of intrathoracic lymph nodes |
| B6143 | Hodgkin's nodular sclerosis of intra-abdominal lymph nodes |
| B6144 | Hodgkin's nodular sclerosis of lymph nodes of axilla and arm |
| B6145 | Hodgkin's nodular sclerosis of inguinal region and leg |
| B6146 | Hodgkin's nodular sclerosis of intrapelvic lymph nodes |
| B6147 | Hodgkin's disease, nodular sclerosis of spleen |
| B6148 | Hodgkin's nodular sclerosis of lymph nodes of multiple sites |
| B614z | Hodgkin's disease, nodular sclerosis NOS |
| B615. | Hodgkin's disease, mixed cellularity |
| B6150 | Hodgkin's disease, mixed cellularity of unspecified site |
| B6151 | Hodgkin's mixed cellularity of lymph nodes head, face, neck |
| B6152 | Hodgkin's mixed cellularity of intrathoracic lymph nodes |
| B6153 | Hodgkin's mixed cellularity of intra-abdominal lymph nodes |
| B6154 | Hodgkin's mixed cellularity of lymph nodes of axilla and arm |
| B6155 | Hodgkin's mixed cellularity of lymph nodes inguinal and leg |
| B6156 | Hodgkin's mixed cellularity of intrapelvic lymph nodes |
| B6157 | Hodgkin's disease, mixed cellularity of spleen |
| B6158 | Hodgkin's mixed cellularity of lymph nodes of multiple sites |
| B615z | Hodgkin's disease, mixed cellularity NOS |
| B616. | Hodgkin's disease, lymphocytic depletion |
| B6160 | Hodgkin's lymphocytic depletion of unspecified site |
| B6161 | Hodgkin's lymphocytic depletion of head, face and neck |
| B6162 | Hodgkin's lymphocytic depletion of intrathoracic lymph nodes |
| B6163 | Hodgkin's lymphocytic depletion intra-abdominal lymph nodes |
| B6164 | Hodgkin's lymphocytic depletion lymph nodes axilla and arm |
| B6165 | Hodgkin's lymphocytic depletion lymph nodes inguinal and leg |
| B6166 | Hodgkin's lymphocytic depletion of intrapelvic lymph nodes |
| B6167 | Hodgkin's disease, lymphocytic depletion of spleen |
| B6168 | Hodgkin's lymphocytic depletion lymph nodes multiple sites |
| B616z | Hodgkin's disease, lymphocytic depletion NOS |
| B61z. | Hodgkin's disease NOS |
| B61z0 | Hodgkin's disease NOS, unspecified site |
| B61z1 | Hodgkin's disease NOS of lymph nodes of head, face and neck |
| B61z2 | Hodgkin's disease NOS of intrathoracic lymph nodes |
| B61z3 | Hodgkin's disease NOS of intra-abdominal lymph nodes |
| B61z4 | Hodgkin's disease NOS of lymph nodes of axilla and arm |
| B61z5 | Hodgkin's disease NOS of lymph nodes inguinal region and leg |
| B61z6 | Hodgkin's disease NOS of intrapelvic lymph nodes |
| B61z7 | Hodgkin's disease NOS of spleen |
| B61z8 | Hodgkin's disease NOS of lymph nodes of multiple sites |
| B61zz | Hodgkin's disease NOS |
| B62.. | Other malignant neoplasm of lymphoid and histiocytic tissue |
| B620. | Nodular lymphoma (Brill - Symmers disease) |
| B6200 | Nodular lymphoma of unspecified site |
| B6201 | Nodular lymphoma of lymph nodes of head, face and neck |
| B6202 | Nodular lymphoma of intrathoracic lymph nodes |
| B6203 | Nodular lymphoma of intra-abdominal lymph nodes |
| B6204 | Nodular lymphoma of lymph nodes of axilla and upper limb |
| B6205 | Nodular lymphoma of lymph nodes of inguinal region and leg |
| B6206 | Nodular lymphoma of intrapelvic lymph nodes |
| B6207 | Nodular lymphoma of spleen |
| B6208 | Nodular lymphoma of lymph nodes of multiple sites |
| B620z | Nodular lymphoma NOS |
| B621. | Mycosis fungoides |
| B6210 | Mycosis fungoides of unspecified site |
| B6211 | Mycosis fungoides of the lymph nodes of head, face and neck |
| B6212 | Mycosis fungoides of intrathoracic lymph nodes |
| B6213 | Mycosis fungoides of intra-abdominal lymph nodes |
| B6214 | Mycosis fungoides of lymph nodes of axilla and upper limb |
| B6215 | Mycosis fungoides of lymph nodes of inguinal region and leg |
| B6216 | Mycosis fungoides of intrapelvic lymph nodes |
| B6217 | Mycosis fungoides of spleen |
| B6218 | Mycosis fungoides of lymph nodes of multiple sites |
| B621z | Mycosis fungoides NOS |
| B622. | Sezary's disease |
| B6220 | Sezary's disease of unspecified site |
| B6221 | Sezary's disease of lymph nodes of head, face and neck |
| B6222 | Sezary's disease of intrathoracic lymph nodes |
| B6223 | Sezary's disease of intra-abdominal lymph nodes |
| B6224 | Sezary's disease of lymph nodes of axilla and upper limb |
| B6225 | Sezary's disease of lymph nodes of inguinal region and leg |
| B6226 | Sezary's disease of intrapelvic lymph nodes |
| B6227 | Sezary's disease of spleen |
| B6228 | Sezary's disease of lymph nodes of multiple sites |
| B622z | Sezary's disease NOS |
| B623. | Malignant histiocytosis |
| B6230 | Malignant histiocytosis of unspecified site |
| B6231 | Malignant histiocytosis of lymph nodes head, face and neck |
| B6232 | Malignant histiocytosis of intrathoracic lymph nodes |
| B6233 | Malignant histiocytosis of intra-abdominal lymph nodes |
| B6234 | Malignant histiocytosis of lymph nodes of axilla and arm |
| B6235 | Malignant histiocytosis of lymph nodes inguinal and leg |
| B6236 | Malignant histiocytosis of intrapelvic lymph nodes |
| B6237 | Malignant histiocytosis of spleen |
| B6238 | Malignant histiocytosis of lymph nodes of multiple sites |
| B623z | Malignant histiocytosis NOS |
| B624. | Leukaemic reticuloendotheliosis |
| B6240 | Leukaemic reticuloendotheliosis of unspecified sites |
| B6241 | Leukaemic reticuloend of lymph nodes of head, face and neck |
| B6242 | Leukaemic reticuloendotheliosis of intrathoracic lymph nodes |
| B6243 | Leukaemic reticuloend of intra-abdominal lymph nodes |
| B6244 | Leukaemic reticuloend of lymph nodes of axilla and arm |
| B6245 | Leukaemic reticuloend of lymph nodes inguinal region and leg |
| B6246 | Leukaemic reticuloendotheliosis of intrapelvic lymph nodes |
| B6247 | Leukaemic reticuloendotheliosis of spleen |
| B6248 | Leukaemic reticuloend of lymph nodes of multiple sites |
| B624z | Leukaemic reticuloendotheliosis NOS |
| B625. | Letterer-Siwe disease |
| B6250 | Letterer-Siwe disease of unspecified sites |
| B6251 | Letterer-Siwe disease of lymph nodes of head, face and neck |
| B6252 | Letterer-Siwe disease of intrathoracic lymph nodes |
| B6253 | Letterer-Siwe disease of intra-abdominal lymph nodes |
| B6254 | Letterer-Siwe disease of lymph nodes of axilla and arm |
| B6255 | Letterer-Siwe disease of lymph nodes inguinal region and leg |
| B6256 | Letterer-Siwe disease of intrapelvic lymph nodes |
| B6257 | Letterer-Siwe disease of spleen |
| B6258 | Letterer-Siwe disease of lymph nodes of multiple sites |
| B625z | Letterer-Siwe disease NOS |
| B626. | Malignant mast cell tumours |
| B6260 | Mast cell malignancy of unspecified site |
| B6261 | Mast cell malignancy of lymph nodes of head, face and neck |
| B6262 | Mast cell malignancy of intrathoracic lymph nodes |
| B6263 | Mast cell malignancy of intra-abdominal lymph nodes |
| B6264 | Mast cell malignancy of lymph nodes of axilla and upper limb |
| B6265 | Mast cell malignancy of lymph nodes inguinal region and leg |
| B6266 | Mast cell malignancy of intrapelvic lymph nodes |
| B6267 | Mast cell malignancy of spleen |
| B6268 | Mast cell malignancy of lymph nodes of multiple sites |
| B626z | Malignant mast cell tumour NOS |
| B627. | Non - Hodgkin's lymphoma |
| B6270 | Follicular non-Hodgkin's small cleaved cell lymphoma |
| B6271 | Follicular non-Hodg mixed sml cleavd & lge cell lymphoma |
| B6272 | Follicular non-Hodgkin's large cell lymphoma |
| B6273 | Diffuse non-Hodgkin's small cell (diffuse) lymphoma |
| B6274 | Diffuse non-Hodgkin's small cleaved cell (diffuse) lymphoma |
| B6275 | Diffuse non-Hodgkin mixed sml & lge cell (diffuse) lymphoma |
| B6276 | Diffuse non-Hodgkin's immunoblastic (diffuse) lymphoma |
| B6277 | Diffuse non-Hodgkin's lymphoblastic (diffuse) lymphoma |
| B6278 | Diffuse non-Hodgkin's lymphoma undifferentiated (diffuse) |
| B6279 | Mucosa-associated lymphoma |
| B627A | Diffuse non-Hodgkin's large cell lymphoma |
| B627B | Other types of follicular non-Hodgkin's lymphoma |
| B627C | Follicular non-Hodgkin's lymphoma |
| B627D | Diffuse non-Hodgkin's centroblastic lymphoma |
| B627E | Diffuse large B-cell lymphoma |
| B627W | Unspecified B-cell non-Hodgkin's lymphoma |
| B627X | Diffuse non-Hodgkin's lymphoma, unspecified |
| B62x. | Malignant lymphoma otherwise specified |
| B62x0 | T-zone lymphoma |
| B62x1 | Lymphoepithelioid lymphoma |
| B62x2 | Peripheral T-cell lymphoma |
| B62x3 | Malignant reticuloendotheliosis |
| B62x4 | Malignant reticulosis |
| B62x5 | Malignant immunoproliferative small intestinal disease |
| B62x6 | True histiocytic lymphoma |
| B62xX | Oth and unspecif peripheral & cutaneous T-cell lymphomas |
| B62y. | Malignant lymphoma NOS |
| B62y0 | Malignant lymphoma NOS of unspecified site |
| B62y1 | Malignant lymphoma NOS of lymph nodes of head, face and neck |
| B62y2 | Malignant lymphoma NOS of intrathoracic lymph nodes |
| B62y3 | Malignant lymphoma NOS of intra-abdominal lymph nodes |
| B62y4 | Malignant lymphoma NOS of lymph nodes of axilla and arm |
| B62y5 | Malignant lymphoma NOS of lymph node inguinal region and leg |
| B62y6 | Malignant lymphoma NOS of intrapelvic lymph nodes |
| B62y7 | Malignant lymphoma NOS of spleen |
| B62y8 | Malignant lymphoma NOS of lymph nodes of multiple sites |
| B62yz | Malignant lymphoma NOS |
| B62z. | Malignant neoplasms of lymphoid and histiocytic tissue NOS |
| B62z0 | Unspec malig neop lymphoid/histiocytic of unspecified site |
| B62z1 | Unspec malig neop lymphoid/histiocytic lymph node head/neck |
| B62z2 | Unspec malig neop lymphoid/histiocytic of intrathoracic node |
| B62z3 | Unspec malig neop lymphoid/histiocytic intra-abdominal nodes |
| B62z4 | Unspec malig neop lymphoid/histiocytic lymph node axilla/arm |
| B62z5 | Unspec malig neop lymphoid/histiocytic nodes inguinal/leg |
| B62z6 | Unspec malig neop lymphoid/histiocytic of intrapelvic nodes |
| B62z7 | Unspec malig neop lymphoid/histiocytic of spleen |
| B62z8 | Unspec malig neop lymphoid/histiocytic of multiple sites |
| B62zz | Lymphoid and histiocytic malignancy NOS |
| B63.. | Multiple myeloma and immunoproliferative neoplasms |
| B630. | Multiple myeloma |
| B6300 | Malignant plasma cell neoplasm, extramedullary plasmacytoma |
| B6301 | Solitary myeloma |
| B6302 | Plasmacytoma NOS |
| B6303 | Lambda light chain myeloma |
| B631. | Plasma cell leukaemia |
| B63y. | Other immunoproliferative neoplasms |
| B63z. | Immunoproliferative neoplasm or myeloma NOS |
| B64.. | Lymphoid leukaemia |
| B640. | Acute lymphoid leukaemia |
| B641. | Chronic lymphoid leukaemia |
| B642. | Subacute lymphoid leukaemia |
| B64y. | Other lymphoid leukaemia |
| B64y0 | Aleukaemic lymphoid leukaemia |
| B64y1 | Prolymphocytic leukaemia |
| B64y2 | Adult T-cell leukaemia |
| B64yz | Other lymphoid leukaemia NOS |
| B64z. | Lymphoid leukaemia NOS |
| B65.. | Myeloid leukaemia |
| B650. | Acute myeloid leukaemia |
| B651. | Chronic myeloid leukaemia |
| B6510 | Chronic eosinophilic leukaemia |
| B6512 | Chronic neutrophilic leukaemia |
| B651z | Chronic myeloid leukaemia NOS |
| B652. | Subacute myeloid leukaemia |
| B653. | Myeloid sarcoma |
| B6530 | Chloroma |
| B6531 | Granulocytic sarcoma |
| B653z | Myeloid sarcoma NOS |
| B65y. | Other myeloid leukaemia |
| B65y0 | Aleukaemic myeloid leukaemia |
| B65y1 | Acute promyelocytic leukaemia |
| B65yz | Other myeloid leukaemia NOS |
| B65z. | Myeloid leukaemia NOS |
| B66.. | Monocytic leukaemia |
| B660. | Acute monocytic leukaemia |
| B661. | Chronic monocytic leukaemia |
| B662. | Subacute monocytic leukaemia |
| B66y. | Other monocytic leukaemia |
| B66y0 | Aleukaemic monocytic leukaemia |
| B66yz | Other monocytic leukaemia NOS |
| B66z. | Monocytic leukaemia NOS |
| B67.. | Other specified leukaemia |
| B670. | Acute erythraemia and erythroleukaemia |
| B671. | Chronic erythraemia |
| B672. | Megakaryocytic leukaemia |
| B673. | Mast cell leukaemia |
| B674. | Acute panmyelosis |
| B675. | Acute myelofibrosis |
| B67y. | Other and unspecified leukaemia |
| B67y0 | Lymphosarcoma cell leukaemia |
| B67yz | Other and unspecified leukaemia NOS |
| B67z. | Other specified leukaemia NOS |
| B68.. | Leukaemia of unspecified cell type |
| B680. | Acute leukaemia NOS |
| B681. | Chronic leukaemia NOS |
| B682. | Subacute leukaemia NOS |
| B68y. | Other leukaemia of unspecified cell type |
| B68z. | Leukaemia NOS |
| B69.. | Myelomonocytic leukaemia |
| B690. | Acute myelomonocytic leukaemia |
| B691. | Chronic myelomonocytic leukaemia |
| B692. | Subacute myelomonocytic leukaemia |
| B6y.. | Malignant neoplasm lymphatic or haematopoietic tissue OS |
| B6y0. | Myeloproliferative disorder |
| B6y1. | Myelosclerosis with myeloid metaplasia |
| B6z.. | Malignant neoplasm lymphatic or haematopoietic tissue NOS |
| B6z0. | Kaposi's sarcoma of lymph nodes |
| B831. | Carcinoma in situ of cervix uteri |
| B8310 | Carcinoma in situ of endocervix |
| B8311 | Carcinoma in situ of exocervix |
| ByuFA | [X]Carcinoma in situ of other parts of cervix |
| BB2K. | [M]Queyrat's erythroplasia |
| BBG4. | [M]Periosteal fibroma |
| BBG5. | [M]Periosteal fibrosarcoma |
| BBGN. | [M]Myofibromatosis |
| BBGP. | [M]Pigmented dermatofibrosarcoma protuberans |
| BBm1. | [M]Malignant histiocytosis |
| BBm4. | [M]True histiocytic lymphoma |
| BBM6. | [M]Mucinous adenofibroma |
| BBM7. | [M]Cellular intracanalicular fibroadenoma |
| BBM8. | [M]Cystosarcoma phyllodes NOS |
| BBmA. | [M] Refractory anaemia with sideroblasts |
| BBmB. | [M]Refractory anaemia+excess of blasts with transformation |
| BBmC. | [M] T-gamma lymphoproliferative disease |
| BBmE. | [M] Gamma heavy chain disease |
| BBmF. | [M] Angiocentric immunoproliferative lesion |
| BBmG. | [M] Immunoproliferative small intestinal disease |
| BBmK. | [M]Waldenstrom's macroglobulinaemia |
| BBmz. | [M]Miscellaneous reticuloendothelial neoplasm NOS |
| BBN0. | [M]Synovioma, benign |
| BBN1. | [M]Synovial sarcoma NOS |
| BBN2. | [M]Synovial sarcoma, spindle cell type |
| BBN3. | [M]Synovial sarcoma, epithelioid cell type |
| BBNz. | [M]Synovial neoplasm NOS |
| BBrA6 | [M]Acute panmyelosis |
| BBrA7 | [M]Acute myelofibrosis |
| BBrA8 | [M]Leukaemic reticuloendotheliosis |
| BBs.. | [M]Misc myeloproliferative and lymphoproliferative disorders |
| BBs0. | [M]Polycythaemia vera |
| BBS1. | [M]Mesonephric tumour |
| BBS2. | [M]Mesonephroma, malignant |
| BBs4. | [M]Idiopathic thrombocythaemia |
| BBs5. | [M]Chronic lymphoproliferative disease |
| BBsz. | [M]Misc myeloproliferative or lymphoproliferative dis NOS |
| ByuD. | [X]Malignant neoplasms of lymphoid, haematopoietic and rela |
| ByuD4 | [X]Other malignant immunoproliferative diseases |
| ByuDA | [X]Oth spcf mal neoplsm/lymphoid,haematopoietic+rltd tissue |
| ByuDB | [X]Mal neoplasm/lymphoid,haematopoietic+related tissu,unspcf |
| BBR.. | [M]Trophoblastic neoplasms |
| BBr0. | [M]Leukaemias unspecified |
| BBr00 | [M]Leukaemia NOS |
| BBr01 | [M]Acute leukaemia NOS |
| BBr02 | [M]Subacute leukaemia NOS |
| BBr03 | [M]Chronic leukaemia NOS |
| BBr04 | [M]Aleukaemic leukaemia NOS |
| BBr0z | [M]Leukaemia unspecified, NOS |
| BBR1. | [M]Invasive hydatidiform mole |
| BBr10 | [M]Compound leukaemia |
| BBr1z | [M]Compound leukaemia NOS |
| BBr2. | [M]Lymphoid leukaemias |
| BBr20 | [M]Lymphoid leukaemia NOS |
| BBr21 | [M]Acute lymphoid leukaemia |
| BBr22 | [M]Subacute lymphoid leukaemia |
| BBr23 | [M]Chronic lymphoid leukaemia |
| BBr24 | [M]Aleukaemic lymphoid leukaemia |
| BBr25 | [M]Prolymphocytic leukaemia |
| BBr26 | [M]Burkitt's cell leukaemia |
| BBr27 | [M]Adult T-cell leukaemia/lymphoma |
| BBr2z | [M]Other lymphoid leukaemia NOS |
| BBr3. | [M]Plasma cell leukaemias |
| BBr30 | [M]Plasma cell leukaemia |
| BBr3z | [M]Plasma cell leukaemia NOS |
| BBR4. | [M]Malignant teratoma, trophoblastic |
| BBr40 | [M]Erythroleukaemia |
| BBr41 | [M]Acute erythraemia |
| BBr42 | [M]Chronic erythraemia |
| BBr4z | [M]Erythroleukaemia NOS |
| BBR5. | [M]Partial hydatidiform mole |
| BBr50 | [M]Lymphosarcoma cell leukaemia |
| BBr5z | [M]Lymphosarcoma cell leukaemia NOS |
| BBR6. | [M]Placental site trophoblastic tumour |
| BBr60 | [M]Myeloid leukaemia NOS |
| BBr61 | [M]Acute myeloid leukaemia |
| BBr62 | [M]Subacute myeloid leukaemia |
| BBr63 | [M]Chronic myeloid leukaemia |
| BBr64 | [M]Aleukaemic myeloid leukaemia |
| BBr65 | [M]Neutrophilic leukaemia |
| BBr66 | [M]Acute promyelocytic leukaemia |
| BBr67 | [M]Acute myelomonocytic leukaemia |
| BBr68 | [M]Chronic myelomonocytic leukaemia |
| BBr6z | [M]Other myeloid leukaemia NOS |
| BBR7. | [M]Classical hydatidiform mole |
| BBr70 | [M]Basophilic leukaemia |
| BBr7z | [M]Basophilic leukaemia NOS |
| BBr8. | [M]Eosinophilic leukaemias |
| BBr80 | [M]Eosinophilic leukaemia |
| BBr8z | [M]Eosinophilic leukaemia NOS |
| BBr9. | [M]Monocytic leukaemias |
| BBr90 | [M]Monocytic leukaemia NOS |
| BBr91 | [M]Acute monocytic leukaemia |
| BBr92 | [M]Subacute monocytic leukaemia |
| BBr93 | [M]Chronic monocytic leukaemia |
| BBr94 | [M]Aleukaemic monocytic leukaemia |
| BBr9z | [M]Other monocytic leukaemia NOS |
| BBrA. | [M]Miscellaneous leukaemias |
| BBrA0 | [M]Mast cell leukaemia |
| BBrA1 | [M]Megakaryocytic leukaemia |
| BBrA2 | [M]Megakaryocytic myelosis |
| BBrA4 | [M]Hairy cell leukaemia |
| BBrA5 | [M]Acute megakaryoblastic leukaemia |
| BBrAz | [M]Miscellaneous leukaemia NOS |
| BBRz. | [M]Trophoblastic neoplasm NOS |
| ByuD5 | [X]Other lymphoid leukaemia |
| ByuD6 | [X]Other myeloid leukaemia |
| ByuD7 | [X]Other monocytic leukaemia |
| ByuD8 | [X]Other specified leukaemias |
| ByuD9 | [X]Other leukaemia of unspecified cell type |
| BBg1. | [M]Malignant lymphoma NOS |
| BBg10 | [M]Malignant lymphoma, diffuse NOS |
| BBg2. | [M]Malignant lymphoma, non Hodgkin's type |
| BBg3. | [M]Malignant lymphoma, undifferentiated cell type NOS |
| BBg7. | [M]Malignant lymphoma, lymphoplasmacytoid type |
| BBg8. | [M]Malignant lymphoma, immunoblastic type |
| BBg9. | [M]Malignant lymphoma, mixed lymphocytic-histiocytic NOS |
| BBgA. | [M]Malignant lymphoma, centroblastic-centrocytic, diffuse |
| BBgB. | [M]Malignant lymphoma, follicular centre cell NOS |
| BBgC. | [M]Malignant lymphoma, lymphocytic, well differentiated NOS |
| BBgD. | [M]Malig lymphoma, lymphocytic, intermediate different NOS |
| BBgE. | [M]Malignant lymphoma, centrocytic |
| BBgF. | [M]Malignant lymphoma, follicular centre cell, cleaved NOS |
| BBgG. | [M]Malignant lymphoma, lymphocytic, poorly different NOS |
| BBgH. | [M]Prolymphocytic lymphosarcoma |
| BBgJ. | [M]Malignant lymphoma, centroblastic type NOS |
| BBgK. | [M]Malig lymphoma, follicular centre cell, non-cleaved NOS |
| BBgL. | [M]Malignant lymphoma, small lymphocytic NOS |
| BBgM. | [M]Malignant lymphoma, small cleaved cell, diffuse |
| BBgR. | [M]Malignant lymphoma, large cell, diffuse NOS |
| BBgS. | [M]Malignant lymphoma, large cell, cleaved, diffuse |
| BBgT. | [M]Malignant lymphoma, large cell, noncleaved, diffuse |
| BBgV. | [M]Malignant lymphoma, small cell, noncleaved, diffuse |
| BBgz. | [M]Lymphoma, diffuse or NOS |
| BBK.. | [M]Myomatous neoplasms |
| BBk0. | [M]Malignant lymphoma, nodular NOS |
| BBk1. | [M]Malig lymphoma, mixed lymphocytic-histiocytic, nodular |
| BBK2. | [M]Myoma and myosarcoma |
| BBK3. | [M]Rhabdomyomatous neoplasms |
| BBk4. | [M]Malig lymp, lymphocytic, intermediate different, nodular |
| BBk5. | [M]Malig lymp, follicular centre cell, cleaved, follicular |
| BBk6. | [M]Malig lymp, lymphocytic, poorly differentiated, nodular |
| BBk7. | [M]Malignant lymphoma, centroblastic type, follicular |
| BBk8. | [M]Malig lymp,follicular centre cell,noncleaved,follicular |
| BBKz. | [M]Myomatous neoplasm NOS |
| BBM5. | [M]Serous adenofibroma |
| BBM9. | [M]Cystosarcoma phyllodes, malignant |
| BBmD. | [M] Cutaneous lymphoma |
| BBmH. | [M] Large cell lymphoma |
| BBQ.. | [M]Germ cell neoplasms |
| BBQ0. | [M]Dysgerminoma |
| BBQz. | [M]Germ cell neoplasm NOS |
| BBV0. | [M]Osteoma NOS |
| BBV2. | [M]Chondroblastic osteosarcoma |
| ByuD1 | [X]Other types of follicular non-Hodgkin's lymphoma |
| ByuD2 | [X]Other types of diffuse non-Hodgkin's lymphoma |
| ByuD3 | [X]Other specified types of non-Hodgkin's lymphoma |
| ByuDC | [X]Diffuse non-Hodgkin's lymphoma, unspecified |
| ByuDD | [X]Oth and unspecif peripheral & cutaneous T-cell lymphomas |
| ByuDE | [X]Unspecified B-cell non-Hodgkin's lymphoma |
| ByuDF | [X]Non-Hodgkin's lymphoma, unspecified type |
| BBJ.. | [M]Lipomatous neoplasms |
| BBJ0. | [M]Lipoma NOS |
| BBJ1. | [M]Liposarcoma NOS |
| BBj10 | [M]Hodgkin,s disease, lymphocytic predominance, diffuse |
| BBj11 | [M]Hodgkin,s disease, lymphocytic predominance, nodular |
| BBj2. | [M]Hodgkin's disease, mixed cellularity |
| BBJ3. | [M]Liposarcoma, well differentiated type |
| BBj4. | [M]Hodgkin's disease,lymphocytic depletion,diffuse fibrosis |
| BBJ5. | [M]Myxoid liposarcoma |
| BBJ6. | [M]Round cell liposarcoma |
| BBj60 | [M]Hodgkin,s disease, nodular sclerosis, lymphocytic predom |
| BBj61 | [M]Hodgkin,s disease, nodular sclerosis, mixed cellularity |
| BBj62 | [M]Hodgkin,s disease, nodular sclerosis, lymphocytic deplet |
| BBJ7. | [M]Pleomorphic liposarcoma |
| BBJ8. | [M]Mixed type liposarcoma |
| BBJ9. | [M]Intramuscular lipoma |
| BBJA. | [M]Spindle cell lipoma |
| BBJz. | [M]Lipomatous neoplasms NOS |
| ByuD0 | [X]Other Hodgkin's disease |
| BB... | [M]Morphology of neoplasms |
| BB0.. | [M]Neoplasms NOS |
| BB02. | [M]Neoplasm, malignant |
| BB03. | [M]Neoplasm, metastatic |
| BB07. | [M]Tumour cells, malignant |
| BB08. | [M]Malignant tumour, small cell type |
| BB09. | [M]Malignant tumour, giant cell type |
| BB0A. | [M]Malignant tumour, fusiform cell type |
| BB0z. | [M]Unspecified tumour cell NOS |
| BB1.. | [M]Epithelial neoplasms NOS |
| BB12. | [M]Carcinoma NOS |
| BB13. | [M]Carcinoma, metastatic, NOS |
| BB14. | [M]Carcinomatosis |
| BB16. | [M]Epithelioma, malignant |
| BB17. | [M]Large cell carcinoma NOS |
| BB18. | [M]Carcinoma, undifferentiated type, NOS |
| BB19. | [M]Carcinoma, anaplastic type, NOS |
| BB1A. | [M]Pleomorphic carcinoma |
| BB1B. | [M]Giant cell and spindle cell carcinoma |
| BB1C. | [M]Giant cell carcinoma |
| BB1D. | [M]Spindle cell carcinoma |
| BB1E. | [M]Pseudosarcomatous carcinoma |
| BB1F. | [M]Polygonal cell carcinoma |
| BB1G. | [M]Spheroidal cell carcinoma |
| BB1H. | [M]Tumourlet |
| BB1J. | [M]Small cell carcinoma NOS |
| BB1K. | [M]Oat cell carcinoma |
| BB1L. | [M]Small cell carcinoma, fusiform cell type |
| BB1M. | [M]Small cell carcinoma, intermediate cell |
| BB1N. | [M]Small cell-large cell carcinoma |
| BB2.. | [M]Papillary and squamous cell neoplasms |
| BB20. | [M]Papilloma NOS (excluding papilloma of urinary bladder) |
| BB22. | [M]Papillary carcinoma NOS |
| BB24. | [M]Verrucous carcinoma NOS |
| BB26. | [M]Papillary squamous cell carcinoma |
| BB2A. | [M]Squamous cell carcinoma NOS |
| BB2B. | [M]Squamous cell carcinoma, metastatic NOS |
| BB2C. | [M]Squamous cell carcinoma, keratinising type NOS |
| BB2D. | [M]Squamous cell carcinoma, large cell, non-keratinising |
| BB2E. | [M]Squamous cell carcinoma, small cell, non-keratinising |
| BB2F. | [M]Squamous cell carcinoma, spindle cell type |
| BB2G. | [M]Adenoid squamous cell carcinoma |
| BB2H. | [M]Squamous cell ca-in-situ, questionable stromal invasion |
| BB2J. | [M]Squamous cell carcinoma, microinvasive |
| BB2M. | [M]Lymphoepithelial carcinoma |
| BB2N. | [M]Intraepit neop,grade III,of cervix, vulva and vagina |
| BB2z. | [M]Papillary or squamous cell neoplasm NOS |
| BB3.. | [M]Basal cell neoplasms |
| BB30. | [M]Basal cell tumour |
| BB31. | [M]Basal cell carcinoma NOS |
| BB32. | [M]Multicentric basal cell carcinoma |
| BB33. | [M]Basal cell carcinoma, morphoea type |
| BB34. | [M]Basal cell carcinoma, fibroepithelial type |
| BB35. | [M]Basosquamous carcinoma |
| BB36. | [M]Metatypical carcinoma |
| BB3z. | [M]Basal cell neoplasm NOS |
| BB4.. | [M]Transitional cell papillomas and carcinomas |
| BB41. | [M]Urothelial papilloma |
| BB43. | [M]Transitional cell carcinoma NOS |
| BB46. | [M]Schneiderian carcinoma |
| BB47. | [M]Transitional cell carcinoma, spindle cell type |
| BB48. | [M]Basaloid carcinoma |
| BB49. | [M]Cloacogenic carcinoma |
| BB4A. | [M]Papillary transitional cell carcinoma |
| BB50. | [M]Adenoma NOS |
| BB500 | [M]Microcystic adenoma |
| BB52. | [M]Adenocarcinoma NOS |
| BB520 | [M]Adenocarcinoma in tubulovillous adenoma |
| BB53. | [M]Adenocarcinoma, metastatic, NOS |
| BB54. | [M]Scirrhous adenocarcinoma |
| BB55. | [M]Linitis plastica |
| BB56. | [M]Superficial spreading adenocarcinoma |
| BB57. | [M]Adenocarcinoma, intestinal type |
| BB58. | [M]Carcinoma, diffuse type |
| BB5a1 | [M]Juxtaglomerular tumour |
| BB5B. | [M]Pancreatic adenomas and carcinomas |
| BB5B4 | [M]Glucagonoma NOS |
| BB5C. | [M]Gastrinoma and carcinomas |
| BB5c0 | [M]Chief cell adenoma |
| BB5C0 | [M]Gastrinoma NOS |
| BB5c2 | [M]Water-clear cell adenocarcinoma |
| BB5d1 | [M]Mixed cell adenocarcinoma |
| BB5f. | [M]Thyroid adenoma and adenocarcinoma |
| BB5f1 | [M]Follicular adenocarcinoma NOS |
| BB5f2 | [M]Follicular adenocarcinoma, well differentiated type |
| BB5f3 | [M]Follicular adenocarcinoma, trabecular type |
| BB5f6 | [M]Papillary and follicular adenocarcinoma |
| BB5f7 | [M]Nonencapsulated sclerosing carcinoma |
| BB5h. | [M]Adrenal cortical tumours |
| BB5hz | [M]Adrenal cortical tumours NOS |
| BB5J. | [M]Adenoid cystic carcinoma |
| BB5j3 | [M]Endometrioid adenofibroma NOS |
| BB5j4 | [M]Endometrioid adenofibroma, borderline malignancy |
| BB5K. | [M]Cribriform carcinoma |
| BB5L1 | [M]Adenocarcinoma in adenomatous polyp |
| BB5L2 | [M]Adenocarcinoma in situ in adenomatous polyp |
| BB5L3 | [M]Adenocarcinoma in multiple adenomatous polyps |
| BB5M1 | [M]Tubular adenocarcinoma |
| BB5P. | [M]Solid carcinoma NOS |
| BB5Q. | [M]Carcinoma simplex |
| BB5R. | [M]Carcinoid tumours |
| BB5R0 | [M]Carcinoid tumour NOS |
| BB5R1 | [M]Carcinoid tumour, malignant |
| BB5R2 | [M]Carcinoid tumour, argentaffin, NOS |
| BB5R3 | [M]Carcinoid tumour, argentaffin, malignant |
| BB5R4 | [M]Carcinoid tumour, nonargentaffin, NOS |
| BB5R5 | [M]Carcinoid tumour, nonargentaffin, malignant |
| BB5R6 | [M]Mucocarcinoid tumour, malignant |
| BB5R8 | [M]Adenocarcinoid tumour |
| BB5R9 | [M]Neuroendocrine carcinoma |
| BB5RA | [M]Merkel cell carcinoma |
| BB5Rz | [M]Carcinoid tumours NOS |
| BB5S. | [M]Respiratory tract adenomas and adenocarcinomas |
| BB5Sz | [M]Respiratory tract adenoma or adenocarcinoma NOS |
| BB5T1 | [M]Papillary adenocarcinoma NOS |
| BB5U1 | [M]Adenocarcinoma in villous adenoma |
| BB5U2 | [M]Villous adenocarcinoma |
| BB5V1 | [M]Chromophobe carcinoma |
| BB5V3 | [M]Acidophil carcinoma |
| BB5V5 | [M]Mixed acidophil-basophil carcinoma |
| BB5V7 | [M]Basophil carcinoma |
| BB5W1 | [M]Oxyphilic adenocarcinoma |
| BB5X1 | [M]Clear cell adenocarcinoma NOS |
| BB5y. | [M]Adenoma and adenocarcinoms OS |
| BB5y0 | [M]Basal cell adenocarcinoma |
| BB5y1 | [M]Vipoma |
| BB5y3 | [M]Apudoma |
| BB5y5 | [M]Lipid-rich carcinoma |
| BB5y6 | [M]Glycogen-rich carcinoma |
| BB6.. | [M]Adnexal and skin appendage neoplasms |
| BB60z | [M]Skin appendage adenoma or carcinoma NOS |
| BB611 | [M]Sweat gland tumour NOS |
| BB621 | [M]Apocrine adenocarcinoma |
| BB63. | [M]Eccrine acrospiroma |
| BB64. | [M]Eccrine spiradenoma |
| BB65. | [M]Hidrocystoma |
| BB66. | [M]Papillary hydradenoma |
| BB67. | [M]Papillary syringadenoma |
| BB68. | [M]Syringoma NOS |
| BB6A1 | [M]Ceruminous adenocarcinoma |
| BB7.. | [M]Mucoepidermoid neoplasms |
| BB70. | [M]Mucoepidermoid tumour |
| BB71. | [M]Mucoepidermoid carcinoma |
| BB7z. | [M]Mucoepidermoid neoplasm NOS |
| BB8.. | [M]Cystic, mucinous and serous neoplasms |
| BB80. | [M]Cystadenoma and carcinoma |
| BB801 | [M]Cystadenocarcinoma NOS |
| BB81. | [M]Ovarian cystic, mucinous and serous neoplasms |
| BB812 | [M]Serous cystadenocarcinoma, NOS |
| BB815 | [M]Papillary cystadenocarcinoma, NOS |
| BB818 | [M]Papillary serous cystadenocarcinoma |
| BB819 | [M]Serous surface papilloma NOS |
| BB81A | [M]Serous surface papilloma, borderline malignancy |
| BB81B | [M]Serous surface papillary carcinoma |
| BB81E | [M]Mucinous cystadenocarcinoma NOS |
| BB81H | [M]Papillary mucinous cystadenocarcinoma |
| BB81J | [M]Serous cystadenoma, borderline malignancy |
| BB81K | [M]Papillary cystadenoma, borderline malignancy |
| BB81L | [M]Papillary cystic tumour |
| BB81M | [M]Papillary serous cystadenoma, borderline malignancy |
| BB81z | [M]Ovarian cystic, mucinous or serous neoplasm NOS |
| BB821 | [M]Mucinous adenocarcinoma |
| BB83. | [M]Pseudomyxoma peritonei |
| BB84. | [M]Mucin-producing adenocarcinoma |
| BB85. | [M]Signet ring carcinoma |
| BB850 | [M]Signet ring cell carcinoma |
| BB851 | [M]Metastatic signet ring cell carcinoma |
| BB85z | [M]Signet ring carcinoma NOS |
| BB8z. | [M]Cystic, mucinous or serous neoplasm NOS |
| BB9.. | [M]Ductal, lobular and medullary neoplasms |
| BB90. | [M]Intraductal carcinoma, noninfiltrating NOS |
| BB91. | [M]Infiltrating duct carcinoma |
| BB910 | [M]Intraductal papillary adenocarcinoma with invasion |
| BB911 | [M]Infiltrating duct and lobular carcinoma |
| BB92. | [M]Comedocarcinoma, noninfiltrating |
| BB93. | [M]Comedocarcinoma NOS |
| BB94. | [M]Juvenile breast carcinoma |
| BB96. | [M]Noninfiltrating intraductal papillary adenocarcinoma |
| BB98. | [M]Noninfiltrating intracystic carcinoma |
| BB9B. | [M]Medullary carcinoma NOS |
| BB9C. | [M]Medullary carcinoma with amyloid stroma |
| BB9D. | [M]Medullary carcinoma with lymphoid stroma |
| BB9E0 | [M]Intraductal carcinoma and lobular carcinoma in situ |
| BB9F. | [M]Lobular carcinoma NOS |
| BB9G. | [M]Infiltrating ductular carcinoma |
| BB9H. | [M]Inflammatory carcinoma |
| BB9J. | [M]Paget's disease, mammary |
| BB9L. | [M]Paget's disease, extramammary, exc Paget's disease bone |
| BB9M. | [M]Intracystic carcinoma NOS |
| BB9z. | [M]Ductal, lobular or medullary neoplasm NOS |
| BBa.. | [M]Miscellaneous tumours |
| BBA.. | [M]Acinar cell neoplasms |
| BBA1. | [M]Acinar cell tumour |
| BBA2. | [M]Acinar cell carcinoma |
| BBa3. | [M]Pineoblastoma |
| BBa4. | [M]Melanotic neuroectodermal tumour |
| BBAz. | [M]Acinar cell neoplasm NOS |
| BBB.. | [M]Complex epithelial neoplasms |
| BBB0. | [M]Adenosquamous carcinoma |
| BBB1. | [M]Adenolymphoma |
| BBB2. | [M]Adenocarcinoma with squamous metaplasia |
| BBB3. | [M]Adenocarcinoma with cartilaginous and osseous metaplasia |
| BBb4. | [M]Subependymal giant cell astrocytoma |
| BBB4. | [M]Adenocarcinoma with spindle cell metaplasia |
| BBb5. | [M]Choroid plexus papilloma NOS |
| BBB5. | [M]Adenocarcinoma with apocrine metaplasia |
| BBb7. | [M]Ependymoma NOS |
| BBB7. | [M]Epithelial-myoepithelial carcinoma |
| BBb8. | [M]Ependymoma, anaplastic type |
| BBb9. | [M]Papillary ependymoma |
| BBbA. | [M]Myxopapillary ependymoma |
| BBbD. | [M]Protoplasmic astrocytoma |
| BBbE. | [M]Gemistocytic astrocytoma |
| BBbz. | [M]Glioma NOS |
| BBBz. | [M]Complex epithelial neoplasm NOS |
| BBc.. | [M]Neuroepitheliomatous neoplasms |
| BBC.. | [M]Specialised gonadal neoplasms |
| BBc0. | [M]Ganglioneuromatous neoplasms |
| BBC0. | [M]Sex cord-stromal tumour |
| BBc00 | [M]Ganglioneuroma |
| BBc1. | [M]Neuroblastoma NOS |
| BBC11 | [M]Theca cell carcinoma |
| BBC12 | [M]Thecoma, luteinized |
| BBC1z | [M]Thecal cell neoplasm NOS |
| BBc2. | [M]Medulloepithelioma NOS |
| BBC2. | [M]Luteoma NOS |
| BBc3. | [M]Teratoid medulloepithelioma |
| BBC3. | [M]Granulosa cell tumour NOS |
| BBC30 | [M]Juvenile granulosa cell tumour |
| BBc4. | [M]Neuroepithelioma NOS |
| BBC4. | [M]Granulosa cell tumour, malignant |
| BBc5. | [M]Spongioneuroblastoma |
| BBC5. | [M]Granulosa cell-theca cell tumour |
| BBC6. | [M]Androblastoma |
| BBC61 | [M]Androblastoma, malignant |
| BBC6z | [M]Androblastoma NOS |
| BBc8. | [M]Pacinian tumour |
| BBc9. | [M]Retinoblastomas |
| BBCA. | [M]Sertoli cell carcinoma |
| BBCB. | [M]Tubular androblastoma with lipid storage |
| BBCC. | [M]Leydig cell tumour |
| BBcD. | [M]Aesthesioneuroepithelioma |
| BBCD. | [M]Hilar cell tumour |
| BBCG. | [M]Sclerosing stromal tumour |
| BBcz. | [M]Neuroepitheliomatous neoplasm NOS |
| BBCz. | [M]Specialised gonadal neoplasm NOS |
| BBd.. | [M]Meningiomas |
| BBd0. | [M]Meningioma NOS |
| BBd1. | [M]Meningiomatosis NOS |
| BBd5. | [M]Psammomatous meningioma |
| BBd7. | [M]Haemangioblastic meningioma |
| BBd8. | [M]Haemangiopericytic meningioma |
| BBD8. | [M]Extra-adrenal paraganglioma, malignant |
| BBd9. | [M]Transitional meningioma |
| BBDA. | [M]Phaeochromocytoma, malignant |
| BBdB. | [M]Meningeal sarcomatosis |
| BBDB. | [M]Glomangiosarcoma |
| BBDC. | [M]Glomus tumour |
| BBDD. | [M]Glomangioma |
| BBDE. | [M]Gangliocytic paraganglioma |
| BBDF. | [M]Glomangiomyoma |
| BBdz. | [M]Meningioma NOS |
| BBDz. | [M]Paraganglioma or glomus tumour NOS |
| BBe.. | [M]Nerve sheath tumour |
| BBE.. | [M]Naevi and melanomas |
| BBe0. | [M]Neurofibroma NOS |
| BBE0. | [M]Pigmented naevus NOS |
| BBe1. | [M]Neurofibromatosis NOS |
| BBE2. | [M]Nodular melanoma |
| BBe3. | [M]Melanotic neurofibroma |
| BBe4. | [M]Plexiform neurofibroma |
| BBe5. | [M]Neurilemmoma NOS |
| BBe6. | [M]Neurinomatosis |
| BBe7. | [M]Neurilemmoma, malignant |
| BBE7. | [M]Neuronaevus |
| BBe8. | [M]Neuroma NOS |
| BBE8. | [M]Magnocellular naevus |
| BBe9. | [M]Triton tumour, malignant |
| BBeA. | [M]Neurothekeoma |
| BBEa. | [M]Spindle cell naevus |
| BBEF. | [M]Hutchinson's melanotic freckle |
| BBez. | [M]Nerve sheath tumour NOS |
| BBf.. | [M]Granular cell tumours and alveolar soft part sarcoma |
| BBF.. | [M]Soft tissue tumours and sarcomas NOS |
| BBf0. | [M]Granular cell tumour NOS |
| BBF0. | [M]Soft tissue tumour, benign |
| BBf1. | [M]Granular cell tumour, malignant |
| BBF1. | [M]Sarcoma NOS |
| BBf2. | [M]Alveolar soft part sarcoma |
| BBF2. | [M]Sarcomatosis NOS |
| BBF3. | [M]Spindle cell sarcoma |
| BBF4. | [M]Giant cell sarcoma (except of bone) |
| BBF5. | [M]Small cell sarcoma |
| BBF6. | [M]Epithelioid cell sarcoma |
| BBfz. | [M]Granular cell tumour or alveolar soft part sarcoma NOS |
| BBFz. | [M]Soft tissue tumour or sarcoma NOS |
| BBg.. | [M]Lymphomas, NOS or diffuse |
| BBG.. | [M]Fibromatous neoplasms |
| BBG1. | [M]Fibrosarcoma NOS |
| BBG2. | [M]Fibromyxoma |
| BBG3. | [M]Fibromyxosarcoma |
| BBg6. | [M]Lymphosarcoma NOS |
| BBG8. | [M]Infantile fibrosarcoma |
| BBG9. | [M]Elastofibroma |
| BBGA. | [M]Aggressive fibromatosis |
| BBGB. | [M]Abdominal fibromatosis |
| BBGC. | [M]Desmoplastic fibroma |
| BBGD. | [M]Fibrous histiocytoma NOS |
| BBGE. | [M]Atypical fibrous histiocytoma |
| BBGF. | [M]Fibrous histiocytoma, malignant |
| BBGG. | [M]Fibroxanthoma NOS |
| BBGH. | [M]Atypical fibroxanthoma |
| BBGJ. | [M]Fibroxanthoma, malignant |
| BBGK. | [M]Dermatofibroma NOS |
| BBGL. | [M]Dermatofibroma protuberans |
| BBGM. | [M]Dermatofibrosarcoma NOS |
| BBgN. | [M]Malign lymphoma,lymphocytic,intermediate differn, diffuse |
| BBgP. | [M]Malignant lymphoma, mixed small and large cell, diffuse |
| BBgQ. | [M]Malignant lymphomatous polyposis |
| BBGz. | [M]Fibromatous neoplasm NOS |
| BBh.. | [M]Reticulosarcomas |
| BBH.. | [M]Myxomatous neoplasms |
| BBh0. | [M]Reticulosarcoma NOS |
| BBH0. | [M]Myxoma NOS |
| BBh1. | [M]Reticulosarcoma, pleomorphic cell type |
| BBH1. | [M]Myxosarcoma |
| BBh2. | [M]Reticulosarcoma, nodular |
| BBhz. | [M]Reticulosarcoma NOS |
| BBHz. | [M]Myxomatous neoplasm NOS |
| BBHZ. | [M]Angiomyxoma |
| BBj.. | [M]Hodgkin's disease |
| BBj1. | [M]Hodgkin's disease, lymphocytic predominance |
| BBJ2. | [M]Fibrolipoma |
| BBj3. | [M]Hodgkin's disease, lymphocytic depletion NOS |
| BBJ4. | [M]Fibromyxolipoma |
| BBj5. | [M]Hodgkin's disease, lymphocytic depletion, reticular type |
| BBj6. | [M]Hodgkin's disease, nodular sclerosis NOS |
| BBj7. | [M]Hodgkin's disease, nodular sclerosis, cellular phase |
| BBj8. | [M]Hodgkin's paragranuloma |
| BBJB. | [M]Angiolipomatous neoplasms |
| BBJB0 | [M]Angiomyolipoma |
| BBJB1 | [M]Angiomyoliposarcoma |
| BBJB2 | [M]Angiolipoma NOS |
| BBJB3 | [M]Angiolipoma, infiltrating |
| BBJBz | [M]Angiolipomatous neoplasm NOS |
| BBJC. | [M]Myelolipoma |
| BBJD. | [M]Hibernoma |
| BBJE. | [M]Lipoblastomatosis |
| BBJF. | [M]Pleomorphic lipoma |
| BBJH. | [M]Dedifferentiated liposarcoma |
| BBjz. | [M]Hodgkin's disease NOS |
| BBk.. | [M]Lymphomas, nodular or follicular |
| BBK0. | [M]Leiomyomatous neoplasms |
| BBK00 | [M]Leiomyoma NOS |
| BBK01 | [M]Intravascular leiomyomatosis |
| BBK02 | [M]Leiomyosarcoma NOS |
| BBK03 | [M]Epithelioid leiomyoma |
| BBK04 | [M]Epithelioid leiomyosarcoma |
| BBK05 | [M]Cellular leiomyoma |
| BBK06 | [M]Bizarre leiomyoma |
| BBK07 | [M]Myxoid leiomyosarcoma |
| BBK0z | [M]Leiomyomatous neoplasm NOS |
| BBK1. | [M]Angiomyomatous neoplasms |
| BBK10 | [M]Angiomyoma |
| BBK11 | [M]Angiomyosarcoma |
| BBK1z | [M]Angiomyomatous neoplasm NOS |
| BBk2. | [M]Malignant lymphoma, centroblastic-centrocytic, follicular |
| BBK20 | [M]Myoma |
| BBK21 | [M]Myosarcoma |
| BBK2z | [M]Myoma or myosarcoma NOS |
| BBk3. | [M]Malig lymphoma, lymphocytic, well differentiated,nodular |
| BBK30 | [M]Rhabdomyoma NOS |
| BBK34 | [M]Fetal rhabdomyoma |
| BBK35 | [M]Adult rhabdomyoma |
| BBK38 | [M]Smooth muscle tumour NOS |
| BBK3z | [M]Rhabdomyomatous neoplasm NOS |
| BBkz. | [M]Lymphoma, nodular or follicular NOS |
| BBL.. | [M]Complex mixed and stromal neoplasms |
| BBl1. | [M]Sezary's disease |
| BBL1. | [M]Endolymphatic stromal myosis |
| BBL2. | [M]Adenomyoma |
| BBL3. | [M]Pleomorphic adenoma |
| BBL4. | [M]Mixed tumour, malignant, NOS |
| BBL5. | [M]Mullerian mixed tumour |
| BBL6. | [M]Mesodermal mixed tumour |
| BBL70 | [M]Mesoblastic nephroma |
| BBL71 | [M]Nephroblastoma NOS |
| BBL72 | [M]Epithelial nephroblastoma |
| BBL73 | [M]Mesenchymal nephroblastoma |
| BBL9. | [M]Carcinosarcoma NOS |
| BBLA. | [M]Carcinosarcoma, embryonal type |
| BBLB. | [M]Myoepithelioma |
| BBLC. | [M]Mesenchymomas |
| BBLC0 | [M]Mesenchymoma, benign |
| BBLC1 | [M]Mesenchymoma, malignant |
| BBLCz | [M]Mesenchymoma NOS |
| BBLD. | [M]Embryonal sarcoma |
| BBLE. | [M]Adenosarcoma |
| BBLG. | [M]Carcinoma in pleomorphic adenoma |
| BBLH. | [M]Rhabdoid sarcoma |
| BBLJ. | [M]Clear cell sarcoma of kidney |
| BBLz. | [M]Complex mixed or stromal neoplasm NOS |
| BBm.. | [M]Miscellaneous reticuloendothelial neoplasms |
| BBM.. | [M]Fibroepithelial neoplasms |
| BBm0. | [M]Microglioma |
| BBM01 | [M]Brenner tumour, malignant |
| BBM1. | [M]Fibroadenoma NOS |
| BBm2. | [M]Histiocytic medullary reticulosis |
| BBM2. | [M]Intracanalicular fibroadenoma NOS |
| BBm3. | [M]Letterer - Siwe disease |
| BBM3. | [M]Pericanalicular fibroadenoma |
| BBM4. | [M]Adenofibroma NOS |
| BBm5. | [M] Peripheral T-cell lymphoma NOS |
| BBm6. | [M] Alpha heavy chain disease |
| BBm7. | [M] Monoclonal gammopathy |
| BBm8. | [M] Angioimmunoblastic lymphadenopathy |
| BBm9. | [M] Monocytoid B-cell lymphoma |
| BBMA. | [M]Juvenile fibroadenoma |
| BBMB. | [M]Giant fibroadenoma |
| BBmJ. | [M] Angioendotheliomatosis |
| BBMz. | [M]Fibroepithelial neoplasm NOS |
| BBn.. | [M]Plasma cell tumours |
| BBN.. | [M]Synovial neoplasms |
| BBn0. | [M]Plasma cell myeloma |
| BBN4. | [M]Synovial sarcoma, biphasic type |
| BBN5. | [M]Clear cell sarcoma of tendons and aponeuroses |
| BBnz. | [M]Plasma cell tumour NOS |
| BBp.. | [M]Mast cell tumours |
| BBP.. | [M]Mesothelial neoplasms |
| BBP0. | [M]Mesothelioma, benign |
| BBP1. | [M]Mesothelioma, malignant |
| BBp2. | [M]Malignant mastocytosis |
| BBP8. | [M]Adenomatoid tumour NOS |
| BBP9. | [M]Cystic mesothelioma |
| BBPX. | [M]Mesothelioma, unspecified |
| BBpz. | [M]Mast cell tumour NOS |
| BBPz. | [M]Mesothelial neoplasm NOS |
| BBq.. | [M]Burkitt's tumours |
| BBq0. | [M]Burkitt's tumour |
| BBQ2. | [M]Germinoma |
| BBQ3. | [M]Embryonal carcinoma NOS |
| BBQ4. | [M]Endodermal sinus tumour |
| BBQ5. | [M]Polyembryoma |
| BBQ6. | [M]Gonadoblastoma |
| BBQ7. | [M]Teratomas |
| BBQ71 | [M]Teratoma NOS |
| BBQ72 | [M]Teratoma, malignant, NOS |
| BBQ73 | [M]Teratocarcinoma |
| BBQ74 | [M]Malignant teratoma, undifferentiated type |
| BBQ75 | [M]Malignant teratoma, intermediate type |
| BBQ7z | [M]Teratoma NOS |
| BBQA. | [M]Strumal neoplasms |
| BBQA0 | [M]Struma ovarii NOS |
| BBQA2 | [M]Strumal carcinoid |
| BBQAz | [M]Strumal neoplasm NOS |
| BBQB. | [M]Mixed germ cell tumour |
| BBqz. | [M]Burkitt's tumour NOS |
| BBrA3 | [M]Myeloid sarcoma |
| BBS.. | [M]Mesonephromas |
| BBS0. | [M]Mesonephroma, benign |
| BBs1. | [M]Acute panmyelosis |
| BBs2. | [M]Chronic myeloproliferative disease |
| BBs3. | [M]Myelosclerosis with myeloid metaplasia |
| BBS3. | [M]Endosalpingioma |
| BBSz. | [M]Mesonephroma NOS |
| BBT0. | [M]Haemangioma NOS |
| BBT1. | [M]Haemangiosarcoma |
| BBT2. | [M]Cavernous haemangioma |
| BBT3. | [M]Venous haemangioma |
| BBT4. | [M]Racemose haemangioma |
| BBT5. | [M]Kupffer cell sarcoma |
| BBT7. | [M]Haemangioendothelioma |
| BBT70 | [M]Haemangioendothelioma, benign |
| BBT71 | [M]Haemangioendothelioma, malignant |
| BBT7z | [M]Haemangioendothelioma NOS |
| BBT8. | [M]Capillary haemangioma |
| BBT9. | [M]Intramuscular haemangioma |
| BBTA. | [M]Kaposi's sarcoma |
| BBTB. | [M]Angiokeratoma |
| BBTC. | [M]Verrucous keratotic haemangioma |
| BBTD. | [M]Haemangiopericytic neoplasms |
| BBTD0 | [M]Haemangiopericytoma, benign |
| BBTD1 | [M]Haemangiopericytoma NOS |
| BBTD2 | [M]Haemangiopericytoma, malignant |
| BBTDz | [M]Haemangiopericytic neoplasm NOS |
| BBTE. | [M]Angiofibroma NOS |
| BBTF. | [M]Haemangioblastoma |
| BBTG. | [M]Epithelioid haemangioma |
| BBTH. | [M]Histiocytoid haemangioma |
| BBTJ. | [M]Epithelioid haemangioendothelioma NOS |
| BBTK. | [M]Epithelioid haemangioendothelioma, malignant |
| BBTL. | [M]Intravascular bronchial alveolar tumour |
| BBTz. | [M]Blood vessel tumour NOS |
| BBU.. | [M]Lymphatic vessel tumours |
| BBU0. | [M]Lymphangioma NOS |
| BBU1. | [M]Lymphangiosarcoma |
| BBU2. | [M]Capillary lymphangioma |
| BBU3. | [M]Cavernous lymphangioma |
| BBU4. | [M]Cystic lymphangioma |
| BBU5. | [M]Lymphangiomyoma |
| BBU6. | [M]Lymphangiomyomatosis |
| BBU7. | [M]Haemolymphangioma |
| BBUz. | [M]Lymphatic vessel tumour NOS |
| BBV.. | [M]Osteomas and osteosarcomas |
| BBV1. | [M]Osteosarcoma NOS |
| BBVz. | [M]Osteoma or osteosarcoma NOS |
| BBW.. | [M]Chondromatous neoplasms |
| BBW0. | [M]Osteochondroma |
| BBW1. | [M]Osteochondromatosis NOS |
| BBW2. | [M]Chondroma NOS |
| BBW3. | [M]Chondromatosis NOS |
| BBW4. | [M]Chondrosarcoma NOS |
| BBW5. | [M]Juxtacortical chondroma |
| BBW6. | [M]Juxtacortical chondrosarcoma |
| BBW7. | [M]Chondroblastoma NOS |
| BBWA. | [M]Chondromyxoid fibroma |
| BBWz. | [M]Chondromatous neoplasm NOS |
| BBX.. | [M]Giant cell tumours |
| BBX0. | [M]Giant cell tumour of bone NOS |
| BBX2. | [M]Giant cell tumour of soft parts NOS |
| BBX3. | [M]Malignant giant cell tumour of soft parts |
| BBXz. | [M]Giant cell tumour NOS |
| BBY0. | [M]Ewing's sarcoma |
| BBy1. | [M]No microscopic confirmation of tumour, clinically malig |
| BBY1. | [M]Adamantinoma of long bones |
| BBY2. | [M]Ossifying fibroma |
| BByz. | [M]No microscopic confirmation of tumour, clinically NOS |
| BBYz. | [M]Miscellaneous bone tumour NOS |
| BBz.. | [M]Neoplasm morphology NOS |
| BBZ.. | [M]Odontogenic tumours |
| BBZ1. | [M]Odontogenic tumour NOS |
| BBZ2. | [M]Odontogenic tumour, malignant |
| BBZ3. | [M]Dentinoma |
| BBZ4. | [M]Cementoma NOS |
| BBZ6. | [M]Cementifying fibroma |
| BBZ7. | [M]Gigantiform cementoma |
| BBZ8. | [M]Odontoma NOS |
| BBZ9. | [M]Compound odontoma |
| BBZA. | [M]Complex odontoma |
| BBZC. | [M]Ameloblastic odontosarcoma |
| BBZD. | [M]Adenomatoid odontogenic tumour |
| BBZE. | [M]Calcifying odontogenic cyst |
| BBZF. | [M]Ameloblastoma NOS |
| BBZJ. | [M]Squamous odontogenic tumour |
| BBZK. | [M]Odontogenic myxoma |
| BBZL. | [M]Odontogenic fibroma NOS |
| BBZM. | [M]Ameloblastic fibroma |
| BBZN. | [M]Ameloblastic fibrosarcoma |
| BBZP. | [M]Calcifying epithelial odontogenic tumour |
| BBZz. | [M]Odontogenic tumour NOS |
| By... | Neoplasms otherwise specified |
| Byu.. | [X]Additional neoplasm classification terms |
| ByuC. | [X]Malignant neoplasm of ill-defined, secondary and unspeci |
| ByuC0 | [X]Malignant neoplasm of other specified sites |
| ByuC1 | [X]Malignant neoplasm/overlap lesion/other+ill-defined sites |
| ByuC2 | [X]2ndry+unspcf malignant neoplasm lymph nodes/multi regions |
| ByuC3 | [X]Secondary malignant neoplasm/oth+unspc respiratory organs |
| ByuC4 | [X]Secondary malignant neoplasm/oth+unspcfd digestive organs |
| ByuC5 | [X]2ndry malignant neoplasm/bladder+oth+unsp urinary organs |
| ByuC6 | [X]2ndry malignant neoplasm/oth+unspec parts/nervous system |
| ByuC7 | [X]Secondary malignant neoplasm of other specified sites |
| ByuC8 | [X]Malignant neoplasm without specification of site |
| ByuE. | [X]Malignant neoplasms/independent (primary) multiple sites |
| ByuE0 | [X]Malignant neoplasms/independent(primary)multiple sites |
| BBb.. | [M]Gliomas |
| BBb0. | [M]Glioma, malignant |
| BBb1. | [M]Gliomatosis cerebri |
| BBb2. | [M]Mixed glioma |
| BBb3. | [M]Subependymal glioma |
| BBbC. | [M]Astrocytoma, anaplastic type |
| BBbR. | [M]Oligodendroglioma, anaplastic type |
| BBbT. | [M]Medulloblastoma NOS |
| BBbU. | [M]Desmoplastic medulloblastoma |
| BBbV. | [M]Medullomyoblastoma |
| BBbW. | [M]Cerebellar sarcoma NOS |
| BBbX. | [M]Monstrocellular sarcoma |
| BBbZ. | [M]Pleomorphic xanthoastrocytoma |
| BBD2. | [M]Sympathetic paraganglioma |
| ByuA0 | [X]Malignant neoplasm/other and unspecified cranial nerves |
| ByuA1 | [X]Malignant neoplasm/central nervous system, unspecified |
| ByuA2 | [X]Malignant neoplasm of meninges, unspecified |
| ByuA3 | [X]Malig neopl, overlap lesion brain & other part of CNS |
| BBcA. | [M]Olfactory neurogenic tumour |
| BBCC1 | [M]Leydig cell tumour, malignant |
| BBQ1. | [M]Seminomas |
| BBQ10 | [M]Seminoma, anaplastic type |
| BBQ11 | [M]Spermatocytic seminoma |
| BBQ1z | [M]Seminoma NOS |
| Byu8. | [X]Malignant neoplasm of male genital organs |
| Byu80 | [X]Malignant neoplasm/other specified male genital organs |
| Byu81 | [X]Malignant neoplasm/overlapping lesion/male genital organs |
| Byu82 | [X]Malignant neoplasm of male genital organ, unspecified |
| BBQA1 | [M]Struma ovarii, malignant |
| Byu7. | [X]Malignant neoplasm of female genital organs |
| Byu70 | [X]Malignant neoplasm of uterine adnexa, unspecified |
| Byu71 | [X]Malignant neoplasm/other specified female genital organs |
| Byu72 | [X]Malignant neoplasm/overlapping lesion/feml genital organs |
| Byu73 | [X]Malignant neoplasm of female genital organ, unspecified |
| BB5j2 | [M]Endometrioid carcinoma |
| BB5j5 | [M]Endometrioid adenofibroma, malignant |
| BBL0. | [M]Endometrial stromal sarcoma |
| BBR2. | [M]Choriocarcinoma |
| BBR3. | [M]Choriocarcinoma combined with teratoma |
| BBcB. | [M]Aesthesioneurocytoma |
| BBG7. | [M]Fascial fibrosarcoma |
| BBK31 | [M]Rhabdomyosarcoma NOS |
| BBK32 | [M]Pleomorphic rhabdomyosarcoma |
| BBK33 | [M]Mixed cell rhabdomyosarcoma |
| BBK36 | [M]Embryonal rhabdomyosarcoma |
| BBK37 | [M]Alveolar rhabdomyosarcoma |
| Byu5. | [X]Malignant neoplasm of mesothelial and soft tissue |
| Byu50 | [X]Mesothelioma of other sites |
| Byu51 | [X]Mesothelioma, unspecified |
| Byu52 | [X]Kaposi's sarcoma of multiple organs |
| Byu53 | [X]Kaposi's sarcoma, unspecified |
| Byu54 | [X]Malignant neoplasm/peripheral nerves of trunk,unspecified |
| Byu55 | [X]Mal neoplasm/overlap les/periph nerv+autonomic nerv systm |
| Byu56 | [X]Mal neoplasm/periph nerves+autonomic nervous system,unspc |
| Byu57 | [X]Malignant neoplasm of peritoneum, unspecified |
| Byu58 | [X]Mal neoplasm/connective+soft tissue of trunk,unspecified |
| Byu59 | [X]Malignant neoplasm/connective + soft tissue,unspecified |
| Byu5B | [X]Kaposi's sarcoma of other sites |
| BB601 | [M]Skin appendage carcinoma |
| BB612 | [M]Sweat gland adenocarcinoma |
| BB691 | [M]Sebaceous adenocarcinoma |
| BB6z. | [M]Adnexal and skin appendage neoplasm NOS |
| BBEV. | [M]Blue naevus, malignant |
| BBl.. | [M]Mycosis fungoides |
| BBl0. | [M]Mycosis fungoides |
| BBlz. | [M]Mycosis fungoides NOS |
| BBQ9. | [M]Dermoid cyst with malignant transformation |
| Byu4. | [X]Melanoma and other malignant neoplasms of skin |
| Byu42 | [X]Oth malignant neoplasm/skin of oth+unspecfd parts of face |
| Byu43 | [X]Malignant neoplasm of skin, unspecified |
| Byu5A | [X]Malignant neoplasm overlapping lesion of skin |
| BBE1. | [M]Malignant melanoma NOS |
| BBE10 | [M]Malignant melanoma, regressing |
| BBE11 | [M]Desmoplastic melanoma, malignant |
| BBe2. | [M]Neurofibrosarcoma |
| BBE4. | [M]Balloon cell melanoma |
| BBEA. | [M]Amelanotic melanoma |
| BBEC. | [M]Malignant melanoma in junctional naevus |
| BBEE. | [M]Malignant melanoma in precancerous melanosis |
| BBEG. | [M]Malignant melanoma in Hutchinson's melanotic freckle |
| BBEG0 | [M]Acral lentiginous melanoma, malignant |
| BBEH. | [M]Superficial spreading melanoma |
| BBEM. | [M]Malignant melanoma in giant pigmented naevus |
| BBEP. | [M]Epithelioid cell melanoma |
| BBEQ. | [M]Spindle cell melanoma NOS |
| BBER. | [M]Spindle cell melanoma, type A |
| BBES. | [M]Spindle cell melanoma, type B |
| BBET. | [M]Mixed epithelioid and spindle melanoma |
| BBEX. | [M]Melanoma in situ |
| Byu40 | [X]Malignant melanoma of other+unspecified parts of face |
| Byu41 | [X]Malignant melanoma of skin, unspecified |
| BBg5. | [M]Malignant lymphoma, convoluted cell type NOS |
| BBn1. | [M]Plasma cell tumour, benign |
| BBn2. | [M]Plasmacytoma NOS |
| BBn3. | [M]Plasma cell tumour, malignant |
| BBv1. | [M]Angioendotheliomatosis |
| BBv2. | [M]AngiocentricT-cell lymphoma |
| BBV3. | [M]Fibroblastic osteosarcoma |
| BBV4. | [M]Telangiectatic osteosarcoma |
| BBV5. | [M]Osteosarcoma in Paget's disease of bone |
| BBV6. | [M]Juxtacortical osteosarcoma |
| BBV8. | [M]Osteoblastoma |
| BBV9. | [M]Myxoid chondrosarcoma |
| BBVA. | [M] Small cell osteosarcoma |
| BBW8. | [M]Chondroblastoma, malignant |
| BBW9. | [M]Mesenchymal chondrosarcoma |
| BBX1. | [M]Giant cell tumour of bone, malignant |
| Byu3. | [X]Malignant neoplasm of bone and articular cartilage |
| Byu30 | [X]Mal neoplasm/overlap lesion/bone+articular cartilage/limb |
| Byu31 | [X]Malignant neoplasm/bones+articular cartilage/limb,unspfd |
| Byu32 | [X]Malignant neoplasm/overlap lesion/bone+articulr cartilage |
| Byu33 | [X]Malignant neoplasm/bone+articular cartilage, unspecified |
| BBp1. | [M]Mast cell sarcoma |
| BBP3. | [M]Fibrous mesothelioma, malignant |
| BBP5. | [M]Epithelioid mesothelioma, malignant |
| BBP7. | [M]Mesothelioma, biphasic type, malignant |
| Byu2. | [X]Malignant neoplasm of respiratory and intrathoracic orga |
| Byu21 | [X]Malignant neoplasm/overlap lesion/heart,mediastinm+pleura |
| Byu22 | [X]Malignant neoplasm/upper resp tract, part unspecified |
| Byu23 | [X]Malignant neopl/overlapping les/resp+intrathoracic organs |
| Byu24 | [X]Malignant neoplasm/ill-defined sites within resp system |
| Byu25 | [X]Malignant neoplasm of mediastinum, part unspecified |
| BB5S2 | [M]Bronchiolo-alveolar adenocarcinoma |
| BB5S4 | [M]Alveolar adenocarcinoma |
| Byu20 | [X]Malignant neoplasm of bronchus or lung, unspecified |
| Byu1. | [X]Malignant neoplasm of digestive organs |
| Byu12 | [X]Malignant neoplasm of intestinal tract, part unspecified |
| Byu13 | [X]Malignant neoplsm/ill-defin sites within digestive system |
| BB5B1 | [M]Islet cell carcinoma |
| BB5B2 | [M]Insulinoma NOS |
| BB5B3 | [M]Insulinoma, malignant |
| BB5B5 | [M]Glucagonoma, malignant |
| BB5B6 | [M]Mixed islet cell and exocrine adenocarcinoma |
| BB5D. | [M]Hepatobiliary tract adenomas and carcinomas |
| BB5D0 | [M]Bile duct adenoma |
| BB5D1 | [M]Cholangiocarcinoma |
| BB5D3 | [M]Bile duct cystadenocarcinoma |
| BB5D5 | [M]Hepatocellular carcinoma NOS |
| BB5D7 | [M]Combined hepatocellular carcinoma and cholangiocarcinoma |
| BB5D8 | [M]Hepatocellular carcinoma, fibrolamellar |
| BB5Dz | [M]Hepatobiliary adenoma or carcinoma NOS |
| Byu10 | [X]Other sarcomas of the liver |
| Byu11 | [X]Other specified carcinomas of liver |
| BB5N. | [M]Adenomatous and adenocarcinomatous polyps of colon |
| BB5N1 | [M]Adenocarcinoma in adenomatous polposis coli |
| BB5C1 | [M]Gastrinoma, malignant |
| BB5Cz | [M]Gastrinoma or carcinoma NOS |
| BBZG. | [M]Ameloblastoma, malignant |
| BBZH. | [M]Odontoameloblastoma |
| Byu0. | [X]Malignant neoplasm of lip, oral cavity and pharynx |
| 13Y8. | Alcoholics anonymous |
| 1462 | H/O: alcoholism |
| 1B1c. | Alcohol induced hallucinations |
| 66e.. | Alcohol disorder monitoring |
| 66e0. | Alcohol abuse monitoring |
| 8BA8. | Alcohol detoxification |
| 8H35. | Admitted to alcohol detoxification centre |
| 8H7p. | Referral to community alcohol team |
| 8HkG. | Referral to specialist alcohol treatment service |
| 8HkJ. | Referral to alcohol brief intervention service |
| 9NN2. | Under care of community alcohol team |
| C1505 | Alcohol-induced pseudo-Cushing's syndrome |
| E01.. | Alcoholic psychoses |
| E010. | Alcohol withdrawal delirium |
| E011. | Alcohol amnestic syndrome |
| E0110 | Korsakov's alcoholic psychosis |
| E0111 | Korsakov's alcoholic psychosis with peripheral neuritis |
| E011z | Alcohol amnestic syndrome NOS |
| E012. | Other alcoholic dementia |
| E0120 | Chronic alcoholic brain syndrome |
| E013. | Alcohol withdrawal hallucinosis |
| E014. | Pathological alcohol intoxication |
| E015. | Alcoholic paranoia |
| E01y. | Other alcoholic psychosis |
| E01y0 | Alcohol withdrawal syndrome |
| E01yz | Other alcoholic psychosis NOS |
| E01z. | Alcoholic psychosis NOS |
| E23.. | Alcohol dependence syndrome |
| E230. | Acute alcoholic intoxication in alcoholism |
| E2300 | Acute alcoholic intoxication, unspecified, in alcoholism |
| E2301 | Continuous acute alcoholic intoxication in alcoholism |
| E2302 | Episodic acute alcoholic intoxication in alcoholism |
| E2303 | Acute alcoholic intoxication in remission, in alcoholism |
| E230z | Acute alcoholic intoxication in alcoholism NOS |
| E231. | Chronic alcoholism |
| E2310 | Unspecified chronic alcoholism |
| E2311 | Continuous chronic alcoholism |
| E2312 | Episodic chronic alcoholism |
| E2313 | Chronic alcoholism in remission |
| E231z | Chronic alcoholism NOS |
| E23z. | Alcohol dependence syndrome NOS |
| Eu101 | [X]Mental and behav dis due to use of alcohol: harmful use |
| Eu102 | [X]Mental and behav dis due to use alcohol: dependence syndr |
| Eu103 | [X]Mental and behav dis due to use alcohol: withdrawal state |
| Eu104 | [X]Men & behav dis due alcohl: withdrawl state with delirium |
| Eu105 | [X]Mental & behav dis due to use alcohol: psychotic disorder |
| Eu106 | [X]Mental and behav dis due to use alcohol: amnesic syndrome |
| Eu107 | [X]Men & behav dis due alcoh: resid & late-onset psychot dis |
| Eu108 | [X]Alcohol withdrawal-induced seizure |
| F11x0 | Cerebral degeneration due to alcoholism |
| F1440 | Cerebellar ataxia due to alcoholism |
| F25B. | Alcohol-induced epilepsy |
| F375. | Alcoholic polyneuropathy |
| F3941 | Alcoholic myopathy |
| G555. | Alcoholic cardiomyopathy |
| J153. | Alcoholic gastritis |
| J610. | Alcoholic fatty liver |
| J611. | Acute alcoholic hepatitis |
| J612. | Alcoholic cirrhosis of liver |
| J6120 | Alcoholic fibrosis and sclerosis of liver |
| J613. | Alcoholic liver damage unspecified |
| J6130 | Alcoholic hepatic failure |
| J617. | Alcoholic hepatitis |
| J6170 | Chronic alcoholic hepatitis |
| J6710 | Alcohol-induced chronic pancreatitis |
| Z1911 | Alcohol withdrawal regime |
| Z4B1. | Alcoholism counselling |
| 13c.. | Drug user |
| 13c0. | Injecting drug user |
| 13c1. | Intravenous drug user |
| 13c2. | Never injecting drug user |
| 13c3. | Intramuscular drug user |
| 13c4. | Intranasal drug user |
| 13c5. | Substance misuse increased |
| 13c6. | Substance misuse decreased |
| 13c7. | Current drug user |
| 13c8. | Reduced drugs misuse |
| 13c9. | Subcutaneous drug user |
| 13cA. | Smokes drugs |
| 13cB. | Misuses drugs orally |
| 13cC. | Continuous use of drugs |
| 13cD. | Episodic use of drugs |
| 13cE. | Prolonged high dose use of cannabis |
| 13cH. | Persistent substance misuse |
| 13cK. | Current non recreational drug user |
| 13cM. | Substance misuse |
| 13cN. | Has never shared drug injection equipment |
| 13cQ. | Behavioural tolerance to drug |
| 13cR. | Physical tolerance to drug |
| 13cS. | Psychological tolerance to drug |
| 13cT. | Reverse tolerance to drug |
| 1B1c. | Alcohol induced hallucinations |
| 1P30. | Compulsive uncontrollable drug taking |
| 1P31. | Compulsive drug taking |
| 1P6.. | Craving for drugs |
| 1P60. | Craves for drugs |
| 1P62. | Abnormal craving for drugs |
| 1P63. | Excessive craving for drugs |
| 1P64. | Irresistible craving for drugs |
| 1TE.. | Uses heroin on top of substitution therapy |
| 1TF.. | Does not use heroin on top of substitution therapy |
| 68U.. | Drugs of abuse screening |
| 68U0. | Drugs of abuse urine screening |
| 8AA.. | Drug abuse monitoring |
| 8B23. | Drug addiction therapy |
| 8B2N. | Drug addiction detoxification therapy - methadone |
| 8B2P. | Drug addiction maintenance therapy - methadone |
| 8B2Q. | Drug addiction maintenance therapy - buprenorphine |
| 8B2R. | Drug addiction detoxification therapy - buprenorphine |
| 8B2S. | Opioid agonist substitution therapy |
| 8B2T. | Opioid antagonist therapy |
| 8BA9. | Detoxification dependence drug |
| 8BAd. | Opiate dependence detoxification |
| 8BE0. | Reinduction to methadone maintenance therapy |
| 8BE1. | Reinduction to buprenorphine maintenance therapy |
| 8FB.. | Drug rehabilitation |
| 8FB0. | Drug detoxification programme completed |
| 9HC.. | Substance misuse monitoring |
| 9HC0. | Initial substance misuse assessment |
| 9HC1. | Follow up substance misuse assessment |
| E02.. | Drug psychoses |
| E020. | Drug withdrawal syndrome |
| E021. | Drug-induced paranoia or hallucinatory states |
| E0210 | Drug-induced paranoid state |
| E0211 | Drug-induced hallucinosis |
| E021z | Drug-induced paranoia or hallucinatory state NOS |
| E022. | Pathological drug intoxication |
| E02y. | Other drug psychoses |
| E02y0 | Drug-induced delirium |
| E02y1 | Drug-induced dementia |
| E02y2 | Drug-induced amnestic syndrome |
| E02y3 | Drug-induced depressive state |
| E02y4 | Drug-induced personality disorder |
| E02yz | Other drug psychoses NOS |
| E02z. | Drug psychosis NOS |
| E24.. | Drug dependence |
| E240. | Opioid type drug dependence |
| E2400 | Unspecified opioid dependence |
| E2401 | Continuous opioid dependence |
| E2402 | Episodic opioid dependence |
| E2403 | Opioid dependence in remission |
| E240z | Opioid drug dependence NOS |
| E241. | Hypnotic or anxiolytic dependence |
| E2410 | Hypnotic or anxiolytic dependence, unspecified |
| E2411 | Hypnotic or anxiolytic dependence, continuous |
| E2412 | Hypnotic or anxiolytic dependence, episodic |
| E2413 | Hypnotic or anxiolytic dependence in remission |
| E241z | Hypnotic or anxiolytic dependence NOS |
| E242. | Cocaine type drug dependence |
| E2420 | Cocaine dependence, unspecified |
| E2421 | Cocaine dependence, continuous |
| E2422 | Cocaine dependence, episodic |
| E2423 | Cocaine dependence in remission |
| E242z | Cocaine drug dependence NOS |
| E243. | Cannabis type drug dependence |
| E2430 | Cannabis dependence, unspecified |
| E2431 | Cannabis dependence, continuous |
| E2432 | Cannabis dependence, episodic |
| E2433 | Cannabis dependence in remission |
| E243z | Cannabis drug dependence NOS |
| E244. | Amphetamine or other psychostimulant dependence |
| E2440 | Amphetamine or psychostimulant dependence, unspecified |
| E2441 | Amphetamine or psychostimulant dependence, continuous |
| E2442 | Amphetamine or psychostimulant dependence, episodic |
| E2443 | Amphetamine or psychostimulant dependence in remission |
| E244z | Amphetamine or psychostimulant dependence NOS |
| E245. | Hallucinogen dependence |
| E2450 | Hallucinogen dependence, unspecified |
| E2451 | Hallucinogen dependence, continuous |
| E2452 | Hallucinogen dependence, episodic |
| E2453 | Hallucinogen dependence in remission |
| E245z | Hallucinogen dependence NOS |
| E246. | Glue sniffing dependence |
| E2460 | Glue sniffing dependence, unspecified |
| E2461 | Glue sniffing dependence, continuous |
| E2462 | Glue sniffing dependence, episodic |
| E2463 | Glue sniffing dependence in remission |
| E246z | Glue sniffing dependence NOS |
| E247. | Other specified drug dependence |
| E2470 | Other specified drug dependence, unspecified |
| E2471 | Other specified drug dependence, continuous |
| E2472 | Other specified drug dependence, episodic |
| E2473 | Other specified drug dependence in remission |
| E247z | Other specified drug dependence NOS |
| E248. | Combined opioid with other drug dependence |
| E2480 | Combined opioid with other drug dependence, unspecified |
| E2481 | Combined opioid with other drug dependence, continuous |
| E2482 | Combined opioid with other drug dependence, episodic |
| E2483 | Combined opioid with other drug dependence in remission |
| E248z | Combined opioid with other drug dependence NOS |
| E249. | Combined drug dependence, excluding opioids |
| E2490 | Combined drug dependence, excluding opioid, unspecified |
| E2491 | Combined drug dependence, excluding opioid, continuous |
| E2492 | Combined drug dependence, excluding opioid, episodic |
| E2493 | Combined drug dependence, excluding opioid, in remission |
| E249z | Combined drug dependence, excluding opioid, NOS |
| E24A. | Ecstasy type drug dependence |
| E24z. | Drug dependence NOS |
| E25.. | Nondependent abuse of drugs |
| E252. | Nondependent cannabis abuse |
| E2520 | Nondependent cannabis abuse, unspecified |
| E2521 | Nondependent cannabis abuse, continuous |
| E2522 | Nondependent cannabis abuse, episodic |
| E2523 | Nondependent cannabis abuse in remission |
| E252z | Nondependent cannabis abuse NOS |
| E253. | Nondependent hallucinogen abuse |
| E2530 | Nondependent hallucinogen abuse, unspecified |
| E2531 | Nondependent hallucinogen abuse, continuous |
| E2532 | Nondependent hallucinogen abuse, episodic |
| E2533 | Nondependent hallucinogen abuse in remission |
| E253z | Nondependent hallucinogen abuse NOS |
| E254. | Nondependent hypnotic or anxiolytic abuse |
| E2540 | Nondependent hypnotic or anxiolytic abuse, unspecified |
| E2541 | Nondependent hypnotic or anxiolytic abuse, continuous |
| E2542 | Nondependent hypnotic or anxiolytic abuse, episodic |
| E2543 | Nondependent hypnotic or anxiolytic abuse in remission |
| E254z | Nondependent hypnotic or anxiolytic abuse NOS |
| E255. | Nondependent opioid abuse |
| E2550 | Nondependent opioid abuse, unspecified |
| E2551 | Nondependent opioid abuse, continuous |
| E2552 | Nondependent opioid abuse, episodic |
| E2553 | Nondependent opioid abuse in remission |
| E255z | Nondependent opioid abuse NOS |
| E256. | Nondependent cocaine abuse |
| E2560 | Nondependent cocaine abuse, unspecified |
| E2561 | Nondependent cocaine abuse, continuous |
| E2562 | Nondependent cocaine abuse, episodic |
| E2563 | Nondependent cocaine abuse in remission |
| E256z | Nondependent cocaine abuse NOS |
| E257. | Nondependent amphetamine or other psychostimulant abuse |
| E2570 | Nondependent amphetamine/psychostimulant abuse, unspecified |
| E2571 | Nondependent amphetamine/psychostimulant abuse, continuous |
| E2572 | Nondependent amphetamine or psychostimulant abuse, episodic |
| E2573 | Nondependent amphetamine/psychostimulant abuse in remission |
| E257z | Nondependent amphetamine or psychostimulant abuse NOS |
| E258. | Nondependent antidepressant type drug abuse |
| E2580 | Nondependent antidepressant type drug abuse, unspecified |
| E2581 | Nondependent antidepressant type drug abuse, continuous |
| E2582 | Nondependent antidepressant type drug abuse, episodic |
| E2583 | Nondependent antidepressant type drug abuse in remission |
| E258z | Nondependent antidepressant type drug abuse NOS |
| E259. | Nondependent mixed drug abuse |
| E2590 | Nondependent mixed drug abuse, unspecified |
| E2591 | Nondependent mixed drug abuse, continuous |
| E2592 | Nondependent mixed drug abuse, episodic |
| E2593 | Nondependent mixed drug abuse in remission |
| E2594 | Misuse of prescription only drugs |
| E259z | Nondependent mixed drug abuse NOS |
| E25y. | Nondependent other drug abuse |
| E25y0 | Nondependent other drug abuse, unspecified |
| E25y1 | Nondependent other drug abuse, continuous |
| E25y2 | Nondependent other drug abuse, episodic |
| E25y3 | Nondependent other drug abuse in remission |
| E25yz | Nondependent other drug abuse NOS |
| E25z. | Misuse of drugs NOS |
| E24.. | Drug dependence |
| E240. | Opioid type drug dependence |
| E2400 | Unspecified opioid dependence |
| E2401 | Continuous opioid dependence |
| E2402 | Episodic opioid dependence |
| E2403 | Opioid dependence in remission |
| E240z | Opioid drug dependence NOS |
| E241. | Hypnotic or anxiolytic dependence |
| E2410 | Hypnotic or anxiolytic dependence, unspecified |
| E2411 | Hypnotic or anxiolytic dependence, continuous |
| E2412 | Hypnotic or anxiolytic dependence, episodic |
| E2413 | Hypnotic or anxiolytic dependence in remission |
| E241z | Hypnotic or anxiolytic dependence NOS |
| E242. | Cocaine type drug dependence |
| E2420 | Cocaine dependence, unspecified |
| E2421 | Cocaine dependence, continuous |
| E2422 | Cocaine dependence, episodic |
| E2423 | Cocaine dependence in remission |
| E242z | Cocaine drug dependence NOS |
| E243. | Cannabis type drug dependence |
| E2430 | Cannabis dependence, unspecified |
| E2431 | Cannabis dependence, continuous |
| E2432 | Cannabis dependence, episodic |
| E2433 | Cannabis dependence in remission |
| E243z | Cannabis drug dependence NOS |
| E244. | Amphetamine or other psychostimulant dependence |
| E2440 | Amphetamine or psychostimulant dependence, unspecified |
| E2441 | Amphetamine or psychostimulant dependence, continuous |
| E2442 | Amphetamine or psychostimulant dependence, episodic |
| E2443 | Amphetamine or psychostimulant dependence in remission |
| E244z | Amphetamine or psychostimulant dependence NOS |
| E245. | Hallucinogen dependence |
| E2450 | Hallucinogen dependence, unspecified |
| E2451 | Hallucinogen dependence, continuous |
| E2452 | Hallucinogen dependence, episodic |
| E2453 | Hallucinogen dependence in remission |
| E245z | Hallucinogen dependence NOS |
| E246. | Glue sniffing dependence |
| E2460 | Glue sniffing dependence, unspecified |
| E2461 | Glue sniffing dependence, continuous |
| E2462 | Glue sniffing dependence, episodic |
| E2463 | Glue sniffing dependence in remission |
| E246z | Glue sniffing dependence NOS |
| E247. | Other specified drug dependence |
| E2470 | Other specified drug dependence, unspecified |
| E2471 | Other specified drug dependence, continuous |
| E2472 | Other specified drug dependence, episodic |
| E2473 | Other specified drug dependence in remission |
| E247z | Other specified drug dependence NOS |
| E248. | Combined opioid with other drug dependence |
| E2480 | Combined opioid with other drug dependence, unspecified |
| E2481 | Combined opioid with other drug dependence, continuous |
| E2482 | Combined opioid with other drug dependence, episodic |
| E2483 | Combined opioid with other drug dependence in remission |
| E248z | Combined opioid with other drug dependence NOS |
| E249. | Combined drug dependence, excluding opioids |
| E2490 | Combined drug dependence, excluding opioid, unspecified |
| E2491 | Combined drug dependence, excluding opioid, continuous |
| E2492 | Combined drug dependence, excluding opioid, episodic |
| E2493 | Combined drug dependence, excluding opioid, in remission |
| E249z | Combined drug dependence, excluding opioid, NOS |
| E24A. | Ecstasy type drug dependence |
| E24z. | Drug dependence NOS |
| E25.. | Nondependent abuse of drugs |
| E252. | Nondependent cannabis abuse |
| E2520 | Nondependent cannabis abuse, unspecified |
| E2521 | Nondependent cannabis abuse, continuous |
| E2522 | Nondependent cannabis abuse, episodic |
| E2523 | Nondependent cannabis abuse in remission |
| E252z | Nondependent cannabis abuse NOS |
| E253. | Nondependent hallucinogen abuse |
| E2530 | Nondependent hallucinogen abuse, unspecified |
| E2531 | Nondependent hallucinogen abuse, continuous |
| E2532 | Nondependent hallucinogen abuse, episodic |
| E2533 | Nondependent hallucinogen abuse in remission |
| E253z | Nondependent hallucinogen abuse NOS |
| E254. | Nondependent hypnotic or anxiolytic abuse |
| E2540 | Nondependent hypnotic or anxiolytic abuse, unspecified |
| E2541 | Nondependent hypnotic or anxiolytic abuse, continuous |
| E2542 | Nondependent hypnotic or anxiolytic abuse, episodic |
| E2543 | Nondependent hypnotic or anxiolytic abuse in remission |
| E254z | Nondependent hypnotic or anxiolytic abuse NOS |
| E255. | Nondependent opioid abuse |
| E2550 | Nondependent opioid abuse, unspecified |
| E2551 | Nondependent opioid abuse, continuous |
| E2552 | Nondependent opioid abuse, episodic |
| E2553 | Nondependent opioid abuse in remission |
| E255z | Nondependent opioid abuse NOS |
| E256. | Nondependent cocaine abuse |
| E2560 | Nondependent cocaine abuse, unspecified |
| E2561 | Nondependent cocaine abuse, continuous |
| E2562 | Nondependent cocaine abuse, episodic |
| E2563 | Nondependent cocaine abuse in remission |
| E256z | Nondependent cocaine abuse NOS |
| E257. | Nondependent amphetamine or other psychostimulant abuse |
| E2570 | Nondependent amphetamine/psychostimulant abuse, unspecified |
| E2571 | Nondependent amphetamine/psychostimulant abuse, continuous |
| E2572 | Nondependent amphetamine or psychostimulant abuse, episodic |
| E2573 | Nondependent amphetamine/psychostimulant abuse in remission |
| E257z | Nondependent amphetamine or psychostimulant abuse NOS |
| E258. | Nondependent antidepressant type drug abuse |
| E2580 | Nondependent antidepressant type drug abuse, unspecified |
| E2581 | Nondependent antidepressant type drug abuse, continuous |
| E2582 | Nondependent antidepressant type drug abuse, episodic |
| E2583 | Nondependent antidepressant type drug abuse in remission |
| E258z | Nondependent antidepressant type drug abuse NOS |
| E259. | Nondependent mixed drug abuse |
| E2590 | Nondependent mixed drug abuse, unspecified |
| E2591 | Nondependent mixed drug abuse, continuous |
| E2592 | Nondependent mixed drug abuse, episodic |
| E2593 | Nondependent mixed drug abuse in remission |
| E2594 | Misuse of prescription only drugs |
| E259z | Nondependent mixed drug abuse NOS |
| E25y. | Nondependent other drug abuse |
| E25y0 | Nondependent other drug abuse, unspecified |
| E25y1 | Nondependent other drug abuse, continuous |
| E25y2 | Nondependent other drug abuse, episodic |
| E25y3 | Nondependent other drug abuse in remission |
| E25yz | Nondependent other drug abuse NOS |
| E25z. | Misuse of drugs NOS |
| Eu1.. | [X]Mental and behavioural disorders due to psychoactive subs |
| Eu11. | [X]Mental and behavioural disorders due to use of opioids |
| Eu110 | [X]Mental & behav dis due to use opioids: acute intoxication |
| Eu111 | [X]Mental and behav dis due to use of opioids: harmful use |
| Eu112 | [X]Mental and behav dis due to use opioids: dependence syndr |
| Eu113 | [X]Mental and behav dis due to use opioids: withdrawal state |
| Eu114 | [X]Men & behav dis due opioid: withdrawl state with delirium |
| Eu115 | [X]Mental & behav dis due to use opioids: psychotic disorder |
| Eu116 | [X]Mental and behav dis due to use opioids: amnesic syndrome |
| Eu117 | [X]Men & beh dis due opioids: resid & late-onset psychot dis |
| Eu11y | [X]Men & behav dis due to use opioids: oth men & behav dis |
| Eu11z | [X]Ment & behav dis due use opioids: unsp ment & behav dis |
| Eu12. | [X]Mental and behavioural disorders due to use cannabinoids |
| Eu120 | [X]Mental & behav dis due cannabinoids: acute intoxication |
| Eu121 | [X]Mental and behav dis due to use cannabinoids: harmful use |
| Eu122 | [X]Mental and behav dis due to cannabinoids: dependence synd |
| Eu123 | [X]Mental and behav dis due cannabinoids: withdrawal state |
| Eu124 | [X]Men & beh dis due cannabinds: withdrwl state wth delirium |
| Eu125 | [X]Mental & behav dis due to cannabinoids: psychotic disordr |
| Eu126 | [X]Mental and behav dis due to use cannabinoids: amnesic syn |
| Eu127 | [X]Mnt/bh dis due cannabinds: resid & late-onset psychot dis |
| Eu12y | [X]Men/behav dis due to use cannabinoids: oth men/behav disd |
| Eu12z | [X]Ment/behav dis due use cannabinoids: unsp ment/behav disd |
| Eu13. | [X]Mental and behavioural dis due use sedatives/hypnotics |
| Eu130 | [X]Mental & behav dis due seds/hypntcs: acute intoxication |
| Eu131 | [X]Mental and behav dis due to use seds/hypntcs: harmful use |
| Eu132 | [X]Mental and behav dis due to seds/hypntcs: dependence synd |
| Eu133 | [X]Mental and behav dis due seds/hypntcs: withdrawal state |
| Eu134 | [X]Men & beh dis due seds/hypns: withdrwl state wth delirium |
| Eu135 | [X]Mental & behav dis due to seds/hypntcs: psychotic disordr |
| Eu136 | [X]Mental and behav dis due to use seds/hypntcs: amnesic syn |
| Eu137 | [X]Mnt/bh dis due seds/hypns: resid & late-onset psychot dis |
| Eu13y | [X]Men/behav dis due to use seds/hypntcs: oth men/behav disd |
| Eu13z | [X]Ment/behav dis due use seds/hypntcs: unsp ment/behav disd |
| Eu14. | [X]Mental and behavioural disorders due to use of cocaine |
| Eu140 | [X]Mental & behav dis due to use cocaine: acute intoxication |
| Eu141 | [X]Mental and behav dis due to use of cocaine: harmful use |
| Eu142 | [X]Mental and behav dis due to use cocaine: dependence syndr |
| Eu143 | [X]Mental and behav dis due to use cocaine: withdrawal state |
| Eu144 | [X]Men & behav dis due cocaine: withdrawl state wth delirium |
| Eu145 | [X]Mental & behav dis due to use cocaine: psychotic disorder |
| Eu146 | [X]Mental and behav dis due to use cocaine: amnesic syndrome |
| Eu147 | [X]Men & beh dis due cocaine: resid & late-onset psychot dis |
| Eu14y | [X]Men & behav dis due to use cocaine: oth men & behav dis |
| Eu14z | [X]Ment & behav dis due use cocaine: unsp ment & behav dis |
| Eu16. | [X]Mental and behavioural disorders due to use hallucinogens |
| Eu160 | [X]Mental & behav dis due hallucinogens: acute intoxicatn |
| Eu161 | [X]Mental and behav dis due to use hallucinogens: harmfl use |
| Eu162 | [X]Mental and behav dis due to hallucinogens: dependence syn |
| Eu163 | [X]Mental and behav dis due hallucinogens: withdrawal state |
| Eu164 | [X]Men & beh dis due hallucngns: withdrwl state wth delirium |
| Eu165 | [X]Mental & behav dis due to hallucinogens: psychotic disord |
| Eu166 | [X]Mental and behav dis due use hallucinogens: amnesic syndr |
| Eu167 | [X]Mnt/bh dis due hallucngns: resid & late-onset psychot dis |
| Eu16y | [X]Men/behav dis due to use hallucinogens: oth men/behav dis |
| Eu16z | [X]Ment/behav dis due use hallucinogens: unsp ment/behav dis |
| Eu18. | [X]Mental & behav disorders due to use of volatile solvents |
| Eu180 | [X]Mental & behav dis due vol solvents: acute intoxication |
| Eu181 | [X]Mental and behav dis due volatile solvents: harmful use |
| Eu182 | [X]Mental and behav dis due to vol solvents: dependence synd |
| Eu183 | [X]Mental and behav dis due vol solvents: withdrawal state |
| Eu184 | [X]Men & beh dis vol solvents: withdrawal state wth delirium |
| Eu185 | [X]Mental & behav dis due to vol solvents: psychotic disordr |
| Eu186 | [X]Mental and behav dis due to use vol solvents: amnesic syn |
| Eu187 | [X]Mnt/bh dis vol solvents: resid & late-onset psychotic dis |
| Eu18y | [X]Men/behav dis due to use vol solvents: oth men/behav disd |
| Eu18z | [X]Ment/behav dis due use vol solvents: unsp ment/behav dis |
| Eu19. | [X]Men & behav disorder multiple drug use/psychoactive subst |
| Eu190 | [X]Mental/behav dis multi drg use/psychoac subs: acute intox |
| Eu191 | [X]Mental and behav dis mlti drg/oth psychoa sbs: harmfl use |
| Eu192 | [X]Mental and behav dis mlti/oth psych sbs: dependence syndr |
| Eu193 | [X]Mental and behav dis mlti/oth psychoa sbs: withdrwl state |
| Eu194 | [X]Mnt/bh dis mlti drg use/oth psy sbs: wthdr state + dlrium |
| Eu195 | [X]Ment/behav dis mlti drug use/oth psyc sbs: psychotc dis |
| Eu196 | [X]Mental/behav dis multi drg use/oth psy sbs: amnesic syndr |
| Eu197 | [X]Men/beh dis mlt drg use/oth subs: resid/late psychot dis |
| Eu19y | [X]Men/beh dis mlt drg use/oth psy sbs: oth men & behav dis |
| Eu19z | [X]Ment/beh dis multi drug use/oth psy sbs unsp mnt/beh dis |
| Eu1A. | [X]Mental and behavioural disorders due use of crack cocaine |
| Eu1A0 | [X]Ment behav dis due use crack cocaine: acute intoxication |
| Eu1A1 | [X]Mental behav disorders due use crack cocaine: harmful use |
| Eu1A2 | [X]Mental behav disorders due use crack cocaine: depend synd |
| Eu1A3 | [X]Mental behav disord due crack cocaine: withdrawal state |
| Eu1A4 | [X]Ment behav dis due crack cocaine: withdraw state delirium |
| Eu1A5 | [X]Mental behav disord due crack cocaine: psychotic disorder |
| Eu1A6 | [X]Men behav disorders due crack cocaine: amnesic syndrome |
| Eu1A7 | [X]Men beh dis due crack cocaine: resid late-onset psych dis |
| Eu1Ay | [X]Ment behav disord due crack cocaine: other ment behav dis |
| Eu1Az | [X]Ment behav dis due crack cocaine: unsp ment and behav dis |
| ZV114 | [V]Personal history of psychoactive substance abuse |
| 14AB. | H/O: TIA |
| 14AK. | H/O: Stroke in last year |
| 14A7. | H/O: CVA/stroke |
| 1M4.. | Central post-stroke pain |
| 662e. | Stroke/CVA annual review |
| 662M. | Stroke monitoring |
| 662o. | Haemorrhagic stroke monitoring |
| 7P242 | Delivery of rehabilitation for stroke |
| 8HBJ. | Stroke / transient ischaemic attack referral |
| G61.. | Intracerebral haemorrhage |
| G610. | Cortical haemorrhage |
| G611. | Internal capsule haemorrhage |
| G612. | Basal nucleus haemorrhage |
| G613. | Cerebellar haemorrhage |
| G614. | Pontine haemorrhage |
| G615. | Bulbar haemorrhage |
| G616. | External capsule haemorrhage |
| G617. | Intracerebral haemorrhage, intraventricular |
| G618. | Intracerebral haemorrhage, multiple localized |
| G61X. | Intracerebral haemorrhage in hemisphere, unspecified |
| G61X0 | Left sided intracerebral haemorrhage, unspecified |
| G61X1 | Right sided intracerebral haemorrhage, unspecified |
| G61z. | Intracerebral haemorrhage NOS |
| G62.. | Other and unspecified intracranial haemorrhage |
| G620. | Extradural haemorrhage - nontraumatic |
| G621. | Subdural haemorrhage - nontraumatic |
| G622. | Subdural haematoma - nontraumatic |
| G623. | Subdural haemorrhage NOS |
| G62z. | Intracranial haemorrhage NOS |
| G63y0 | Cerebral infarct due to thrombosis of precerebral arteries |
| G63y1 | Cerebral infarction due to embolism of precerebral arteries |
| G64.. | Cerebral arterial occlusion |
| G6400 | Cerebral infarction due to thrombosis of cerebral arteries |
| G6410 | Cerebral infarction due to embolism of cerebral arteries |
| G64z. | Cerebral infarction NOS |
| G64z0 | Brainstem infarction |
| G64z2 | Left sided cerebral infarction |
| G64z3 | Right sided cerebral infarction |
| G64z4 | Infarction of basal ganglia |
| G65.. | Transient cerebral ischaemia |
| G650. | Basilar artery syndrome |
| G651. | Vertebral artery syndrome |
| G6510 | Vertebro-basilar artery syndrome |
| G652. | Subclavian steal syndrome |
| G653. | Carotid artery syndrome hemispheric |
| G654. | Multiple and bilateral precerebral artery syndromes |
| G656. | Vertebrobasilar insufficiency |
| G65y. | Other transient cerebral ischaemia |
| G65z. | Transient cerebral ischaemia NOS |
| G65z0 | Impending cerebral ischaemia |
| G65z1 | Intermittent cerebral ischaemia |
| G65zz | Transient cerebral ischaemia NOS |
| G66.. | Stroke and cerebrovascular accident unspecified |
| G663. | Brain stem stroke syndrome |
| G664. | Cerebellar stroke syndrome |
| G667. | Left sided CVA |
| G668. | Right sided CVA |
| G669. | Cerebral palsy, not congenital or infantile, acute |
| G6760 | Cereb infarct due cerebral venous thrombosis, nonpyogenic |
| G6W.. | Cereb infarct due unsp occlus/stenos precerebr arteries |
| G6X.. | Cerebrl infarctn due/unspcf occlusn or sten/cerebrl artrs |
| Gyu62 | [X]Other intracerebral haemorrhage |
| Gyu63 | [X]Cerebrl infarctn due/unspcf occlusn or sten/cerebrl artrs |
| Gyu64 | [X]Other cerebral infarction |
| Gyu65 | [X]Occlusion and stenosis of other precerebral arteries |
| Gyu66 | [X]Occlusion and stenosis of other cerebral arteries |
| Gyu6F | [X]Intracerebral haemorrhage in hemisphere, unspecified |
| Gyu6G | [X]Cereb infarct due unsp occlus/stenos precerebr arteries |
| ZV12D | [V]Personal history of transient ischaemic attack |
| Fyu55 | [X]Other transnt cerebral ischaemic attacks+related syndroms |
| 1Z1.. | Chronic renal impairment |
| 1Z10. | Chronic kidney disease stage 1 |
| 1Z11. | Chronic kidney disease stage 2 |
| 1Z12. | Chronic kidney disease stage 3 |
| 1Z13. | Chronic kidney disease stage 4 |
| 1Z14. | Chronic kidney disease stage 5 |
| 1Z15. | Chronic kidney disease stage 3A |
| 1Z16. | Chronic kidney disease stage 3B |
| 1Z17. | Chronic kidney disease stage 1 with proteinuria |
| 1Z18. | Chronic kidney disease stage 1 without proteinuria |
| 1Z19. | Chronic kidney disease stage 2 with proteinuria |
| 1Z1A. | Chronic kidney disease stage 2 without proteinuria |
| 1Z1B. | Chronic kidney disease stage 3 with proteinuria |
| 1Z1C. | Chronic kidney disease stage 3 without proteinuria |
| 1Z1D. | Chronic kidney disease stage 3A with proteinuria |
| 1Z1E. | Chronic kidney disease stage 3A without proteinuria |
| 1Z1F. | Chronic kidney disease stage 3B with proteinuria |
| 1Z1G. | Chronic kidney disease stage 3B without proteinuria |
| 1Z1H. | Chronic kidney disease stage 4 with proteinuria |
| 1Z1J. | Chronic kidney disease stage 4 without proteinuria |
| 1Z1K. | Chronic kidney disease stage 5 with proteinuria |
| 1Z1L. | Chronic kidney disease stage 5 without proteinuria |
| 66i.. | Chronic kidney disease monitoring |
| K07.. | Renal sclerosis unspecified |
| K070. | Atrophy of kidney |
| K071. | Renal fibrosis |
| K072. | Glomerulosclerosis |
| K07z. | Renal sclerosis NOS |
| K08.. | Impaired renal function disorder |
| K080. | Renal osteodystrophy |
| K0800 | Phosphate-losing tubular disorders |
| K0801 | Renal dwarfism |
| K0802 | Renal infantilism |
| K0803 | Renal rickets |
| K080z | Renal osteodystrophy NOS |
| K081. | Nephrogenic diabetes insipidus |
| K08y. | Other impaired renal function disorder |
| K08y0 | Hypokalaemic nephropathy |
| K08y2 | Lightwood - Albright syndrome |
| K08y3 | Renal function impairment with growth failure |
| K08y4 | Renal tubular acidosis |
| K08yz | Other impaired renal function disorder NOS |
| K08z. | Impaired renal function disorder NOS |
| K09.. | Small kidney of unknown cause |
| K090. | Unilateral small kidney |
| K0900 | Atrophy of kidney |
| K091. | Bilateral small kidneys |
| K09z. | Small kidneys unspecified |
| K0y.. | Other specified nephritis, nephrosis or nephrotic syndrome |
| K0z.. | Nephritis, nephrosis and nephrotic syndrome NOS |
| K103. | Pyeloureteritis cystica |
| K13.. | Other kidney and ureter disorders |
| K130. | Nephroptosis |
| K131. | Hypertrophy of kidney |
| K13y. | Other kidney and ureteric disorders |
| K13y0 | Ureteric fistula |
| K13y1 | Adhesions of kidney |
| K13y2 | Adhesions of ureter |
| K13y3 | Periureteritis |
| K13y5 | Polyp of ureter |
| K13y6 | Ureterocele - acquired |
| K13y7 | Megaloureter - acquired |
| K13y9 | Ureteric neuromuscular incoordination |
| K13yz | Other kidney and ureteric disorders NOS |
| K13z. | Kidney and ureter disease NOS |
| Kyu4. | [X]Other disorders of kidney and ureter |
| Kyu40 | [X]Other disorders resulting/impaired renal tubular function |
| Kyu41 | [X]Other specified disorders of kidney and ureter |
| Kyu42 | [X]Oth disordrs/kidney+ureter/infects+parasitic diseases CE |
| Kyu43 | [X]Other disorders of kidney+ureter in other diseases CE |
| J511. | Diverticulitis |
| J5110 | Diverticulitis of the duodenum |
| J5111 | Diverticulitis of the jejunum |
| J5112 | Diverticulitis of the ileum |
| J5113 | Diverticulitis of the small intestine unspecified |
| J5114 | Diverticulitis of the small intestine NOS |
| J5115 | Diverticulitis of the colon |
| J5116 | Diverticulitis of the large intestine unspecified |
| J5117 | Diverticulitis of the large intestine NOS |
| J511y | Diverticulitis unspecified |
| J511z | Diverticulitis NOS |
| J5100 | Diverticulosis of the duodenum |
| J5101 | Diverticulosis of the jejunum |
| J5102 | Diverticulosis of the ileum |
| J5103 | Diverticulosis of the small intestine unspecified |
| J5104 | Diverticulosis of the small intestine NOS |
| J5105 | Diverticulosis of the colon |
| J5106 | Diverticulosis of the large intestine unspecified |
| J5107 | Diverticulosis of the large intestine NOS |
| J5108 | Diverticular disease of both small and large intestine without perforation or abscess |
| J5109 | Bleeding diverticulosis |
| J510y | Diverticulosis unspecified |
| J510z | Diverticulosis NOS |
| J171. | Gastric diverticulum |
| J512. | Perforated diverticulum |
| J5121 | Perforated diverticulum of jejunum |
| J5122 | Perforated diverticulum of ileum |
| J5123 | Perforated diverticulum of small intestine unspecified |
| J5124 | Perforated diverticulum of small intestine NOS |
| J5125 | Perforated diverticulum of colon |
| J5126 | Perforated diverticulum of large intestine unspecified |
| J5127 | Perforated diverticulum of large intestine NOS |
| J5128 | Diverticular disease of both small and large intestine with perforation and abscess |
| J512y | Perforated diverticulum unspecified |
| J512z | Perforated diverticulum of intestine NOS |
| J513. | Diverticular abscess |
| J51z. | Diverticula of the intestine NOS |
| J51.. | Diverticula of intestine |
| X304P | DD - Diverticular disease |
| X304Q | Solitary diverticulum of left side of colon |
| XM0B8 | Simple diverticular disease |
| J5105 | Diverticulosis of the colon |
| X304T | Diverticular disease of right side of colon |
| XM0B9 | Complicated diverticular disease |
| Xa1bK | Diverticulitis of large intestine |
| J5115 | Diverticulitis of colon |
| J5116 | Diverticulitis of the large intestine unspecified |
| J5117 | Diverticulitis of the large intestine NOS |
| Xa1bL | Perforated diverticulum of large intestine |
| J5125 | Perforated diverticulum of colon |
| J5126 | Perforated diverticulum of large intestine unspecified |
| J5127 | Perforated diverticulum of large intestine NOS |
| X304R | Colonic diverticular abscess |
| X304S | Small muscle hypertrophy of sigmoid colon |
| J5109 | Bleeding diverticulosis |
| Xa1d1 | Solitary diverticulum of caecum |
| J5106 | Diverticulosis of the large intestine unspecified |
| J5107 | Diverticulosis of the large intestine NOS |
| J5108 | Diverticular disease of both small and large intestine without perforation or abscess |
| J5128 | Diverticular disease of both small and large intestine with perforation and abscess |
| Xa2ku | Diverticular disease of left side of colon |
| 14AN. | H/O: atrial fibrillation |
| 3272 | ECG: atrial fibrillation |
| 662S. | Atrial fibrillation monitoring |
| 6A9.. | Atrial fibrillation annual review |
| G573. | Atrial fibrillation and flutter |
| G5730 | Atrial fibrillation |
| G5732 | Paroxysmal atrial fibrillation |
| G5733 | Non-rheumatic atrial fibrillation |
| G5734 | Permanent atrial fibrillation |
| G5735 | Persistent atrial fibrillation |
| G573z | Atrial fibrillation and flutter NOS |
| 14NB. | H/O: Peripheral vascular disease procedure |
| G73.. | Other peripheral vascular disease |
| G730. | Raynaud's syndrome |
| G7300 | Raynaud's disease |
| G7301 | Raynaud's phenomenon |
| G730z | Raynaud's syndrome NOS |
| G731. | Thromboangiitis obliterans |
| G7310 | Buerger's disease |
| G7311 | Presenile gangrene |
| G731z | Thromboangiitis obliterans NOS |
| G732. | Peripheral gangrene |
| G7320 | Gangrene of toe |
| G7321 | Gangrene of foot |
| G7322 | Gangrene of finger |
| G7323 | Gangrene of thumb |
| G7324 | Gangrene of hand |
| G733. | Ischaemic foot |
| G73y. | Other specified peripheral vascular disease |
| G73y0 | Diabetic peripheral angiopathy |
| G73y1 | Peripheral angiopathic disease EC NOS |
| G73y2 | Acrocyanosis |
| G73y4 | Acroparaesthesia - Schultze's type |
| G73y5 | Acroparaesthesia - Nothnagel's type |
| G73y6 | Acroparaesthesia - unspecified |
| G73y7 | Erythrocyanosis |
| G73y8 | Erythromelalgia |
| G73yz | Other specified peripheral vascular disease NOS |
| G73z. | Peripheral vascular disease NOS |
| G73z0 | Intermittent claudication |
| G73zz | Peripheral vascular disease NOS |
| Gyu74 | [X]Other specified peripheral vascular diseases |
| P76.. | Other peripheral vascular system anomalies |
| G830. | Varicose veins of the leg with ulcer |
| G831. | Varicose veins of the leg with eczema |
| G832. | Varicose veins of the leg with ulcer and eczema |
| G833. | Varicose veins of the leg with rupture |
| G835. | Infected varicose ulcer |
| G836. | Varicose vein of leg with phlebitis |
| G837. | Venous ulcer of leg |
| G8y1. | Postphlebitic syndrome |
| G8y3. | Chronic peripheral venous hypertension |
| G8yy0 | Chronic venous insufficiency NOS |
| G702. | Extremity artery atheroma |
| G702z | Extremity artery atheroma NOS |
| 14A6. | H/O: heart failure |
| 14AM. | H/O: Heart failure in last year |
| 1O1.. | Heart failure confirmed |
| 662p. | Heart failure 6 month review |
| 662T. | Congestive heart failure monitoring |
| 662W. | Heart failure annual review |
| 8B29. | Cardiac failure therapy |
| 8H2S. | Admit heart failure emergency |
| 9N0k. | Seen in heart failure clinic |
| G1yz1 | Rheumatic left ventricular failure |
| G58.. | Heart failure |
| G580. | Congestive heart failure |
| G5800 | Acute congestive heart failure |
| G5801 | Chronic congestive heart failure |
| G5802 | Decompensated cardiac failure |
| G5803 | Compensated cardiac failure |
| G5804 | Congestive heart failure due to valvular disease |
| G581. | Left ventricular failure |
| G5810 | Acute left ventricular failure |
| G582. | Acute heart failure |
| G583. | Heart failure with normal ejection fraction |
| G58z. | Heart failure NOS |
| G5y4z | Post cardiac operation heart failure NOS |
| L09y2 | Cardiac failure following abortive pregnancy |
| Q48y1 | Congenital cardiac failure |
| 8L51. | Prostatectomy planned |
| A1650 | Tuberculosis of prostate |
| A9812 | Acute gonococcal prostatitis |
| A9832 | Chronic gonococcal prostatitis |
| AD103 | Trichomonal prostatitis |
| B46.. | Malignant neoplasm of prostate |
| B58y5 | Secondary malignant neoplasm of prostate |
| B7C2. | Benign neoplasm of prostate |
| B834. | Carcinoma in situ of prostate |
| B8340 | High grade prostatic intraepithelial neoplasia |
| B915. | Neoplasm of uncertain behaviour of prostate |
| K20.. | Benign prostatic hypertrophy |
| K200. | Prostatic hyperplasia unspecified |
| K201. | Prostatic hyperplasia of the lateral lobe |
| K202. | Prostatic hyperplasia of the medial lobe |
| K20z. | Prostatic hyperplasia NOS |
| K21.. | Prostate inflammatory diseases |
| K210. | Acute prostatitis |
| K211. | Chronic prostatitis |
| K212. | Abscess of prostate |
| K213. | Prostatocystitis |
| K214. | Prostatitis in diseases EC |
| K2140 | Prostatitis in actinomycosis |
| K2141 | Prostatitis in blastomycosis |
| K2142 | Prostatitis in syphilis |
| K2143 | Prostatitis in tuberculosis |
| K2144 | Prostatitis in gonorrhoea |
| K2145 | Prostatitis in moniliasis |
| K2146 | Prostatitis in trichomoniasis |
| K214z | Prostatitis in diseases EC NOS |
| K21y. | Other prostatic inflammatory diseases |
| K21z. | Prostatitis NOS |
| K22.. | Other disorders of prostate |
| K220. | Calculus of prostate |
| K221. | Prostatic congestion or haemorrhage |
| K2210 | Prostatic congestion |
| K2211 | Prostatic haemorrhage |
| K221z | Prostatic congestion or haemorrhage NOS |
| K222. | Atrophy of prostate |
| K22y. | Other disorders of prostate OS |
| K22y0 | Prostatic fistula |
| K22y1 | Infarction of prostate |
| K22y2 | Stricture of prostate |
| K22y3 | Periprostatic adhesions |
| K22yz | Other prostate disorders NOS |
| K22z. | Prostatic disorders NOS |
| Kyu60 | [X]Other inflammatory diseases of prostate |
| Kyu61 | [X]Other specified disorders of prostate |
| Kyu68 | [X]Disorders of prostate in diseases classified elsewhere |
| PCy01 | Congenital absence of prostate |
| PCyx. | Other congenital anomaly of vas deferens or prostate |
| Pyu69 | [X]Oth cong malform vas def/epidid/semin vesicles/prostate |
| 66T1. | Glaucoma monitoring |
| 7275 | Pan retinal photocoagulation for glaucoma |
| F4421 | Glaucomatocyclitic crises |
| F45.. | Glaucoma |
| F450. | Borderline glaucoma |
| F4501 | Open angle glaucoma with borderline intraocular pressure |
| F4502 | Borderline glaucoma with anatomical narrow angle |
| F4503 | Borderline glaucoma steroid responder |
| F450z | Borderline glaucoma NOS |
| F451. | Open-angle glaucoma |
| F4510 | Unspecified open-angle glaucoma |
| F4511 | Primary open-angle glaucoma |
| F4512 | Low tension glaucoma |
| F4513 | Pigmentary glaucoma |
| F4514 | Glaucoma of childhood |
| F4515 | Open-angle glaucoma residual stage |
| F451z | Open-angle glaucoma NOS |
| F452. | Primary angle-closure glaucoma |
| F4520 | Unspecified primary angle-closure glaucoma |
| F4521 | Intermittent primary angle-closure glaucoma |
| F4522 | Acute primary angle-closure glaucoma |
| F4523 | Chronic primary angle-closure glaucoma |
| F4524 | Primary angle-closure glaucoma residual stage |
| F452z | Primary angle-closure glaucoma NOS |
| F453. | Steroid-induced glaucoma |
| F4530 | Steroid-induced glaucoma glaucomatous stage |
| F4531 | Steroid-induced glaucoma residual stage |
| F453z | Steroid-induced glaucoma NOS |
| F454. | Glaucoma due to disease EC |
| F4540 | Glaucoma due to chamber angle anomaly |
| F4541 | Glaucoma due to iris anomaly |
| F4542 | Glaucoma due to other anterior segment anomaly |
| F4543 | Glaucoma due to systemic syndrome |
| F4544 | Glaucoma in endocrine, nutritional and metabolic diseases |
| F454z | Glaucoma due to disease NOS |
| F455. | Glaucoma associated with disorders of the lens |
| F4550 | Phacolytic glaucoma |
| F4551 | Pseudoexfoliation glaucoma |
| F455z | Glaucoma associated with disorders of the lens NOS |
| F456. | Glaucoma associated with other ocular disorders |
| F4560 | Glaucoma due to unspecified ocular disorder |
| F4561 | Glaucoma due to pupillary block |
| F4562 | Glaucoma due to ocular inflammation |
| F4563 | Glaucoma due to ocular vascular disorder |
| F4564 | Glaucoma due to ocular tumour or cyst |
| F4565 | Glaucoma due to ocular trauma |
| F4566 | Neovascular glaucoma |
| F456z | Glaucoma associated with other ocular disorders NOS |
| F45y. | Other specified forms of glaucoma |
| F45y0 | Hypersecretion glaucoma |
| F45y1 | Glaucoma due to episode of increased venous pressure |
| F45y2 | Low tension glaucoma |
| F45yz | Other specified glaucoma NOS |
| F45z. | Glaucoma NOS |
| F4631 | Glaucomatous subcapsular flecks |
| F4H14 | Optic disc glaucomatous atrophy |
| FyuG. | [X]Glaucoma |
| FyuG0 | [X]Other glaucoma |
| FyuG1 | [X]Glaucoma in endocrine,nutritional+metabolic diseases CE |
| FyuG2 | [X]Glaucoma in other diseases classified elsewhere |
| P3200 | Congenital glaucoma |
| Q20y7 | Traumatic glaucoma due to birth trauma |
| 1473 | H/O: epilepsy |
| 1B1W. | Transient epileptic amnesia |
| 1O30. | Epilepsy confirmed |
| 667.. | Epilepsy monitoring |
| 6671 | Initial epilepsy assessment |
| 6672 | Follow-up epilepsy assessment |
| 6673 | Epilepsy associated problems |
| 6674 | Fit frequency |
| 6675 | Last fit |
| 6676 | Epilepsy treatment changed |
| 6677 | Epilepsy treatment started |
| 6678 | Nocturnal epilepsy |
| 6679 | Epilepsy control good |
| 667D. | Epilepsy control poor |
| 667E. | Epilepsy care arrangement |
| 667G. | Epilepsy restricts employment |
| 667H. | Epilepsy prevents employment |
| 667J. | Epilepsy impairs education |
| 667K. | Epilepsy limits activities |
| 667L. | Epilepsy does not limit activities |
| 667M. | Epilepsy management plan given |
| 667N. | Epilepsy severity |
| 667Q. | 1 to 12 seizures a year |
| 667R. | 2 to 4 seizures a month |
| 667S. | 1 to 7 seizures a week |
| 667T. | Daily seizures |
| 667V. | Many seizures a day |
| 667W. | Emergency epilepsy treatment since last appointment |
| 667X. | No epilepsy drug side effects |
| 667Z. | Epilepsy monitoring NOS |
| 8B66. | Anticonvulsant therapy |
| 8BIF. | Epilepsy medication review |
| Eu803 | [X]Acquired aphasia with epilepsy [Landau - Kleffner] |
| F1321 | Progressive myoclonic epilepsy |
| F25.. | Epilepsy |
| F250. | Generalised nonconvulsive epilepsy |
| F2500 | Petit mal (minor) epilepsy |
| F2501 | Pykno-epilepsy |
| F2502 | Epileptic seizures - atonic |
| F2503 | Epileptic seizures - akinetic |
| F2504 | Juvenile absence epilepsy |
| F2505 | Lennox-Gastaut syndrome |
| F250y | Other specified generalised nonconvulsive epilepsy |
| F250z | Generalised nonconvulsive epilepsy NOS |
| F251. | Generalised convulsive epilepsy |
| F2510 | Grand mal (major) epilepsy |
| F2511 | Neonatal myoclonic epilepsy |
| F2512 | Epileptic seizures - clonic |
| F2513 | Epileptic seizures - myoclonic |
| F2514 | Epileptic seizures - tonic |
| F2515 | Tonic-clonic epilepsy |
| F2516 | Grand mal seizure |
| F251y | Other specified generalised convulsive epilepsy |
| F251z | Generalised convulsive epilepsy NOS |
| F252. | Petit mal status |
| F253. | Grand mal status |
| F254. | Partial epilepsy with impairment of consciousness |
| F2540 | Temporal lobe epilepsy |
| F2541 | Psychomotor epilepsy |
| F2542 | Psychosensory epilepsy |
| F2543 | Limbic system epilepsy |
| F2544 | Epileptic automatism |
| F2545 | Complex partial epileptic seizure |
| F254z | Partial epilepsy with impairment of consciousness NOS |
| F255. | Partial epilepsy without impairment of consciousness |
| F2550 | Jacksonian, focal or motor epilepsy |
| F2551 | Sensory induced epilepsy |
| F2552 | Somatosensory epilepsy |
| F2553 | Visceral reflex epilepsy |
| F2554 | Visual reflex epilepsy |
| F2555 | Unilateral epilepsy |
| F2556 | Simple partial epileptic seizure |
| F255y | Partial epilepsy without impairment of consciousness OS |
| F255z | Partial epilepsy without impairment of consciousness NOS |
| F256. | Infantile spasms |
| F2560 | Hypsarrhythmia |
| F2561 | Salaam attacks |
| F256z | Infantile spasms NOS |
| F257. | Kojevnikov's epilepsy |
| F258. | Post-ictal state |
| F259. | Early infant epileptic encephalopathy wth suppression bursts |
| F25A. | Juvenile myoclonic epilepsy |
| F25B. | Alcohol-induced epilepsy |
| F25C. | Drug-induced epilepsy |
| F25D. | Menstrual epilepsy |
| F25E. | Stress-induced epilepsy |
| F25F. | Photosensitive epilepsy |
| F25X. | Status epilepticus, unspecified |
| F25y. | Other forms of epilepsy |
| F25y0 | Cursive (running) epilepsy |
| F25y1 | Gelastic epilepsy |
| F25y2 | Locl-rlt(foc)(part)idiop epilep&epilptic syn seiz locl onset |
| F25y3 | Complex partial status epilepticus |
| F25y4 | Benign Rolandic epilepsy |
| F25y5 | Panayiotopoulos syndrome |
| F25yz | Other forms of epilepsy NOS |
| F25z. | Epilepsy NOS |
| Fyu50 | [X]Other generalized epilepsy and epileptic syndromes |
| Fyu51 | [X]Other epilepsy |
| Fyu52 | [X]Other status epilepticus |
| Fyu59 | [X]Status epilepticus, unspecified |
| SC200 | Traumatic epilepsy |
| 66h.. | Dementia monitoring |
| 6AB.. | Dementia annual review |
| E000. | Uncomplicated senile dementia |
| E001. | Presenile dementia |
| E0010 | Uncomplicated presenile dementia |
| E0011 | Presenile dementia with delirium |
| E0012 | Presenile dementia with paranoia |
| E0013 | Presenile dementia with depression |
| E001z | Presenile dementia NOS |
| E002. | Senile dementia with depressive or paranoid features |
| E0020 | Senile dementia with paranoia |
| E0021 | Senile dementia with depression |
| E002z | Senile dementia with depressive or paranoid features NOS |
| E003. | Senile dementia with delirium |
| E004. | Arteriosclerotic dementia |
| E0040 | Uncomplicated arteriosclerotic dementia |
| E0041 | Arteriosclerotic dementia with delirium |
| E0042 | Arteriosclerotic dementia with paranoia |
| E0043 | Arteriosclerotic dementia with depression |
| E004z | Arteriosclerotic dementia NOS |
| E012. | Other alcoholic dementia |
| E02y1 | Drug-induced dementia |
| E041. | Dementia in conditions EC |
| Eu00. | [X]Dementia in Alzheimer's disease |
| Eu000 | [X]Dementia in Alzheimer's disease with early onset |
| Eu001 | [X]Dementia in Alzheimer's disease with late onset |
| Eu002 | [X]Dementia in Alzheimer's dis, atypical or mixed type |
| Eu00z | [X]Dementia in Alzheimer's disease, unspecified |
| Eu01. | [X]Vascular dementia |
| Eu010 | [X]Vascular dementia of acute onset |
| Eu011 | [X]Multi-infarct dementia |
| Eu012 | [X]Subcortical vascular dementia |
| Eu013 | [X]Mixed cortical and subcortical vascular dementia |
| Eu01y | [X]Other vascular dementia |
| Eu01z | [X]Vascular dementia, unspecified |
| Eu02. | [X]Dementia in other diseases classified elsewhere |
| Eu020 | [X]Dementia in Pick's disease |
| Eu021 | [X]Dementia in Creutzfeldt-Jakob disease |
| Eu022 | [X]Dementia in Huntington's disease |
| Eu023 | [X]Dementia in Parkinson's disease |
| Eu024 | [X]Dementia in human immunodef virus [HIV] disease |
| Eu025 | [X]Lewy body dementia |
| Eu02y | [X]Dementia in other specified diseases classif elsewhere |
| Eu02z | [X] Unspecified dementia |
| Eu041 | [X]Delirium superimposed on dementia |
| F110. | Alzheimer's disease |
| F1100 | Alzheimer's disease with early onset |
| F1101 | Alzheimer's disease with late onset |
| F111. | Pick's disease |
| F112. | Senile degeneration of brain |
| F116. | Lewy body disease |
| Fyu30 | [X]Other Alzheimer's disease |
| 1464 | H/O: schizophrenia |
| E10.. | Schizophrenic disorders |
| E100. | Simple schizophrenia |
| E1000 | Unspecified schizophrenia |
| E1001 | Subchronic schizophrenia |
| E1002 | Chronic schizophrenic |
| E1003 | Acute exacerbation of subchronic schizophrenia |
| E1004 | Acute exacerbation of chronic schizophrenia |
| E1005 | Schizophrenia in remission |
| E100z | Simple schizophrenia NOS |
| E101. | Hebephrenic schizophrenia |
| E1010 | Unspecified hebephrenic schizophrenia |
| E1011 | Subchronic hebephrenic schizophrenia |
| E1012 | Chronic hebephrenic schizophrenia |
| E1013 | Acute exacerbation of subchronic hebephrenic schizophrenia |
| E1014 | Acute exacerbation of chronic hebephrenic schizophrenia |
| E1015 | Hebephrenic schizophrenia in remission |
| E101z | Hebephrenic schizophrenia NOS |
| E102. | Catatonic schizophrenia |
| E1020 | Unspecified catatonic schizophrenia |
| E1021 | Subchronic catatonic schizophrenia |
| E1022 | Chronic catatonic schizophrenia |
| E1023 | Acute exacerbation of subchronic catatonic schizophrenia |
| E1024 | Acute exacerbation of chronic catatonic schizophrenia |
| E1025 | Catatonic schizophrenia in remission |
| E102z | Catatonic schizophrenia NOS |
| E103. | Paranoid schizophrenia |
| E1030 | Unspecified paranoid schizophrenia |
| E1031 | Subchronic paranoid schizophrenia |
| E1032 | Chronic paranoid schizophrenia |
| E1033 | Acute exacerbation of subchronic paranoid schizophrenia |
| E1034 | Acute exacerbation of chronic paranoid schizophrenia |
| E1035 | Paranoid schizophrenia in remission |
| E103z | Paranoid schizophrenia NOS |
| E104. | Acute schizophrenic episode |
| E105. | Latent schizophrenia |
| E1050 | Unspecified latent schizophrenia |
| E1051 | Subchronic latent schizophrenia |
| E1052 | Chronic latent schizophrenia |
| E1053 | Acute exacerbation of subchronic latent schizophrenia |
| E1054 | Acute exacerbation of chronic latent schizophrenia |
| E1055 | Latent schizophrenia in remission |
| E105z | Latent schizophrenia NOS |
| E106. | Residual schizophrenia |
| E107. | Schizo-affective schizophrenia |
| E1070 | Unspecified schizo-affective schizophrenia |
| E1071 | Subchronic schizo-affective schizophrenia |
| E1072 | Chronic schizo-affective schizophrenia |
| E1073 | Acute exacerbation subchronic schizo-affective schizophrenia |
| E1074 | Acute exacerbation of chronic schizo-affective schizophrenia |
| E1075 | Schizo-affective schizophrenia in remission |
| E107z | Schizo-affective schizophrenia NOS |
| E10y. | Other schizophrenia |
| E10y0 | Atypical schizophrenia |
| E10y1 | Coenesthopathic schizophrenia |
| E10yz | Other schizophrenia NOS |
| E10z. | Schizophrenia NOS |
| E11.. | Affective psychoses |
| E110. | Manic disorder, single episode |
| E1100 | Single manic episode, unspecified |
| E1101 | Single manic episode, mild |
| E1102 | Single manic episode, moderate |
| E1103 | Single manic episode, severe without mention of psychosis |
| E1104 | Single manic episode, severe, with psychosis |
| E1105 | Single manic episode in partial or unspecified remission |
| E1106 | Single manic episode in full remission |
| E110z | Manic disorder, single episode NOS |
| E111. | Recurrent manic episodes |
| E1110 | Recurrent manic episodes, unspecified |
| E1111 | Recurrent manic episodes, mild |
| E1112 | Recurrent manic episodes, moderate |
| E1113 | Recurrent manic episodes, severe without mention psychosis |
| E1114 | Recurrent manic episodes, severe, with psychosis |
| E1115 | Recurrent manic episodes, partial or unspecified remission |
| E1116 | Recurrent manic episodes, in full remission |
| E111z | Recurrent manic episode NOS |
| Eu2.. | [X]Schizophrenia, schizotypal and delusional disorders |
| Eu20. | [X]Schizophrenia |
| Eu200 | [X]Paranoid schizophrenia |
| Eu201 | [X]Hebephrenic schizophrenia |
| Eu202 | [X]Catatonic schizophrenia |
| Eu203 | [X]Undifferentiated schizophrenia |
| Eu204 | [X]Post-schizophrenic depression |
| Eu205 | [X]Residual schizophrenia |
| Eu206 | [X]Simple schizophrenia |
| Eu20y | [X]Other schizophrenia |
| Eu20z | [X]Schizophrenia, unspecified |
| Eu21. | [X]Schizotypal disorder |
| Eu22. | [X]Persistent delusional disorders |
| Eu220 | [X]Delusional disorder |
| Eu221 | [X]Delusional misidentification syndrome |
| Eu222 | [X]Cotard syndrome |
| Eu223 | [X]Paranoid state in remission |
| Eu22y | [X]Other persistent delusional disorders |
| Eu22z | [X]Persistent delusional disorder, unspecified |
| Eu23. | [X]Acute and transient psychotic disorders |
| Eu230 | [X]Acute polymorphic psychot disord without symp of schizoph |
| Eu231 | [X]Acute polymorphic psychot disord with symp of schizophren |
| Eu232 | [X]Acute schizophrenia-like psychotic disorder |
| Eu233 | [X]Other acute predominantly delusional psychotic disorders |
| Eu23y | [X]Other acute and transient psychotic disorders |
| Eu23z | [X]Acute and transient psychotic disorder, unspecified |
| Eu24. | [X]Induced delusional disorder |
| Eu25. | [X]Schizoaffective disorders |
| Eu250 | [X]Schizoaffective disorder, manic type |
| Eu251 | [X]Schizoaffective disorder, depressive type |
| Eu252 | [X]Schizoaffective disorder, mixed type |
| Eu25y | [X]Other schizoaffective disorders |
| Eu25z | [X]Schizoaffective disorder, unspecified |
| Eu26. | [X]Nonorganic psychosis in remission |
| Eu2y. | [X]Other nonorganic psychotic disorders |
| Eu2z. | [X]Unspecified nonorganic psychosis |
| ZV110 | [V]Personal history of schizophrenia |
| E114. | Bipolar affective disorder, currently manic |
| E1140 | Bipolar affective disorder, currently manic, unspecified |
| E1141 | Bipolar affective disorder, currently manic, mild |
| E1142 | Bipolar affective disorder, currently manic, moderate |
| E1143 | Bipolar affect disord, currently manic, severe, no psychosis |
| E1144 | Bipolar affect disord, currently manic,severe with psychosis |
| E1145 | Bipolar affect disord,currently manic, part/unspec remission |
| E1146 | Bipolar affective disorder, currently manic, full remission |
| E114z | Bipolar affective disorder, currently manic, NOS |
| E115. | Bipolar affective disorder, currently depressed |
| E1150 | Bipolar affective disorder, currently depressed, unspecified |
| E1151 | Bipolar affective disorder, currently depressed, mild |
| E1152 | Bipolar affective disorder, currently depressed, moderate |
| E1153 | Bipolar affect disord, now depressed, severe, no psychosis |
| E1154 | Bipolar affect disord, now depressed, severe with psychosis |
| E1155 | Bipolar affect disord, now depressed, part/unspec remission |
| E1156 | Bipolar affective disorder, now depressed, in full remission |
| E115z | Bipolar affective disorder, currently depressed, NOS |
| E116. | Mixed bipolar affective disorder |
| E1160 | Mixed bipolar affective disorder, unspecified |
| E1161 | Mixed bipolar affective disorder, mild |
| E1162 | Mixed bipolar affective disorder, moderate |
| E1163 | Mixed bipolar affective disorder, severe, without psychosis |
| E1164 | Mixed bipolar affective disorder, severe, with psychosis |
| E1165 | Mixed bipolar affective disorder, partial/unspec remission |
| E1166 | Mixed bipolar affective disorder, in full remission |
| E116z | Mixed bipolar affective disorder, NOS |
| E117. | Unspecified bipolar affective disorder |
| E1170 | Unspecified bipolar affective disorder, unspecified |
| E1171 | Unspecified bipolar affective disorder, mild |
| E1172 | Unspecified bipolar affective disorder, moderate |
| E1173 | Unspecified bipolar affective disorder, severe, no psychosis |
| E1174 | Unspecified bipolar affective disorder,severe with psychosis |
| E1175 | Unspecified bipolar affect disord, partial/unspec remission |
| E1176 | Unspecified bipolar affective disorder, in full remission |
| E117z | Unspecified bipolar affective disorder, NOS |
| E11y. | Other and unspecified manic-depressive psychoses |
| E11y0 | Unspecified manic-depressive psychoses |
| E11y1 | Atypical manic disorder |
| E11y2 | Atypical depressive disorder |
| E11y3 | Other mixed manic-depressive psychoses |
| E11yz | Other and unspecified manic-depressive psychoses NOS |
| Eu31. | [X]Bipolar affective disorder |
| Eu310 | [X]Bipolar affective disorder, current episode hypomanic |
| Eu311 | [X]Bipolar affect disorder cur epi manic wout psychotic symp |
| Eu312 | [X]Bipolar affect disorder cur epi manic with psychotic symp |
| Eu313 | [X]Bipolar affect disorder cur epi mild or moderate depressn |
| Eu314 | [X]Bipol aff disord, curr epis sev depress, no psychot symp |
| Eu315 | [X]Bipolar affect dis cur epi severe depres with psyc symp |
| Eu316 | [X]Bipolar affective disorder, current episode mixed |
| Eu317 | [X]Bipolar affective disorder, currently in remission |
| Eu31y | [X]Other bipolar affective disorders |
| Eu31z | [X]Bipolar affective disorder, unspecified |
| 14F2. | H/O: psoriasis |
| M160. | Psoriatic arthropathy |
| M1600 | Psoriasis spondylitica |
| M1601 | Distal interphalangeal psoriatic arthropathy |
| M160z | Psoriatic arthropathy NOS |
| M161. | Other psoriasis |
| M1610 | Psoriasis unspecified |
| M1611 | Psoriasis annularis |
| M1612 | Psoriasis circinata |
| M1613 | Psoriasis diffusa |
| M1614 | Psoriasis discoidea |
| M1615 | Psoriasis geographica |
| M1616 | Guttate psoriasis |
| M1617 | Psoriasis gyrata |
| M1618 | Psoriasis inveterata |
| M1619 | Psoriasis ostracea |
| M161A | Psoriasis palmaris |
| M161B | Psoriasis plantaris |
| M161C | Psoriasis punctata |
| M161D | Pustular psoriasis |
| M161E | Psoriasis universalis |
| M161F | Psoriasis vulgaris |
| M161G | Acrodermatitis continua |
| M161H | Erythrodermic psoriasis |
| M161z | Psoriasis NOS |
| M16y. | Other psoriasis and similar disorders |
| M16y0 | Scalp psoriasis |
| M16z. | Psoriasis and similar disorders NOS |
| Myu30 | [X]Other psoriasis |
| N0452 | Juvenile arthritis in psoriasis |
| Nyu13 | [X]Other psoriatic arthropathies |
| 14F1. | H/O: eczema |
| 26C4. | Nipple eczema |
| F4D30 | Eczematous eyelid dermatitis |
| F5024 | Acute eczematoid otitis extern |
| M102. | Infectious eczematoid dermatitis |
| M111. | Atopic dermatitis/eczema |
| M112. | Infantile eczema |
| M113. | Flexural eczema |
| M114. | Allergic (intrinsic) eczema |
| M119. | Discoid eczema |
| M11A. | Asteatotic eczema |
| M12z1 | Eczema NOS |
| M12z2 | Infected eczema |
| M12z3 | Hand eczema |
| M12z4 | Erythrodermic eczema |
| M1y2. | Gravitational eczema |
| Myu2. | [X]Dermatitis and eczema |
| Myu22 | [X]Exacerbation of eczema |
| 14C4. | H/O: colitis |
| J08z9 | Orofacial Crohn's disease |
| J40.. | Regional enteritis - Crohn's disease |
| J400. | Regional enteritis of the small bowel |
| J4000 | Regional enteritis of the duodenum |
| J4001 | Regional enteritis of the jejunum |
| J4002 | Crohn's disease of the terminal ileum |
| J4003 | Crohn's disease of the ileum unspecified |
| J4004 | Crohn's disease of the ileum NOS |
| J4005 | Exacerbation of Crohn's disease of small intestine |
| J400z | Crohn's disease of the small bowel NOS |
| J401. | Regional enteritis of the large bowel |
| J4010 | Regional enteritis of the colon |
| J4011 | Regional enteritis of the rectum |
| J4012 | Exacerbation of Crohn's disease of large intestine |
| J401z | Crohn's disease of the large bowel NOS |
| J402. | Regional ileocolitis |
| J40z. | Regional enteritis NOS |
| J41.. | Idiopathic proctocolitis |
| J410. | Ulcerative proctocolitis |
| J4100 | Ulcerative ileocolitis |
| J4101 | Ulcerative colitis |
| J4102 | Ulcerative rectosigmoiditis |
| J4103 | Ulcerative proctitis |
| J4104 | Exacerbation of ulcerative colitis |
| J410z | Ulcerative proctocolitis NOS |
| J411. | Ulcerative (chronic) enterocolitis |
| J412. | Ulcerative (chronic) ileocolitis |
| J41y. | Other idiopathic proctocolitis |
| J4212 | Chronic ischaemic enterocolitis |
| J4213 | Chronic ischaemic colitis |
| J42z0 | Ischaemic colitis NOS |
| J4302 | Radiation enterocolitis |
| J4303 | Radiation colitis |
| J4312 | Toxic enterocolitis |
| J4313 | Toxic colitis |
| J4322 | Allergic enterocolitis |
| J4323 | Allergic colitis |
| J4332 | Dietetic enterocolitis |
| J4333 | Dietetic colitis |
| J436. | Microscopic colitis |
| J4360 | Collagenous colitis |
| J4361 | Lymphocytic colitis |
| J437. | Colitis |
| J4z3. | Non-infective colitis NOS |
| J4z5. | Exacerbation of non-infective colitis |
| J4z6. | Indeterminate colitis |
| Jyu4. | [X]Noninfective enteritis and colitis |
| Jyu40 | [X]Other Crohn's disease |
| Jyu41 | [X]Other ulcerative colitis |
| N0310 | Arthropathy in ulcerative colitis |
| N0454 | Juvenile arthritis in ulcerative colitis |
| J08z9 | Orofacial Crohn's disease |
| J40.. | Regional enteritis - Crohn's disease |
| J4002 | Crohn's disease of the terminal ileum |
| J4003 | Crohn's disease of the ileum unspecified |
| J4004 | Crohn's disease of the ileum NOS |
| J4005 | Exacerbation of Crohn's disease of small intestine |
| J400z | Crohn's disease of the small bowel NOS |
| J4012 | Exacerbation of Crohn's disease of large intestine |
| J401z | Crohn's disease of the large bowel NOS |
| Jyu40 | [X]Other Crohn's disease |
| N0311 | Arthropathy in Crohn's disease |
| N0453 | Juvenile arthritis in Crohn's disease |
| 1B75. | Loss of vision |
| 1B77. | Deteriorating vision |
| 2B6B. | O/E - R-eye completely blind |
| 2B6S. | O/E - pinhole R-eye completely blind |
| 2B7B. | O/E - L-eye completely blind |
| 2BBr. | Impaired vision due to diabetic retinopathy |
| 2B7S. | O/E - pinhole L-eye completely blind |
| 6688 | Registered partially sighted |
| 668B. | Poor visual acuity |
| 6689 | Registered blind |
| 668C. | Certificate of vision impairment |
| C245. | Vitamin A deficiency with night blindness |
| E2011 | Hysterical blindness |
| F4041 | Blind hypotensive eye |
| F4042 | Blind hypertensive eye |
| F4653 | After-cataract with vision obscured |
| F4811 | Sudden visual loss |
| F4814 | Other transient visual loss |
| F486. | Night blindness |
| F4860 | Unspecified night blindness |
| F4862 | Hereditary night blindness NOS |
| F4863 | Congenital night blindness NOS |
| F4864 | Acquired night blindness |
| F486z | Night blindness NOS |
| F490. | Blindness, both eyes |
| F4900 | Unspecified blindness both eyes |
| F4901 | Both eyes total visual impairment |
| F4902 | Better eye: near total VI, Lesser eye: unspecified |
| F4903 | Better eye: near total VI, Lesser eye: total VI |
| F4904 | Better eye: near total VI, Lesser eye: near total VI |
| F4905 | Better eye: profound VI, Lesser eye: unspecified |
| F4906 | Better eye: profound VI, Lesser eye: total VI |
| F4907 | Better eye: profound VI, Lesser eye: near total VI |
| F4908 | Better eye: profound VI, Lesser eye: profound VI |
| F4909 | Acquired blindness, both eyes |
| F490z | Blindness both eyes NOS |
| F491. | Better eye: low vision, Lesser eye: profound VI |
| F4910 | One eye blind, one eye low vision |
| F4911 | Better eye: severe VI, Lesser eye: blind, unspecified |
| F4912 | Better eye: severe VI, Lesser eye: total VI |
| F4913 | Better eye: severe VI, Lesser eye: near total VI |
| F4914 | Better eye: severe VI, Lesser eye: profound VI |
| F4915 | Better eye: moderate VI, Lesser eye: blind, unspecified |
| F4916 | Better eye: moderate VI, Lesser eye: total VI |
| F4917 | Better eye: moderate VI, Lesser eye: near total VI |
| F4918 | Better eye: moderate VI, Lesser eye: profound VI |
| F491z | One eye blind, one eye low vision NOS |
| F492. | Low vision, both eyes |
| F4920 | Low vision, both eyes unspecified |
| F4921 | Better eye: severe VI, Lesser eye: low vision unspecified |
| F4922 | Better eye: severe VI, Lesser eye: severe VI |
| F4923 | Better eye: moderate VI, Lesser eye: low vision unspecified |
| F4924 | Better eye: moderate VI, Lesser eye: severe VI |
| F4925 | Better eye: moderate VI, Lesser eye: moderate VI |
| F492z | Low vision, both eyes NOS |
| F493. | Visual loss, both eyes unqualified |
| F494. | Legal blindness USA |
| F495. | Profound impairment, one eye |
| F4950 | Blindness, one eye, unspecified |
| F4951 | Lesser eye: total visual impairment, Better eye: unspecified |
| F4952 | Lesser eye: total VI, Better eye: near normal vision |
| F4953 | Lesser eye: total VI, Better eye: normal vision |
| F4954 | Lesser eye: near total VI, Better eye: unspecified |
| F4955 | Lesser eye: near total VI, Better eye: near normal vision |
| F4956 | Lesser eye: near total VI, Better eye: normal vision |
| F4957 | Lesser eye: profound VI, Better eye: unspecified |
| F4958 | Lesser eye: profound VI, Better eye: near normal vision |
| F4959 | Lesser eye: profound VI, Better eye: normal vision |
| F495A | Acquired blindness, one eye |
| F495z | Profound impairment one eye NOS |
| F496. | Low vision, one eye |
| F4960 | Low vision, one eye, unspecified |
| F4961 | Lesser eye: severe VI, Better eye: unspecified |
| F4962 | Lesser eye: severe VI, Better eye: near normal vision |
| F4963 | Lesser eye: severe VI, Better eye: normal vision |
| F4964 | Lesser eye: moderate VI, Better eye: unspecified |
| F4965 | Lesser eye: moderate VI, Better eye: near normal vision |
| F4966 | Lesser eye: moderate VI, Better eye: normal vision |
| F496z | Low vision, one eye NOS |
| F49y. | Visual loss, one eye, unqualified |
| F49z. | Visual loss NOS |
| F4H73 | Cortical blindness |
| FyuL. | [X]Visual disturbances and blindness |
| P3410 | Congenital corneal opacity with visual deficit |
| P3411 | Congenital corneal opacity without visual deficit |
| H13.. | Chronic sinusitis |
| H130. | Chronic maxillary sinusitis |
| H131. | Chronic frontal sinusitis |
| H132. | Chronic ethmoidal sinusitis |
| H133. | Chronic sphenoidal sinusitis |
| H134. | Fistula of nasal sinus |
| H135. | Recurrent sinusitis |
| H13y. | Other chronic sinusitis |
| H13y0 | Chronic pansinusitis |
| H13y1 | Pansinusitis |
| H13yz | Other chronic sinusitis NOS |
| H13z. | Chronic sinusitis NOS |
| Hyu22 | [X]Other chronic sinusitis |
| J0835 | Oroantral fistula |
| 13Z4E | Learning difficulties |
| 9HB3. | Learning disabilities health assessment |
| 9HB5. | Learning disabilities annual health assessment |
| E3... | Mental retardation |
| E30.. | Mild mental retardation, IQ in range 50-70 |
| E31.. | Other specified mental retardation |
| E310. | Moderate mental retardation, IQ in range 35-49 |
| E311. | Severe mental retardation, IQ in range 20-34 |
| E312. | Profound mental retardation with IQ less than 20 |
| E31z. | Other specified mental retardation NOS |
| E3y.. | Other specified mental retardation |
| E3z.. | Mental retardation NOS |
| Eu7.. | [X]Mental retardation |
| Eu70. | [X]Mild mental retardation |
| Eu700 | [X]Mld mental retard with statement no or min impairm behav |
| Eu701 | [X]Mld mental retard sig impairment behav req attent/treatmt |
| Eu70y | [X]Mild mental retardation, other impairments of behaviour |
| Eu70z | [X]Mild mental retardation without mention impairment behav |
| Eu71. | [X]Moderate mental retardation |
| Eu710 | [X]Mod mental retard with statement no or min impairm behav |
| Eu711 | [X]Mod mental retard sig impairment behav req attent/treatmt |
| Eu71y | [X]Mod retard oth behav impair |
| Eu71z | [X]Mod mental retardation without mention impairment behav |
| Eu72. | [X]Severe mental retardation |
| Eu720 | [X]Sev mental retard with statement no or min impairm behav |
| Eu721 | [X]Sev mental retard sig impairment behav req attent/treatmt |
| Eu72y | [X]Severe mental retardation, other impairments of behaviour |
| Eu72z | [X]Sev mental retardation without mention impairment behav |
| Eu73. | [X]Profound mental retardation |
| Eu730 | [X]Profound ment retrd wth statement no or min impairm behav |
| Eu731 | [X]Profound ment retard sig impairmnt behav req attent/treat |
| Eu73y | [X]Profound mental retardation, other impairments of behavr |
| Eu73z | [X]Prfnd mental retardation without mention impairment behav |
| Eu7y. | [X]Other mental retardation |
| Eu7y0 | [X]Oth mental retard with statement no or min impairm behav |
| Eu7y1 | [X]Oth mental retard sig impairment behav req attent/treatmt |
| Eu7yy | [X]Other mental retardation, other impairments of behaviour |
| Eu7yz | [X]Other mental retardation without mention impairment behav |
| Eu7z. | [X]Unspecified mental retardation |
| Eu7z0 | [X]Unsp mental retard with statement no or min impairm behav |
| Eu7z1 | [X]Unsp mentl retard sig impairment behav req attent/treatmt |
| Eu7zy | [X]Unspecified mental retardatn, other impairments of behav |
| Eu7zz | [X]Unsp mental retardation without mention impairment behav |
| Eu814 | [X]Moderate learning disability |
| Eu815 | [X]Severe learning disability |
| Eu816 | [X]Mild learning disability |
| Eu817 | [X]Profound learning disability |
| Eu81z | [X]Developmental disorder of scholastic skills, unspecified |
| Z7CD2 | Learning difficulties |
| 1467 | H/O: anorexia nervosa |
| E271. | Anorexia nervosa |
| E2751 | Bulimia (non-organic overeating) |
| Eu500 | [X]Anorexia nervosa |
| Eu501 | [X]Atypical anorexia nervosa |
| Eu502 | [X]Bulimia nervosa |
| Eu503 | [X]Atypical bulimia nervosa |
| A115. | Tuberculous bronchiectasis |
| H34.. | Bronchiectasis |
| H340. | Recurrent bronchiectasis |
| H341. | Post-infective bronchiectasis |
| H34z. | Bronchiectasis NOS |
| P861. | Congenital bronchiectasis |
| 147F. | History of Parkinson's disease |
| 297A. | O/E - Parkinsonian tremor |
| 2987 | O/E -Parkinson flexion posture |
| 2994 | O/E-festination-Parkinson gait |
| A94y1 | Syphilitic parkinsonism |
| Eu023 | [X]Dementia in Parkinson's disease |
| F11x9 | Cerebral degeneration in Parkinson's disease |
| F12.. | Parkinson's disease |
| F120. | Paralysis agitans |
| F121. | Parkinsonism secondary to drugs |
| F123. | Postencephalitic parkinsonism |
| F124. | Vascular parkinsonism |
| F12W. | Secondary parkinsonism due to other external agents |
| F12X. | Secondary parkinsonism, unspecified |
| F12z. | Parkinson's disease NOS |
| F1303 | Parkinsonism with orthostatic hypotension |
| Fyu20 | [X]Other drug-induced secondary parkinsonism |
| Fyu21 | [X]Other secondary parkinsonism |
| Fyu22 | [X]Parkinsonism in diseases classified elsewhere |
| Fyu29 | [X]Secondary parkinsonism, unspecified |
| Fyu2B | [X]Secondary parkinsonism due to other external agents |
| 666A. | Multiple sclerosis review |
| 666B. | Multiple sclerosis multidisciplinary review |
| 8CS1. | Multiple sclerosis care plan agreed |
| F20.. | Multiple sclerosis |
| F200. | Multiple sclerosis of the brain stem |
| F201. | Multiple sclerosis of the spinal cord |
| F202. | Generalised multiple sclerosis |
| F203. | Exacerbation of multiple sclerosis |
| F204. | Benign multiple sclerosis |
| F205. | Malignant multiple sclerosis |
| F206. | Primary progressive multiple sclerosis |
| F207. | Relapsing and remitting multiple sclerosis |
| F208. | Secondary progressive multiple sclerosis |
| F20z. | Multiple sclerosis NOS |
| 141E. | History of hepatitis B |
| 65Q7. | Viral hepatitis carrier |
| A702. | Viral hepatitis B with coma |
| A7020 | Acute hep B with delta-agent (coinfection) with hep coma |
| A703. | Viral (serum) hepatitis B |
| A7030 | Acute hep B with delta-agent (coinfectn) without hep coma |
| A7040 | Viral hepatitis C with coma |
| A7050 | Viral hepatitis C without mention of hepatic coma |
| A7051 | Acute delta-(super)infection of hepatitis B carrier |
| A7054 | Hepatitis non A non B |
| A707. | Chronic viral hepatitis |
| A7070 | Chronic viral hepatitis B with delta-agent |
| A7071 | Chronic viral hepatitis B without delta-agent |
| A7072 | Chronic viral hepatitis C |
| A707X | Chronic viral hepatitis, unspecified |
| A70z0 | Hepatitis C |
| AE23. | Sequelae of viral hepatitis |
| AyuB1 | [X]Other chronic viral hepatitis |
| AyuB2 | [X]Chronic viral hepatitis, unspecified |
| AyuJ9 | [X]Sequelae of viral hepatitis |
| Q409. | Congenital viral hepatitis |
| Q4091 | Congenital hepatitis B infection |
| Q409y | Other specified congenital viral hepatitis |
| Q409z | Congenital viral hepatitis NOS |
| ZV026 | [V]Viral hepatitis carrier |
| ZV02B | [V]Hepatitis B carrier |
| ZV02C | [V]Hepatitis C carrier |
| G8522 | Oesophageal varices in cirrhosis of the liver |
| J601. | Subacute necrosis of liver |
| J6010 | Subacute hepatic failure |
| J6011 | Subacute hepatitis - noninfective |
| J6012 | Subacute yellow atrophy |
| J601z | Subacute necrosis of liver NOS |
| J60z. | Acute and subacute liver necrosis NOS |
| J61.. | Cirrhosis and chronic liver disease |
| J614. | Chronic hepatitis |
| J6140 | Chronic persistent hepatitis |
| J6141 | Chronic active hepatitis |
| J6142 | Chronic aggressive hepatitis |
| J6143 | Recurrent hepatitis |
| J6144 | Chronic lobular hepatitis |
| J614y | Chronic hepatitis unspecified |
| J614z | Chronic hepatitis NOS |
| J615. | Cirrhosis - non alcoholic |
| J6150 | Unilobular portal cirrhosis |
| J6151 | Multilobular portal cirrhosis |
| J6152 | Mixed portal cirrhosis |
| J6153 | Diffuse nodular cirrhosis |
| J6154 | Fatty portal cirrhosis |
| J6155 | Hypertrophic portal cirrhosis |
| J6156 | Capsular portal cirrhosis |
| J6157 | Cardiac portal cirrhosis |
| J6158 | Juvenile portal cirrhosis |
| J6159 | Pigmentary portal cirrhosis |
| J615A | Pipe-stem portal cirrhosis |
| J615B | Toxic portal cirrhosis |
| J615C | Xanthomatous portal cirrhosis |
| J615D | Bacterial portal cirrhosis |
| J615E | Cardituberculous cirrhosis |
| J615F | Syphilitic portal cirrhosis |
| J615G | Zooparasitic portal cirrhosis |
| J615H | Infectious cirrhosis NOS |
| J615y | Portal cirrhosis unspecified |
| J615z | Non-alcoholic cirrhosis NOS |
| J616. | Biliary cirrhosis |
| J6160 | Primary biliary cirrhosis |
| J6161 | Secondary biliary cirrhosis |
| J6162 | Biliary cirrhosis of children |
| J616z | Biliary cirrhosis NOS |
| J617. | Alcoholic hepatitis |
| J6170 | Chronic alcoholic hepatitis |
| J61y. | Other non-alcoholic chronic liver disease |
| J61y0 | Chronic yellow liver atrophy |
| J61y1 | Non-alcoholic fatty liver |
| J61y2 | Hepatosplenomegaly |
| J61y3 | Portal fibrosis without cirrhosis |
| J61y4 | Hepatic fibrosis |
| J61y5 | Hepatic sclerosis |
| J61y6 | Hepatic fibrosis with hepatic sclerosis |
| J61y7 | Steatosis of liver |
| J61y8 | Nonalcoholic steatohepatitis |
| J61yz | Other non-alcoholic chronic liver disease NOS |
| J61z. | Chronic liver disease NOS |
| J623. | Portal hypertension |
| J624. | Hepatorenal syndrome |
| J62y. | Other sequelae of chronic liver disease |
| J62z. | Liver abscess and chronic liver disease causing sequelae NOS |
| J630. | Chronic passive liver congestion |
| Jyu71 | [X]Other and unspecified cirrhosis of liver |
| PB62. | Congenital cystic liver disease |
| PB620 | Congenital polycystic liver disease |

**Table S4 Results of variable selection in SAIL cohort (using fast backward selection method)**(3)

| **Variable name** | **p-value** | **Status of selection** |
| --- | --- | --- |
| Eating disorders | 0.92 | Not selected |
| Multiple Sclerosis | 0.89 | Not selected |
| Schizophrenia or bipolar disorder | 0.94 | Not selected |
| Tuberculosis | 0.92 | Not selected |
| Diabetes | 0.73 | Not selected |
| Viral hepatitis | 0.79 | Not selected |
| Diverticular disease | 0.50 | Not selected |
| Connective tissue disease | 0.71 | Not selected |
| Chronic kidney disease | 0.54 | Not selected |
| Asbestosis | 0.42 | Not selected |
| Deafness | 0.40 | Not selected |
| Glaucoma | 0.30 | Not selected |
| Anxiety | 0.36 | Not selected |
| Heart failure | 0.23 | Not selected |
| Parkinson’s disease | 0.25 | Not selected |
| Migraine | 0.24 | Not selected |
| Bronchiectasis | 0.14 | Not selected |
| Depression | 0.25 | Not selected |
| Inflammatory Bowel Disease | 0.16 | Not selected |
| Chronic liver disease | 0.17 | Not selected |
| Thyroid disease | 0.12 | Not selected |
| Psoriasis or Eczema | 0.10 | Not selected |
| Irritable Bowel Syndrome | 0.09 | Not selected |
| Blindness and low vision | 0.09 | Not selected |
| Asthma | 0.08 | Not selected |
| Epilepsy | 0.07 | Not selected |
| Education class | 0.09 | Not selected |
| Psychoactive substance misuse | 0.05 | Not selected |
| Treated constipation | 0.02 | Not selected |
| Prostate disorders | 0.02 | Not selected |
| Atrial Fibrillation | 0.01 | Not selected |
| Chronic sinusitis | 0.01 | Not selected |
|  |  |  |
|  |  |  |
| Stroke/Transient Ischaemic Attack | 0.009 | Selected |
| Coronary Heart Disease | 0.008 | Selected |
| Dementia | <0.001 | Selected |
| Alcohol misuse | <0.001 | Selected |
| Chronic Obstructive Pulmonary Disease | <0.001 | Selected |
| Hypertension | <0.001 | Selected |
| Painful condition | <0.001 | Selected |
| Peripheral vascular disease | <0.001 | Selected |
| History of previous cancer | <0.001 | Selected |
| History of previous pneumonia | <0.001 | Selected |
| Age-continuous | <0.001 | Selected |
| Sex-Male | 0.001 | Selected |
| WIMD_2011_Quintile5 | Reference | Selected |
| WIMD_2011_Quintile4 | 0.005 | Selected |
| WIMD_2011_Quintile3 | <0.001 | Selected |
| WIMD_2011_Quintile2 | <0.001 | Selected |
| WIMD_2011_Quintile1 | <0.001 | Selected |
| Smoking status: non-smoker | Reference | Selected |
| Smoking status: ex-smoker | <0.001 | Selected |
| Smoking status: current smoker | <0.001 | Selected |
| Family history of lung cancer | <0.001 | Selected |
| Body mass index | <0.001 | Selected |

WIMD=Wales index for multiple deprivation; WIMD is area-based measures of socio-economic status(4); P-value<0.01 was taken as significance level for selecting variables into the model.

**Table S5 Statistical interaction testing between selected variables and smoking status**

| Interaction | p-value |
| --- | --- |
| Age: smoking status | 0.12 |
| Sex: smoking status | 0.09 |
| WIMD: smoking status | 0.23 |
| Alcohol problem: Smoking status | 0.52 |
| COPD: smoking status | 0.66 |
| CHD: smoking status | 0.15 |
| Peripheral vascular disease: smoking status | 0.17 |
| Stroke/TIA: smoking status | 0.063 |
| Dementia: smoking status | 0.77 |
| Hypertension: smoking status | 0.10 |
| Painful condition: smoking status | 0.83 |
| Cancer history: smoking status | 0.07 |
| Pneumonia: smoking status | 0.08 |
| Family history of lung cancer: smoking status | 0.65 |
| **BMI: smoking status** | **0.01** |

**Table S6 Sensitivity analyses based on using hospital admission data (in addition to GP data) for calculating LTC prevalence.**

| SAIL cohort. N=530069; Lung cancer incidence at 6 years Cases=5923 | | | | |
| --- | --- | --- | --- | --- |
|  | ALIGNED | Ever smoker | PLCO | LLP |
| AUC with 95% CI | 82.5% (82%-83%) | 69.8% (69.4%-70.2%) | 82.4% (81.9%-82.9%) | 75.9% (75.2%-76.5%) |
| UK Biobank cohort. N=137,918 ; Lung cancer incidence at 6 years=656 | | | | |
| AUC with 95% CI | 74.9% (72.7%-77.0%) | 60.2% (58.3%-62.2%) | 82.7% (80.9%-84.5%) | 81.1% (792%-83.1%) |

**Table S7 Sensitivity Analysis in SAIL data comparing AUC performance of various lung cancer risk cores for 5-year lung cancer incidence.**

| SAIL cohort N=530069; Lung cancer incidence at 5 years Cases=5177 | | | | |
| --- | --- | --- | --- | --- |
|  | ALIGNED | Ever smoker | PLCOM2012 | LLPv2 |
| AUC with 95% CI | 80.3% (79.8%-80.9%) | 69.8% (69.4%-70.2%) | 80.1% (79.6%-80.7%) | 69.4% (68.7%-70.1%) |

**Table S8 Results from internal validation in the SAIL cohort by splitting data into 10 equal sub-groups.**

| Sub-group | Number of lung cancer cases | AUC |
| --- | --- | --- |
| T1 N=52898 | 536 | 79.2% (77.4%-81.0%) |
| T2 N=52652 | 577 | 81.0% (79.5%-82.5%) |
| T3 N=53128 | 613 | 81.6% 980.0%-83.1%) |
| T4 N=52590 | 585 | 79.9% (78.2%-81.6%) |
| T5 N=53370 | 589 | 80.7% (79%-82.3%) |
| T6 N=53218 | 610 | 81.4% (79.9%-82.9%) |
| T7 N=52918 | 593 | 79.1% (77.5%-80.8%) |
| T8 N=53259 | 617 | 80.8% (79.2%-82.4%) |
| T9 N=52976 | 594 | 81.6% (80.1%-83.1%) |
| T10 N=53060 | 609 | 80.6% (79.0%-82.1%) |

**Table S9 Results from sub-group analysis based on demographic characteristics and smoking status in SAIL cohort.**

| Sub-group | N | Number of lung cancer cases | AUC |
| --- | --- | --- | --- |
| Females | 273062 | 2768 | 81.1% (80.4%-81.8%) |
| Males | 257007 | 3155 | 79.6% (78.9%-80.3%) |
| Never smokers | 267228 | 666 | 70.4% (68.4%-72.4%) |
| Ex-smokers | 116187 | 1321 | 71.2% (69.8%-72.6% |
| Current smokers | 146654 | 3936 | 67.1% (66.3%-68%) |
| 55-65 | 302725 | 2307 | 80.4% (79.6%-81.2%) |
| 66-75 | 256367 | 3930 | 78.7% (78%-79.3%) |

**References**

1. Atkinson MD, Kennedy JI, John A, Lewis KE, Lyons RA, Brophy ST, et al. Development of an algorithm for determining smoking status and behaviour over the life course from UK electronic primary care records. BMC Med Inform Decis Mak. 2017;17(1).

2. Hanlon P, Jani BD, Nicholl B, Lewsey J, McAllister DA, Mair FS. Associations between multimorbidity and adverse health outcomes in UK Biobank and the SAIL Databank: A comparison of longitudinal cohort studies. PLoS Med. 2022 Mar 7;19(3):e1003931.

3. Fridman J, Hastie T, Tibshirani R, Al E. Package “glmnet” [Internet]. 2018. p. 1–23. Available from: https://cran.r-project.org/web/packages/glmnet/glmnet.pdf

4. Welsh Government. Welsh Index of Multiple Deprivation [Internet]. 2019. Available from: https://gov.wales/sites/default/files/statistics-and-research/2019-11/welsh-index-multiple-deprivation-guidance.pdf
